# SensEnhance: A Genetic Circuit for Converting Endogenous Mammalian Gene Signals Into High‐Dynamic‐Range Genetic Readouts

**DOI:** 10.1002/advs.78654

**Published:** 2026-11-09

**Authors:** Miguel Baños, Stanislav Anastassov, Payman Goodarzi, Mustafa Khammash, Wilfried Weber

**Affiliations:** ^1^ INM – Leibniz Institute for New Materials Saarbrücken Germany; ^2^ Department of Materials Science and Engineering Saarland University Saarbrücken Germany; ^3^ Department of Biosystems Science and Engineering (D‐BSSE) ETH Zurich Basel Switzerland

**Keywords:** biosensing, genetic circuits, mechanotransduction, synthetic biology

## Abstract

Integrating synthetic gene circuits with endogenous mammalian gene expression programs enables genetic readouts of physiologically relevant cellular states. However, the performance of these readouts is often constrained by sensing promoters with low maximal output, modest fold changes, and high basal activity. Here, we use mathematical modeling and analysis to devise SensEnhance, a generalizable genetic circuit architecture that converts leaky, low‐dynamic‐range promoter signals into tightly controlled, high‐dynamic‐range outputs. SensEnhance combines sequestration‐mediated suppression below a tunable response threshold with feedback‐supported amplification above it. We implement SensEnhance through split intein‐mediated protein trans‐splicing and experimentally validate its predicted input–output behavior and threshold tunability. We apply SensEnhance to a YAP/TAZ‐responsive mechanosensing promoter and stably integrate the circuit into NIH/3T3 fibroblasts, increasing output fold change between soft and stiff tissue‐like hydrogels from 2.6‐fold in the promoter‐only configuration to over 20‐fold using SensEnhance. When coupled to a STAT3‐responsive promoter showing a 2.5‐fold change in response to IL‐6, SensEnhance increases this fold change to 14‐fold, improving cytokine sensing and supporting its generic applicability across signaling pathways. These results establish SensEnhance as a modular strategy for improving the dynamic range and tunability of mammalian genetic biosensors.

## Graphical abstract

Here, we present SensEnhance, a modular genetic circuit that converts leaky, low‐dynamic‐range promoter signals into tightly controlled, high‐dynamic‐range outputs. Applied to mechanosensing and IL‐6‐responsive promoters, SensEnhance increases fold changes from 2.6‐ and 2.5‐fold in promoter‐only configurations to over 20‐ and 14‐fold, respectively. SensEnhance may serve as a high‐performance genetic analog of a threshold amplifier for biosensing.

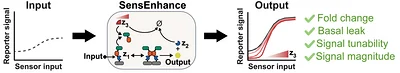

## 1 Introduction

Interfacing synthetic gene circuits with endogenous mammalian gene signals enables genetically encoded readouts of cellular state and environmental cues. These interfaces provide a basis for cell‐based biosensors that convert endogenous signaling activity into measurable outputs [1, 2, 3]. Many cell‐based biosensors rely on endogenous or synthetic sensing promoters whose activity changes in response to a cellular cue. In these systems, natural or engineered receptors detect an input signal and activate downstream signaling cascades, leading to transcription factor binding at specific promoter elements and induction of gene expression [4]. In recent years, remarkable progress has been made in designing and implementing gene switches based on endogenous pathways that activate in response to external [5, 6, 7, 8, 9] and internal signals [10, 11, 12]. However, the performance of these biosensors is often constrained by the low maximal output, modest induction, and high basal activity of mammalian sensing promoters [13, 14, 15].

Strategies to improve promoter performance include engineering promoter architecture [16, 17] or optimizing and overexpressing components of the associated signaling pathway [18, 19]. Although effective, these approaches are often promoter‐ or pathway‐specific, require iterative optimization, and may perturb the endogenous signaling context that the circuit aims to read. Recent efforts have focused on designing generalizable gene network architectures to improve promoter performance. These approaches represent important and promising advances, showing that promoter activity can be processed downstream through amplification [20, 21, 22], feedback [23, 24, 25], thresholding and filtering [21, 24], logic integration [26, 27] and protein‐level regulation [10]. At the same time, they illustrate that improving biosensor performance often involves architecture‐specific trade‐offs. Amplification and positive feedback can increase output magnitude, but also amplify basal activity from weak or leaky sensing promoters. Promoter‐level thresholding based on competitive DNA binding can sharpen responses, but because filtering and activation occur at the same promoter, threshold tuning remains linked to promoter architecture and maximal expression. In addition, some architectures are optimized for switch‐like ON/OFF output, limiting graded reporting across a desired biological input range. Finally, multilayer circuits, CRISPR‐based regulatory modules or large regulatory payloads can increase implementation complexity and construct size, complicating delivery and stable genomic integration required for long‐term use of engineered cells.

An ideal genetic circuit for enhancing sensing promoters should therefore meet several requirements. First, it should act downstream of the sensing promoter, preserving the upstream signaling pathway and core sensing element. Second, it should suppress basal output in the unstimulated state while maintaining strong output when the relevant signal is present. Third, it should increase the fold change between low and high input states, preserve graded information across the relevant input range, and allow the operating range to be tuned to the biological range of interest. Finally, it should be modular and generalizable, while remaining compact enough for stable genomic integration and long‐term use in engineered cells.

Aiming to fulfill these requirements, we developed SensEnhance, a modular genetic circuit architecture that acts downstream of sensing promoters to convert modest promoter activity into high‐dynamic‐range outputs. SensEnhance combines sequestration‐based suppression below a tunable threshold with feedback‐supported amplification above it, thereby reducing low‐input output while maintaining strong, graded expression at higher input levels. By shifting this threshold, the operating range can be adjusted over selected biological input levels. We first theoretically develop the circuit architecture, analyze its operating principles, and experimentally validate its behavior using constitutively controlled modules. We then apply SensEnhance to matrix stiffness sensing. Matrix mechanics are central regulators of tissue homeostasis [28] and disease progression [29, 30], yet genetic readouts of extracellular mechanics often lack sufficient dynamic range to distinguish relevant mechanical states. Using 8xGTIIC, a YAP/TAZ‐responsive promoter widely used to monitor mechanotransduction [31], we generated a stable NIH/3T3 fibroblast cell line that reports static, induced and spatial differences in hydrogel stiffness with greater dynamic range than the mechanosensing promoter alone. Finally, to evaluate whether SensEnhance can serve as a general strategy for improving sensing promoter performance, we coupled it to a STAT3‐responsive promoter reporting IL‐6/JAK/STAT3 signaling and showed improved cytokine sensing. Together, these results establish SensEnhance as a versatile, modular strategy for converting low‐dynamic‐range mammalian sensing promoters into tunable, high‐performance genetic interfaces for biosensing.

## 2 Results

### 2.1 A Sequestration‐Based Architecture for Decoupling Basal Suppression from High‐Gain Amplification

To define the design principles underlying SensEnhance, we first analyzed how basal promoter activity constrains the input‐output behavior of promoter‐based biosensors. We began with the simplest sensor architecture, in which a responsive promoter directly drives reporter expression. At steady state, the reporter output r can be approximated as

(1) $$r_{ss}=\frac{\alpha +u(s)}{\gamma },$$

where α denotes the basal leak rate, u(s) is the input‐dependent production rate induced by the sensed signal s, and γ is the effective degradation or dilution rate. In this architecture, basal and induced expression contribute additively to the output. Consequently, any increase in promoter strength or downstream gain elevates both low‐input and high‐input output, limiting discrimination between basal and induced states (Figure 1a).

**FIGURE 1:**
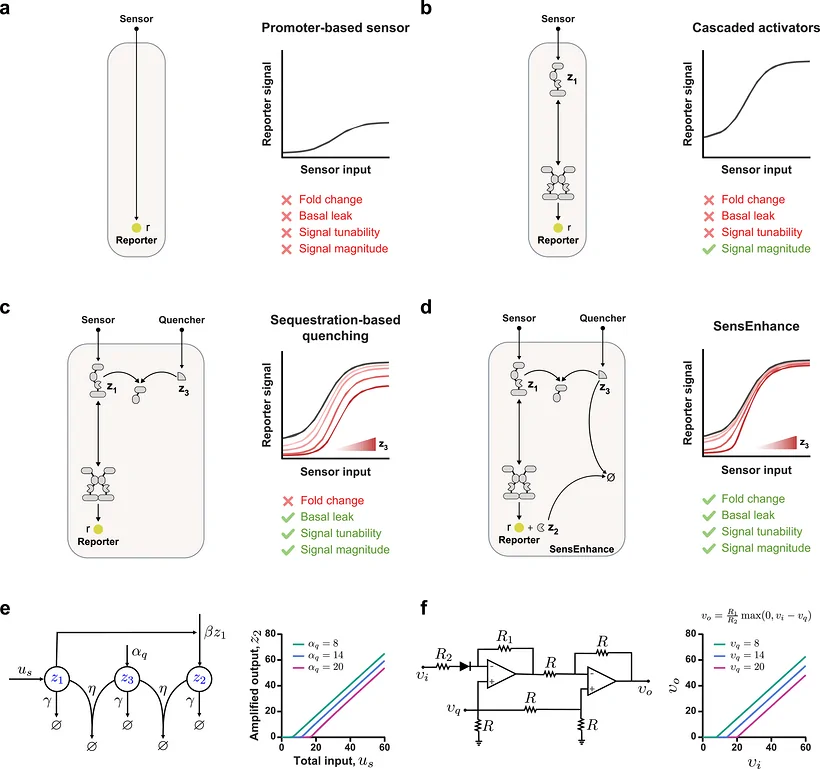
A sequestration‐based architecture for decoupling basal suppression from high‐gain amplification. **(a** **)** Promoter‐based sensor in which a sensing promoter directly drives reporter expression (r). Basal leak and input‐dependent promoter activity contribute additively to reporter output, limiting fold change, signal tunability, and signal magnitude. **(b** **)** Cascaded‐activator architecture in which the sensing promoter drives an activator ($z_{1}$), which activates downstream reporter expression. In the illustrated configuration, this increases signal magnitude, but also propagates and amplifies upstream basal activity, limiting fold change improvement and response tunability. **(c** **)** Sequestration‐based quenching architecture in which a suppressive species ($z_{3}$) stoichiometrically binds the activator ($z_{1}$), reducing the free activator pool available for reporter expression. This suppresses basal output and enables threshold tuning through $z_{3}$ abundance, but continued activator attenuation can limit maximal output and fold change improvement. **(d** **)** SensEnhance builds on the sequestration‐based architecture in **c** by coupling expression of a competing species ($z_{2}$) to the output promoter together with the reporter (r). Below the threshold imposed by $z_{3}$ abundance, $z_{3}$ sequesters the activator ($z_{1}$) and suppresses reporter expression. Above this threshold, output‐driven $z_{2}$ sequesters $z_{3}$, reducing the amount available to inactivate $z_{1}$ and thereby supporting strong reporter expression. This combines reduced basal output, improved fold change, a tunable response range and high signal magnitude. **(e** **)** Simplified SensEnhance circuit illustrating a suppression regime and an amplification regime. The threshold between these regimes can be tuned by selecting $\alpha_{q}$. **(f** **)** The behavior of the SensEnhance biological circuit is analogous, although not identical, to a threshold amplifier whose output remains zero below the threshold and switches to linear amplification above it. Curves in **(a**–**d)** were generated from Equations (1), (4), (5), (6)–(8), and (9)–(12), using u(s)=s/(1+s) and $h\left(z_{1}\right)=Vz_{1}^{n}/\left(K^{n}+z_{1}^{n}\right)$, with γ=1, η=10, V=4, K=0.5, n=1.5, α=0.15, $\alpha_{1}=0.15$, $\alpha_{2}=\alpha_{r}=0.05$ and $\alpha_{3}=0$. Curves in **e** were generated from Equation (13) and Equations (14)–(16), with β=2.2, γ=1.35, η=5 and $\alpha_{q}=8,14,20$. Responses in **f** were generated from $v_{o}=\left(R_{1}/R_{2}\right)\max\left(0,v_{i}-v_{q}\right)$, with $R_{1}/R_{2}=1.21$ and $v_{q}=8,14,20$.

A common strategy to increase output magnitude is transcriptional amplification through cascaded activators. In such architectures, the sensing promoter drives expression of a synthetic transcription factor $z_{1}$, which subsequently activates a downstream reporter promoter through a nonlinear activation function $h(z_{1})$:

(2) $$\begin{array}{cc} {\dot{z}}_{1} & =\alpha_{1}+u\left(s\right)-\gamma z_{1}, \end{array}$$

(3) $$\begin{array}{cc} \dot{r} & =\alpha_{r}+h\left(z_{1}\right)-\gamma r. \end{array}$$

At steady state,

(4) $$\begin{array}{cc} z_{1,ss} & =\frac{\alpha_{1}+u\left(s\right)}{\gamma }, \end{array}$$

(5) $$\begin{array}{cc} r_{ss} & =\frac{\alpha_{r}+h\left(z_{1,ss}\right)}{\gamma }. \end{array}$$

Transcriptional cascades can increase signal magnitude but may also increase background output. Basal expression generated upstream propagates through the cascade and can itself be amplified by the downstream nonlinear activation. Amplification alone therefore does not ensure low background output (Figure 1b).

To address this shortcoming, we sought an architecture capable of selectively suppressing weak‐input activity before amplification occurs. To achieve this, we introduced a sequestration layer that dynamically controls the pool of active transcription factor available for reporter activation. In this topology, the activator $z_{1}$ interacts stoichiometrically with a quencher species $z_{3}$, forming an inactive complex that reduces the concentration of free activator available for downstream transcriptional activation.

The resulting dynamics are

(6) $$\begin{array}{cc} {\dot{z}}_{1} & =\alpha_{1}+u\left(s\right)-\eta z_{1}z_{3}-\gamma z_{1}, \end{array}$$

(7) $$\begin{array}{cc} {\dot{z}}_{3} & =\alpha_{3}+q\left(c\right)-\eta z_{1}z_{3}-\gamma z_{3}, \end{array}$$

(8) $$\begin{array}{cc} \dot{r} & =\alpha_{r}+h\left(z_{1}\right)-\gamma r, \end{array}$$

where η denotes the sequestration rate and q(c) defines an externally tunable input controlling production of the quencher species.

In this architecture, reporter activation depends on the free activator pool rather than total activator abundance. At low‐input levels, sequestration dominates and most activator molecules are rendered inactive, thereby suppressing reporter expression. As input increases, the sequestration layer progressively saturates, allowing free activator levels to rise. The circuit therefore introduces a tunable activation threshold through stoichiometric competition, with the threshold position set by the abundance of the quencher species (Figure 1c). By adjusting quencher expression, the threshold can be shifted to the input range at which reporter activation is desired, without modifying the sensing promoter itself. This allows the same sensing promoter to be configured either to suppress basal leak or to restrict reporter activation to high‐magnitude input states.

However, sequestration‐based thresholding alone introduces a new trade‐off. Although it suppresses basal activity, the quencher species continues to attenuate activator availability even at large inputs, thereby reducing maximal output and limiting overall amplification.

To overcome this limitation, we extended the sequestration architecture by coupling the output stage back to the quenching layer. In the resulting circuit, termed SensEnhance, the output promoter drives expression not only of the reporter r, but also of an additional species $z_{2}$ capable of sequestering the quencher species $z_{3}$. Once free $z_{1}$ activates the output promoter, this promoter produces $z_{2}$ in addition to the reporter. As $z_{2}$ accumulates, it depletes the quencher pool, leaving less $z_{3}$ available to sequester $z_{1}$. This increases the pool of active $z_{1}$ and further enhances reporter activation (Figure 1d).

The complete circuit is described by

(9) $$\begin{array}{cc} {\dot{z}}_{1} & =\alpha_{1}+u\left(s\right)-\eta z_{3}z_{1}-\gamma z_{1}, \end{array}$$

(10) $$\begin{array}{cc} {\dot{z}}_{2} & =\alpha_{2}+h\left(z_{1}\right)-\eta z_{3}z_{2}-\gamma z_{2}, \end{array}$$

(11) $$\begin{array}{cc} {\dot{z}}_{3} & =\alpha_{3}+q\left(c\right)-\eta z_{3}\left(z_{1}+z_{2}\right)-\gamma z_{3}, \end{array}$$

(12) $$\begin{array}{cc} \dot{r} & =\alpha_{r}+h\left(z_{1}\right)-\gamma r. \end{array}$$

Unlike conventional transcriptional positive feedback, amplification in SensEnhance arises indirectly through feedback‐mediated depletion of the inhibitory sequestration layer. The circuit switches between two operating regimes. Below threshold, the quencher species remains abundant and SensEnhance acts in a suppressive mode, strongly attenuating reporter activation. Above threshold, output‐driven depletion of the quencher pool relieves inhibition and shifts the circuit into an amplifying mode, enabling strong reporter activation.

SensEnhance therefore combines threshold‐mediated suppression of weak‐input activity with feedback‐supported amplification at high input levels, effectively decoupling basal suppression from high‐gain output amplification.

### 2.2 Analytical Characterization of the SensEnhance Architecture

To understand the operating principles of SensEnhance independently of implementation‐specific nonlinearities, we analyzed a simplified model in which transcriptional activation is approximated as linear. This simplified description preserves the core sequestration and feedback structure while allowing analytical characterization of the circuit behavior.

To explicitly distinguish basal leak from regulated promoter activity, we write the total input to the activator arm as

(13) $$u_{s}=\ell +u\left(s\right),$$

where ℓ denotes basal leak from the sensing promoter and u(s) denotes the signal‐dependent contribution. Thus, $u_{s}$ represents the effective total production rate of the activator species $z_{1}$.

The simplified dynamics are

(14) $$\begin{array}{cc} {\dot{z}}_{1} & =u_{s}-\eta z_{1}z_{3}-\gamma z_{1}, \end{array}$$

(15) $$\begin{array}{cc} {\dot{z}}_{2} & =\beta z_{1}-\eta z_{2}z_{3}-\gamma z_{2}, \end{array}$$

(16) $$\begin{array}{cc} {\dot{z}}_{3} & =\alpha_{q}-\eta z_{3}\left(z_{1}+z_{2}\right)-\gamma z_{3}, \end{array}$$

where $z_{2}$ is the amplified output species, $\alpha_{q}$ defines the production rate of the quenching species, η is the sequestration rate, γ is the effective degradation or dilution rate and β denotes the downstream transcriptional gain.

It can be shown (Section S1) that at steady state, the system is characterized by an effective suppression factor $A^{*}$ that depends on the steady‐state value of $z_{3}$

(17) $$A^{*}:=\frac{\eta z_{3}^{*}+\gamma }{\gamma },$$

which quantifies the strength of sequestration‐mediated inhibition. The steady‐state concentrations of the activator species and output species can be expressed in terms of $A^{*}$

(18) $$z_{1}^{*}=\frac{u_{s}}{\gamma A^{*}},\;z_{2}^{*}=\frac{\beta u_{s}}{\gamma^{2}{\left(A^{*}\right)}^{2}}.$$

For the co‐expressed reporter, assuming negligible basal reporter production and the same removal rate γ, the steady‐state concentration is

$$r^{*}=\frac{\beta u_{s}}{\gamma^{2}A^{*}}=A^{*}z_{2}^{*}.$$

The circuit behavior is governed by the normalized input

(19) $$U=\frac{u_{s}}{\alpha_{q}},$$

which compares the effective activator input to the replenishment capacity of the quenching layer.

For sufficiently low U and sufficiently strong sequestration, production of the quenching species, $z_{3}$, dominates demand for its sequestration. In this regime, $z_{3}$ remains abundant, the effective suppression factor $A^{*}$ is large, and both activator species, $z_{1}^{*}$, and output species, $z_{2}^{*}$, are strongly attenuated. The circuit therefore operates in a *suppression regime*, in which weak‐input signals and basal leak are actively compressed.

For sufficiently large U, sequestration demand on the quenching species exceeds its replenishment. The quenching pool becomes progressively depleted, causing $A^{*}\to 1$ and releasing the activator from sequestration‐mediated inhibition. The output species $z_{2}$ then rises sharply through feedback‐assisted amplification. The circuit therefore transitions into an *amplification regime* characterized by strong output gain and enhanced discrimination between weak and strong inputs.

A useful initial tuning reference is

(20) $$u_{s}\approx \alpha_{q}.$$

As an initial design heuristic, the quenching strength can be chosen so that low effective inputs lie below this reference, while desired high‐input states exceed it:

(21) $$\ell +u\left(s^{\mathrm{low}}\right)<\alpha_{q}<\ell +u\left(s^{\mathrm{high}}\right)$$

Thus, the quenching layer selectively suppresses weak‐input states while preserving strong activation at larger inputs. For sufficiently large inputs, sequestration becomes negligible, and the output asymptotically approaches a linear amplifier

(22) $$z_{2}^{*}\approx \frac{\beta u_{s}}{\gamma^{2}}.$$

Compared to a direct single‐stage sensor, whose output scales as $u_{s}/\gamma$, the SensEnhance architecture therefore provides an additional amplification factor of β/γ when β>γ. Importantly, this amplification emerges selectively above threshold, whereas low‐input signals remain strongly suppressed.

Thus, SensEnhance reshapes the input‐output response by compressing weak‐input activity while selectively amplifying high‐input states. The activation threshold is directly tunable through $\alpha_{q}$, allowing the circuit operating range to be matched to the biological signal of interest (Figure 1e). Finally, it is interesting to note that the operation of the SensEnhance circuit is not unlike that of a threshold amplifier, whose output remains zero as long as the input falls below the threshold, but behaves as a standard amplifier above the threshold (Figure 1f).

### 2.3 Biological Implementation and Experimental Validation of SensEnhance

To experimentally implement SensEnhance (Figure 1d), we designed a circuit composed of three modular constructs (Figure 2a): an activator construct, a quencher construct, and a reporter construct. The activator construct places the transcription factor $z_{1}$ under the control of the sensing promoter $P_{\mathrm{Sensing}}$. The quencher construct expresses the sequestering species $z_{3}$ from a constitutive or inducible quencher promoter ($P_{\mathrm{Quencher}}$), depending on the desired level of control. The reporter construct contains the $z_{1}$‐responsive output promoter ($P_{\mathrm{Output}}$), which drives expression of the reporter r together with the additional competing species $z_{2}$. This organization separates sensing, quenching and output expression into exchangeable modules.

**FIGURE 2:**
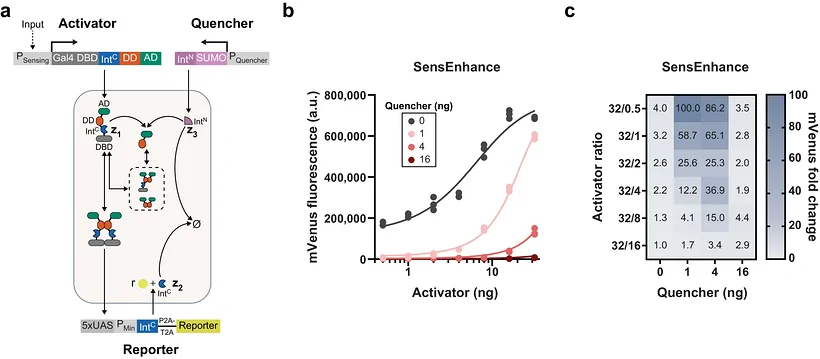
Biological implementation and validation of SensEnhance. **(a** **)** Schematic of the biological implementation of SensEnhance. The Gal4‐derived split‐intein transcription factor and its trans‐splicing‐mediated inactivation are based on the previously described platform [33]. The circuit comprises an activator, a quencher, and a reporter construct. The activator construct places a Gal4‐derived transcription factor (TF; $z_{1}$) under the control of the sensing promoter $P_{\mathrm{Sensing}}$. The TF carries the ${\mathrm{Int}}^{C}$ from the Gp41‐1 split intein between the Gal4 DNA‐binding domain (DBD) and the dimerization domain/activation domain (DD/AD). The quencher construct expresses the complementary SUMO‐tagged ${\mathrm{Int}}^{N}$ ($z_{3}$) from a constitutive or inducible quencher promoter $P_{\mathrm{Quencher}}$. Interaction between ${\mathrm{Int}}^{N}$ and the ${\mathrm{Int}}^{C}$‐containing TF triggers trans‐splicing, separating the DBD from the DD/AD and thereby preventing TF‐mediated activation of the output promoter. The reporter construct contains the TF‐responsive 5×UAS–MinP output promoter $P_{\mathrm{Output}}$, which drives expression of the fluorescent reporter r together with an additional ${\mathrm{Int}}^{C}$ species ($z_{2}$), separated by self‐cleaving P2A–T2A peptides. This output‐driven ${\mathrm{Int}}^{C}$ competes with the TF for available ${\mathrm{Int}}^{N}$, reducing further TF inactivation once the threshold set by ${\mathrm{Int}}^{N}$ expression has been exceeded. Additional spliced intermediates that are not expected to activate transcription are indicated by the dashed box. **(b, c** **)** Flow cytometry analysis of mVenus reporter fluorescence in HEK‐293T cells transfected with a fixed amount of reporter construct, increasing amounts of activator construct (0.5, 1, 2, 4, 8, 16, and 32ng), and the indicated amounts of quencher construct (0, 1, 4, and 16ng). Activator and quencher constructs were both driven by EF1α promoters. Fluorescence was analyzed 24h after transfection (**b**; *n* = 3). Fold changes in mVenus fluorescence between the indicated activator construct amounts shown in **b** are shown in **c**. Each dot represents the median fluorescence of one technical replicate, lines indicate fits of the SensEnhance model, and fold changes were calculated from the mean fluorescence of three replicates.

At the molecular level, we implemented the sequestration reactions using split intein‐mediated protein trans‐splicing. Split inteins are protein fragments, termed ${\mathrm{Int}}^{N}$ and ${\mathrm{Int}}^{C}$, that associate and catalyze protein trans‐splicing in a 1:1 stoichiometric ratio [32]. For our implementation, we adapted a Gal4‐based split‐intein system previously developed for intein‐mediated control [33, 34]. In this design, the transcription factor $z_{1}$ is Gal4‐derived and carries the ${\mathrm{Int}}^{C}$ from the Gp41‐1 split intein, inserted between the DNA‐binding domain and the dimerization/activation domains. The sequestering species $z_{3}$, driven by $P_{\mathrm{Quencher}}$, corresponds to the complementary ${\mathrm{Int}}^{N}$. Interaction between ${\mathrm{Int}}^{N}$ and the ${\mathrm{Int}}^{C}$‐containing transcription factor triggers trans‐splicing, separating the DNA‐binding domain from the dimerization and activation domains and thereby preventing transcription factor‐mediated activation of $P_{\mathrm{Output}}$. Intact Gal4‐derived transcription factor dimers activate the 5×UAS–MinP output promoter ($P_{\mathrm{Output}}$), which drives expression of the fluorescent reporter r together with an additional ${\mathrm{Int}}^{C}$ species corresponding to $z_{2}$. The reporter and ${\mathrm{Int}}^{C}$ are separated by self‐cleaving P2A–T2A peptides. This output‐driven ${\mathrm{Int}}^{C}$ competes with the transcription factor for available ${\mathrm{Int}}^{N}$, reducing further transcription factor inactivation and supporting high reporter expression once input‐dependent activator production exceeds the threshold imposed by ${\mathrm{Int}}^{N}$ abundance.

We selected a Gal4‐based architecture because activation of the output promoter requires both DNA binding and transcription factor dimerization. Therefore, spliced DNA‐binding fragments lacking the dimerization and activation domains are not expected to activate the promoter. Although additional spliced intermediates may form, including products that retain dimerization capacity (Figure 2a, dashed box), these species are not expected to contribute to transcriptional activation and were not considered explicitly in the model. Thus, only intact Gal4‐derived transcription factor dimers activate the output promoter.

We next validated the circuit in HEK‐293T cells. Cells were co‐transfected with a fixed amount of reporter plasmid and varying amounts of activator plasmid (0.5, 1, 2, 4, 8, 16, and 32ng) and quencher plasmid (0, 1, 4 and 16ng). The activator and quencher constructs were each driven by the EF1α constitutive promoter. In this configuration, increasing the amount of activator plasmid emulates increasing input‐dependent transcription factor production, whereas quencher plasmid titration sets the abundance of the threshold‐setting ${\mathrm{Int}}^{N}$.

In the absence of ${\mathrm{Int}}^{N}$ expression, reporter fluorescence remained high even at low activator levels, indicating substantial activation of the output promoter (Figure 2b, 0ng quencher construct). Co‐expression of a small amount of quencher construct strongly reduced reporter output in this low input regime, bringing fluorescence close to background while preserving high expression at elevated activator levels (Figure 2b, 1ng quencher construct). This increased the fold change between low and high activator inputs (0.5 and 32ng) from 4‐fold without quencher construct to 100‐fold with 1ng quencher construct (Figure 2c). In fold change heatmaps throughout the manuscript, rows indicate the input pairs being compared, columns indicate quencher conditions, and each cell gives the corresponding numerical fold change in reporter signal. Increasing the quencher construct amount further shifted the activation threshold toward higher activator levels, generating a family of input–output curves with distinct operating ranges. This tunability enabled SensEnhance to improve discrimination over selected input intervals. For instance, between intermediate and high activator levels (4 and 32ng), the fold change increased from 2.2‐fold without quencher construct to 37‐fold with 4ng quencher construct (Figure 2b,c). The SensEnhance model captured these quencher‐dependent shifts in the input–output profiles (Figure 2b, fitted curves). Because individual model parameters were not uniquely identifiable from steady‐state fluorescence measurements alone, the fits were used to describe response behavior rather than to infer unique biochemical parameter values; the fitting procedure and identifiability analysis are provided in Section S2.

To assess the contribution of output‐driven ${\mathrm{Int}}^{C}$ to relieving quenching and preserving strong reporter expression, we compared SensEnhance with the single sequestration circuit described in Figure 1c. Both circuits used the same constitutive promoters for activator and quencher expression and differed only in whether the reporter construct also encoded ${\mathrm{Int}}^{C}$. To compare the fraction of maximal output retained under quenching, we defined 100% for each circuit as the highest fluorescence measured without quencher over the tested activator range. With 1 ng of quencher plasmid, both circuits suppressed reporter expression at low input, but SensEnhance reached a peak normalized fluorescence of approximately 85%, compared with 50% for the single sequestration circuit (Figure S3a). SensEnhance also showed an approximately linear relationship between activator plasmid amount and fluorescence (Figure S3b), consistent with the signal linearization predicted in Section S1. Together, these results show that ${\mathrm{Int}}^{N}$‐mediated quenching suppresses reporter expression at low input, while output‐driven ${\mathrm{Int}}^{C}$ helps preserve strong output above a tunable activation threshold, thereby increasing the dynamic range over selected input intervals.

### 2.4 SensEnhance Converts a Mechanoresponsive Promoter Into a High‐Dynamic‐Range Matrix Stiffness Reporter

After experimentally validating that SensEnhance reduces low input reporter expression while preserving strong activation above a tunable threshold, we next asked whether this circuit architecture could be coupled to promoters regulated by endogenous mammalian pathways to obtain an enhanced readout. We focused on mechanoresponsive promoters because matrix mechanics encode physiologically and pathologically relevant information [28, 29, 30, 35], but endogenous mechanical inputs often generate modest transcriptional responses [36]. Among the mechanoresponsive promoters reported so far, a YAP/TAZ‐responsive promoter [31], an NF‐κB‐responsive promoter [37, 38], and the lamin A/C gene (*LMNA*) promoter [39] have been used to genetically report extracellular mechanics. Despite optimization efforts, for instance for YAP/TAZ‐responsive elements [31, 40, 41], these systems yielded only modest fold changes across conditions expected to strongly modulate mechanoresponsive promoter activity, including large differences in matrix stiffness (<1kPa vs. 40kPa) or sparse versus confluent cultures. As a result, genetic readouts of extracellular mechanics remain limited, particularly with sufficient precision to distinguish stiffness differences within a narrow mechanical range.

As possible inputs for the SensEnhance circuit, we evaluated the three promoters previously reported to respond to mechanical cues. To this end, NIH/3T3 fibroblasts were co‐transfected with firefly luciferase reporter constructs driven by each candidate promoter and a constitutively expressed Renilla luciferase reporter for normalization. As extracellular matrix substrates, we prepared 8‐arm polyethylene glycol‐vinyl sulfone (8‐arm PEG‐VS) hydrogels crosslinked with dithiothreitol (DTT) and functionalized with a cysteine‐containing RGD peptide to support cell adhesion (hereafter referred to as RGD‐DTT‐PEG hydrogels; Figure S4a). Hydrogel stiffness was tuned by varying the PEG‐VS concentration: 5.5% and 8% (w/v) PEG‐VS yielded soft and stiff hydrogel conditions with Young's moduli of 0.3 and 28kPa, respectively. Transfected cells were cultured on these matrices, and promoter activity was quantified by dual‐luciferase assay. Across the tested promoters, stiffness‐dependent fold changes were also modest in our system, ranging from only 1.2‐ to 1.4‐fold (Figure S5). Because the YAP/TAZ‐responsive promoter produced the largest stiffness‐dependent change (1.4‐fold), we selected it as input for SensEnhance. This promoter, 8xGTIIC, consists of eight GTIIC motifs recognized by TEAD transcription factors and is widely used to monitor YAP/TAZ transcriptional activity in response to substrate mechanics [5, 31, 42, 43, 44]. Its selection was further supported by the central role of YAP/TAZ in mechanotransduction. On soft matrices, limited integrin‐mediated force transmission and reduced cytoskeletal tension restrict YAP/TAZ nuclear accumulation, resulting in low TEAD‐dependent transcription. By contrast, stiffer matrices promote actomyosin contractility and force transmission to the nucleus, favoring YAP/TAZ nuclear localization and activation of TEAD‐dependent gene expression (Figure 3a) [45].

**FIGURE 3:**
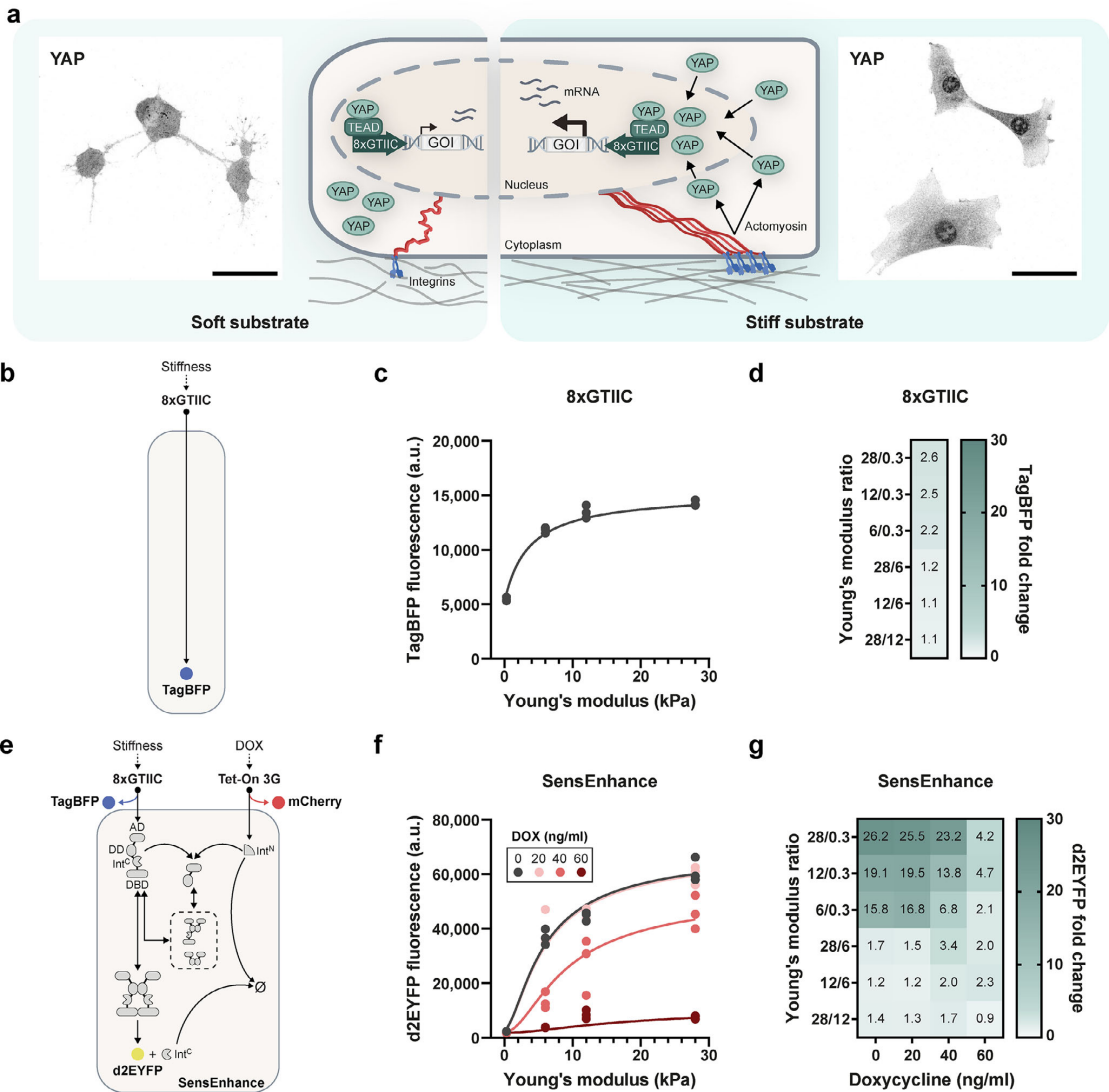
Implementation of SensEnhance with a YAP/TAZ mechanoresponsive promoter. **(a** **)** Schematic of stiffness‐dependent YAP/TAZ nucleocytoplasmic shuttling. On soft substrates, reduced integrin‐mediated force transmission and low cytoskeletal tension limit YAP/TAZ nuclear accumulation, resulting in low TEAD‐dependent transcription. Stiff substrates promote actomyosin contractility and force transmission to the nucleus, favoring YAP/TAZ nuclear localization and TEAD‐dependent gene expression. Insets show representative confocal microscopy images of YAP‐immunostained NIH/3T3 fibroblasts cultured on hydrogels with Young's moduli of 0.3kPa (soft, left) and 28kPa (stiff, right). Cells were fixed 24h after seeding. Scale bars are 50μm. **(b** **)** Schematic of the basic mechanoresponsive sensor, in which the YAP/TAZ‐responsive 8xGTIIC promoter drives TagBFP expression. **(c, d** **)** Flow cytometry analysis of TagBFP fluorescence driven by 8xGTIIC in cells cultured on RGD‐DTT‐PEG hydrogels with Young's moduli of 0.3, 6, 12 and 28kPa. Fluorescence was analyzed 64h after seeding (**c**; *n* = 3). Fold changes between the indicated Young's modulus conditions shown in **c** are shown in **d**. **(e** **)** Schematic of SensEnhance driven by 8xGTIIC. In this configuration, 8xGTIIC controls Gal4‐derived activator expression, whereas ${\mathrm{Int}}^{N}$ expression is controlled by a doxycycline‐inducible promoter (Tet‐On 3G). TagBFP reports 8xGTIIC activity, mCherry reports doxycycline‐induced ${\mathrm{Int}}^{N}$ expression, and d2EYFP reports SensEnhance output. **(f, g** **)** Flow cytometry analysis of d2EYFP fluorescence as SensEnhance output in cells cultured on RGD‐DTT‐PEG hydrogels with Young's moduli of 0.3, 6, 12 and 28kPa. Sixteen hours after seeding, ${\mathrm{Int}}^{N}$ expression was induced with 0, 20, 40 or $60\;\mathrm{ng}\;{\mathrm{mL}}^{-1}$ doxycycline (DOX), and fluorescence was analyzed 48h later (**f**; *n* = 3). Fold changes between the indicated Young's modulus conditions at each doxycycline concentration shown in **f** are shown in **g**. For **c** and **f**, each dot represents the median fluorescence of at least 2500 gated cells from one independently prepared hydrogel replicate, and lines indicate fits of the corresponding model. Fold changes in **d** and **g** were calculated from the mean fluorescence of three replicates.

Using SensEnhance, we generated a stable cell line capable of sensing and reporting matrix stiffness with a high dynamic range. To this end, we transduced NIH/3T3 fibroblasts with lentiviral particles encoding the 8xGTIIC‐driven SensEnhance circuit. To externally tune the activation threshold, the sequestering ${\mathrm{Int}}^{N}$ species was placed under the control of the Tet‐On 3G system [46], enabling doxycycline‐dependent induction of the quenching module. To monitor the activity of the circuit modules, we incorporated fluorescent markers into the corresponding expression cassettes. TagBFP was co‐expressed with the transcription factor from the 8xGTIIC promoter, separated by self‐cleaving peptides, enabling direct tracking of mechanosensing promoter activity. Similarly, mCherry was co‐expressed with ${\mathrm{Int}}^{N}$ from the Tet‐On 3G inducible promoter, allowing us to monitor doxycycline‐dependent quencher expression. Destabilized EYFP (d2EYFP, $t_{1/2}=2\;h$) was used as the output reporter of the full SensEnhance circuit (Figure 3e). From the transduced population, we generated monoclonal cell lines and screened them for doxycycline‐responsive circuit activity (Figure S6). Clone 1B4 was selected for subsequent experiments because it combined high fold change with typical NIH/3T3 growth and morphology (Figure S6c,d).

We first confirmed doxycycline‐dependent control of the quenching module (Figure S7). SensEnhance cells were seeded onto tissue‐culture plastic (Young's modulus $>10^{6}\;\mathrm{kPa}$), where YAP/TAZ‐driven circuit activation is maximal, and cultured with increasing concentrations of doxycycline (DOX). As anticipated, increasing doxycycline led to a dose‐dependent increase in mCherry fluorescence, confirming induction of the Tet‐On 3G‐controlled ${\mathrm{Int}}^{N}$. In parallel, d2EYFP fluorescence decreased as ${\mathrm{Int}}^{N}$ expression increased, consistent with enhanced trans‐splicing‐mediated inactivation of the transcription factor. This response identified a doxycycline range, approximately 0 to $60\;\mathrm{ng}\;{\mathrm{mL}}^{-1}$, in which circuit output could be gradually tuned without being completely suppressed (Figure S7). We therefore used this range for subsequent stiffness‐response experiments.

Next, we asked whether the engineered NIH/3T3 cells could sense and report differences in matrix stiffness. Cells were seeded on RGD‐DTT‐PEG hydrogels with Young's moduli of 0.3, 6, 12, and 28kPa, spanning a soft‐to‐stiff range that approximates mechanical environments from soft brain tissue through skin‐like matrices to stiffer pre‐calcified bone substrates (Figure S4b) [47]. Because TagBFP is co‐expressed from the 8xGTIIC input promoter, we first used TagBFP fluorescence to monitor the activity of the underlying mechanosensing promoter (Figure 3b). TagBFP fluorescence increased with substrate stiffness (Figure 3c). Across the full stiffness range tested, from 0.3 to 28kPa, the promoter produced a 2.6‐fold difference in fluorescence (Figure 3d). However, resolution within the intermediate stiffness range (6 to 28kPa) remained limited. Although most stiffness comparisons were statistically significant, the corresponding fold changes were modest; for example, fluorescence differed by only 1.2‐fold between 6 and 28kPa (Figure 3d). Thus, 8xGTIIC alone provided limited stiffness discrimination, highlighting a limitation for applications that require larger output differences between nearby stiffness conditions.

We next examined d2EYFP fluorescence as the output of the full SensEnhance circuit. In the absence of doxycycline, SensEnhance substantially increased stiffness‐dependent reporting, with the fold change between 0.3 and 28kPa rising from 2.6‐fold at the 8xGTIIC input‐promoter level to 26‐fold at the circuit output (Figure 3d,g). Notably, d2EYFP fluorescence on 0.3kPa hydrogels was already close to the lower output state of the circuit, despite detectable 8xGTIIC activity measured by TagBFP. Comparable low d2EYFP levels were observed on soft hydrogels after doxycycline treatment and on tissue‐culture plastic under high doxycycline conditions, where ${\mathrm{Int}}^{N}$ expression suppresses the circuit (Figure S8). Together, these observations suggest that, on soft matrices, the 8xGTIIC‐driven input remains too low to strongly engage the output promoter. This behavior is consistent with the nonlinear input–output relationship of SensEnhance, potentially combined with basal ${\mathrm{Int}}^{N}$ expression from the Tet‐On 3G system [48]. Indeed, without doxycycline, SensEnhance cells cultured on tissue‐culture plastic showed higher mCherry fluorescence than wild‐type cells, indicating leaky expression of the ${\mathrm{Int}}^{N}$‐containing quencher module. This leakiness was associated with reduced reporter output, as mCherry‐positive cells, identified using wild‐type cells as the negative reference, showed significantly lower d2EYFP fluorescence than mCherry‐negative cells (Figure S9). Such basal quenching could contribute to the low reporter output observed on soft hydrogels without doxycycline. Across the widest stiffness range tested, from soft hydrogels (0.3kPa) to very stiff tissue‐culture plastic ($>10^{6}\;\mathrm{kPa}$), the low circuit output observed on soft matrices contributed to a larger reporter dynamic range, increasing the difference between conditions from 3.2‐fold at the 8xGTIIC promoter level to 54.1‐fold at the full circuit output (Figure S10).

Despite this high dynamic range across the full stiffness range, the intermediate stiffness regime remained more difficult to resolve. Without doxycycline induction of the quenching arm, differences between intermediate and stiff conditions were mostly significant but still small, with fold changes ranging from 1.2‐ to 1.7‐fold (Figure 3g). We therefore asked whether doxycycline‐dependent ${\mathrm{Int}}^{N}$ production could sharpen SensEnhance discrimination in this regime. Consistent with the previous doxycycline dose‐response curve (Figure S7), $20\;\mathrm{ng}\;{\mathrm{mL}}^{-1}$ doxycycline had little effect on the circuit output (Figure 3f). In contrast, $40\;\mathrm{ng}\;{\mathrm{mL}}^{-1}$ doxycycline preferentially reduced d2EYFP fluorescence at low‐intermediate stiffnesses, particularly at 6kPa, while maintaining higher signal on 28kPa hydrogels (Figure 3f). Consequently, the fold change between 6 and 28kPa increased from 1.2‐fold for 8xGTIIC alone to 1.7‐fold without doxycycline and to 3.4‐fold with $40\;\mathrm{ng}\;{\mathrm{mL}}^{-1}$ doxycycline (Figure 3g). This improvement was also evident between 6 and 12kPa, where $40\;\mathrm{ng}\;{\mathrm{mL}}^{-1}$ doxycycline increased the fold change to 2‐fold and produced a statistically significant difference (p=0.0008), whereas SensEnhance cells without doxycycline showed only a non‐significant 1.2‐fold difference (p=0.09; two‐way ANOVA followed by Tukey's multiple‐comparisons test). At $60\;\mathrm{ng}\;{\mathrm{mL}}^{-1}$ doxycycline, d2EYFP fluorescence was strongly reduced across the stiffness range, indicating that excessive ${\mathrm{Int}}^{N}$ expression over‐suppresses the circuit. The resulting stiffness‐dependent output profiles across doxycycline concentrations were captured by fits of the SensEnhance model (Figure 3f).

Importantly, doxycycline treatment did not affect 8xGTIIC input‐promoter activity, as TagBFP fluorescence remained unchanged across doxycycline concentrations (Figures S11b, TagBFP histograms, and S12a). Similarly, mCherry fluorescence, used as a proxy for doxycycline‐induced ${\mathrm{Int}}^{N}$ expression, was comparable across stiffness conditions at each doxycycline concentration (Figures S11b, mCherry histograms, and S12b). The condition‐specific variation in mCherry fluorescence observed on hydrogels in the soft‐to‐intermediate stiffness regime may therefore reflect substrate‐dependent changes in cell state [31], including reduced proliferative dilution of fluorescent proteins or context‐dependent differences in Tet‐On induction [49]. Together, these results show that the engineered SensEnhance mechanoresponsive cell line combines substantially greater reporter contrast than 8xGTIIC alone with tunable stiffness discrimination. These cells already provide strong contrast between very soft and stiff hydrogels without doxycycline, while doxycycline‐induced quenching further sharpens discrimination over selected intermediate intervals. The level of quencher expression can therefore be adjusted to enhance contrast within the stiffness range of interest.

### 2.5 SensEnhance Sharpens Genetic Reporting of Enzymatically Modified and Spatially Patterned Matrix Stiffness

We next asked whether SensEnhance mechanosensing cells could improve endpoint discrimination following hydrogel stiffening or softening and across spatial stiffness patterns.

To induce changes in stiffness, we used an enzyme‐responsive PEG hydrogel system [50]. This system enables both hydrogel stiffening and softening through orthogonal enzymatic reactions. Stiffening is mediated by microbial transglutaminase (mTG), whereas softening is mediated by the *Clostridium difficile*‐derived protease PPEP‐1. The hydrogel formulation is based on RGD‐functionalized 8‐arm PEG‐VS crosslinked with two complementary bis‐cysteine peptides, Peptide‐Q and Peptide‐K. These peptides contain the glutamine (Q) and lysine (K) amino acids which are crosslinked upon addition of mTG, increasing the Young's modulus of the material (Figure 4a). The same peptide crosslinkers also contain the PPEP‐1 recognition sequence (PNP ↓ PVP) [51]. PPEP‐1 specifically hydrolyzes the proline–proline bond within this motif under oxidative conditions. Cleavage of the peptide crosslinks reduces network crosslinking density and thereby decreases the Young's modulus of the substrate (Figure 4e).

**FIGURE 4:**
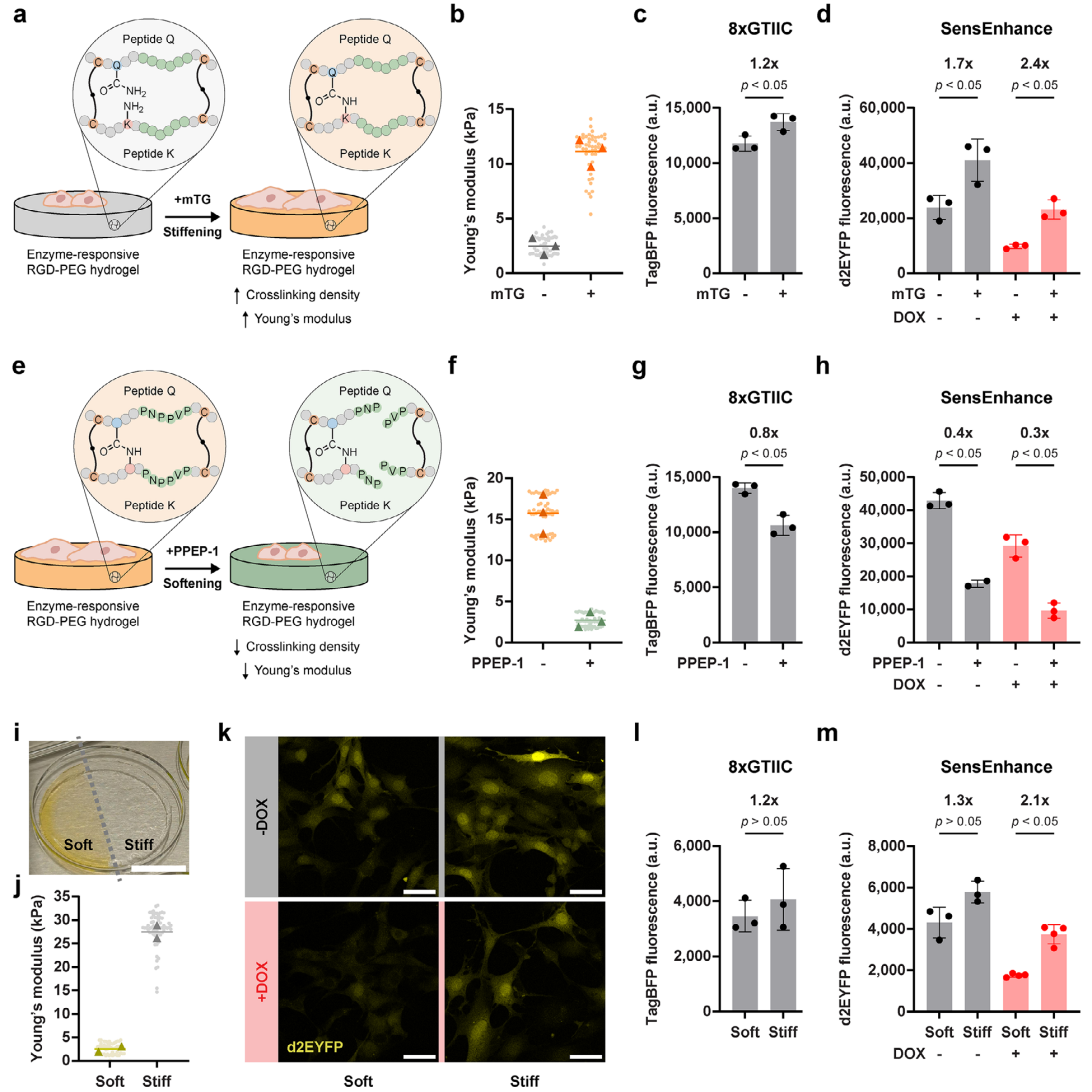
Endpoint reporting of hydrogel stiffening, softening and spatial stiffness patterns by SensEnhance mechanosensing cells. **(a** **)** Schematic of mTG‐mediated stiffening of RGD‐functionalized enzyme‐responsive PEG hydrogels. Peptide‐Q and Peptide‐K contain Q and K residues that enable mTG‐mediated secondary crosslinking. **(b** **)** Nanoindentation of non‐stiffened controls and hydrogels 24h after mTG treatment (*n* = 3). **(c, d** **)** Flow cytometry analysis of TagBFP fluorescence, reporting 8xGTIIC activity (**c**; *n* = 3), and d2EYFP fluorescence, reporting SensEnhance output with or without $40\;\mathrm{ng}\;{\mathrm{mL}}^{-1}$ doxycycline (**d**; *n* = 3), after mTG‐mediated stiffening. Cells were cultured on enzyme‐responsive hydrogels; 16h after seeding, mTG was added or omitted, and fluorescence was measured 24h later. **(e** **)** Schematic of PPEP‐1‐mediated softening. Peptide‐Q and Peptide‐K contain a PPEP‐1 recognition sequence (PNP↓PVP), whose cleavage reduces hydrogel crosslinking. **(f** **)** Nanoindentation of non‐softened controls and hydrogels 24h after PPEP‐1 treatment (*n* = 3). **(g, h** **)** Flow cytometry analysis of TagBFP fluorescence (**g**; *n* = 3) and d2EYFP fluorescence with or without $40\;\mathrm{ng}\;{\mathrm{mL}}^{-1}$ doxycycline (**h**; *n* = 3, except for the softened condition without doxycycline, for which *n* = 2 because one replicate was damaged during processing and excluded from analysis), after PPEP‐1‐mediated softening. Cells were cultured on mTG‐crosslinked hydrogels; 16h after seeding, PPEP‐1 was added or omitted, and fluorescence was measured 24h later. **(i** **)** Representative spatially patterned RGD‐DTT‐PEG hydrogel; fluorescent RGD marks the soft region, and the dotted line indicates the interface. Scale bar is 1cm. **(j** **)** Nanoindentation of soft and stiff regions in patterned hydrogels (*n* = 2). **(k** **)** Representative confocal images of the d2EYFP channel in cells cultured on soft or stiff regions of spatially patterned hydrogels, with or without $40\;\mathrm{ng}\;{\mathrm{mL}}^{-1}$ doxycycline, 48h after seeding. Scale bars are 50μm. **(l, m** **)** Image‐based quantification of TagBFP fluorescence (**l**; *n* = 3) and d2EYFP fluorescence (**m**; *n* = 3 for $0\;\mathrm{ng}\;{\mathrm{mL}}^{-1}$ doxycycline and *n* = 4 for $40\;\mathrm{ng}\;{\mathrm{mL}}^{-1}$ doxycycline) in soft and stiff regions. At least 500 cells were analyzed per condition. For nanoindentation plots, triangles indicate hydrogel means, dots indicate individual measurements, and horizontal lines indicate replicate means. For fluorescence plots, each dot represents the median fluorescence of cells from one independently prepared hydrogel replicate; bars represent the mean of replicate values and error bars indicate standard deviation. Fold changes are indicated. Statistical significance was assessed using unpaired two‐sided Welch's *t*‐tests.

For the induced stiffening experiment, we selected a hydrogel formulation with an initial Young's modulus of approximately 2.5kPa. Upon mTG‐mediated crosslinking ($700\;U\;L^{-1}$), the Young's modulus increased to approximately 11kPa (Figure 4b). Non‐stiffened hydrogels prepared from the same formulation served as controls representing the starting stiffness of the material. We chose this intermediate soft‐matrix regime as a challenging yet biologically relevant stiffness transition for further characterization of the engineered cells, as comparable changes in matrix mechanics occur during pathological tissue remodeling [52]. Twenty‐four hours after induction of stiffening, cells were analyzed by flow cytometry.

SensEnhance mechanosensing cells distinguished between the non‐stiffened control hydrogels and the mTG‐stiffened hydrogels. The 8xGTIIC input promoter, measured by TagBFP fluorescence, showed a 1.2‐fold difference between these two stiffness states (Figure 4c). The full SensEnhance circuit, measured by d2EYFP fluorescence, slightly increased this difference to 1.7‐fold in the absence of doxycycline (Figure 4d). Induction of the ${\mathrm{Int}}^{N}$ quenching arm with $40\;\mathrm{ng}\;{\mathrm{mL}}^{-1}$ doxycycline further increased the fold change to 2.4‐fold by reducing reporter fluorescence in the non‐stiffened control condition while maintaining higher output after stiffening (Figure 4d). Thus, doxycycline‐induced ${\mathrm{Int}}^{N}$ production enabled SensEnhance mechanosensing cells to distinguish mTG‐stiffened hydrogels from non‐stiffened controls within the intermediate stiffness regime with higher contrast than the 8xGTIIC promoter alone, effectively doubling the fold change readout.

We next tested whether the engineered cells could report the reverse mechanical transition: enzymatic softening of the hydrogel matrix (Figure 4e). For this, we started from mTG‐crosslinked hydrogels with a Young's modulus of approximately 16kPa. Upon PPEP‐1‐mediated cleavage of the peptide crosslinks ($0.048\;U\;L^{-1}$), the hydrogels softened to a Young's modulus of approximately 3kPa (Figure 4f). Non‐softened, mTG‐crosslinked hydrogels served as controls representing the starting stiffness before PPEP‐1‐mediated softening.

Flow cytometry analysis at 24h after induction of softening showed that the engineered cells distinguished PPEP‐1‐softened hydrogels from non‐softened controls. For the 8xGTIIC input promoter alone, TagBFP fluorescence showed a modest but statistically significant difference, with a softened‐to‐control fluorescence ratio of 0.8 (Figure 4g, p=0.031). By contrast, the full SensEnhance output showed a larger difference, with d2EYFP fluorescence decreasing to 0.4 of the control condition after softening in the absence of doxycycline (Figure 4h). Induction of ${\mathrm{Int}}^{N}$ expression with $40\;\mathrm{ng}\;{\mathrm{mL}}^{-1}$ doxycycline further reduced reporter fluorescence in the softened hydrogels, resulting in a softened‐to‐control fluorescence ratio of 0.3 (Figure 4h). SensEnhance therefore converted the modest 1.2‐fold 8xGTIIC response following softening into a 3.3‐fold high‐contrast biosensor output. Together with the stiffening results, these data demonstrate improved endpoint discrimination following enzyme‐mediated increases or decreases in hydrogel stiffness.

We additionally tested whether SensEnhance‐engineered cells could report spatial stiffness differences within the same sample. For this, we prepared RGD‐DTT‐PEG hydrogels containing two mechanically distinct regions (Figure 4i), with Young's moduli of approximately 2.5 and 27.5kPa (Figure 4j). This experiment moved the analysis from population‐level flow cytometry to spatially resolved fluorescence imaging. Direct microscopy‐based readout is particularly challenging for mechanosensing promoters because they typically report only modest fluorescence differences between soft and stiff substrates. Indeed, microscopy‐based detection of mechanosensitive reporters often requires additional signal enhancement, such as immunostaining [5] or incorporation of translational enhancer elements to increase reporter brightness [41], to resolve spatial differences in promoter activity. We therefore asked whether SensEnhance could provide sufficient fluorescent contrast to directly visualize local differences in matrix stiffness.

For the 8xGTIIC input promoter alone, comparison of TagBFP fluorescence between the soft and stiff regions yielded a non‐significant 1.2‐fold difference (p=0.46, Figure 4l and Figure S13). The SensEnhance output slightly increased this difference to 1.3‐fold in the absence of doxycycline, although the difference remained non‐significant (p=0.054, Figure 4k,m). In contrast, induction of the quenching arm with $40\;\mathrm{ng}\;{\mathrm{mL}}^{-1}$ doxycycline produced a clearer and statistically significant separation between stiffness regions (p=0.0027, Figure 4m). Under these conditions, d2EYFP fluorescence was reduced on the softer regions while remaining higher on the stiffer regions, increasing the fold change between both areas to 2.1‐fold (Figure 4k,m and Figure S13). These results show that SensEnhance provides sufficient fluorescence contrast for direct microscopy‐based detection of spatial differences in matrix stiffness that cannot be clearly distinguished using the previously described promoter alone.

### 2.6 SensEnhance Provides a Generalizable Strategy to Improve Sensing‐Promoter Performance

Having shown that SensEnhance improves stiffness‐dependent reporting from a mechanosensing promoter, we next asked whether this circuit architecture could be generalized to sensing promoters controlled by other endogenous pathways. We therefore applied SensEnhance to a STAT3‐responsive promoter composed of three sis‐inducible elements (3×SIE), a regulatory element commonly used to report STAT3 transcriptional activity [53]. This promoter reports IL‐6‐dependent inflammatory signaling: upon IL‐6 stimulation, receptor activation triggers JAK‐mediated STAT3 phosphorylation, followed by STAT3 dimerization, nuclear translocation and binding to SIE elements to induce transcription (Figure 5a). Hereafter, we refer to this promoter as 3xSTAT3‐RE.

**FIGURE 5:**
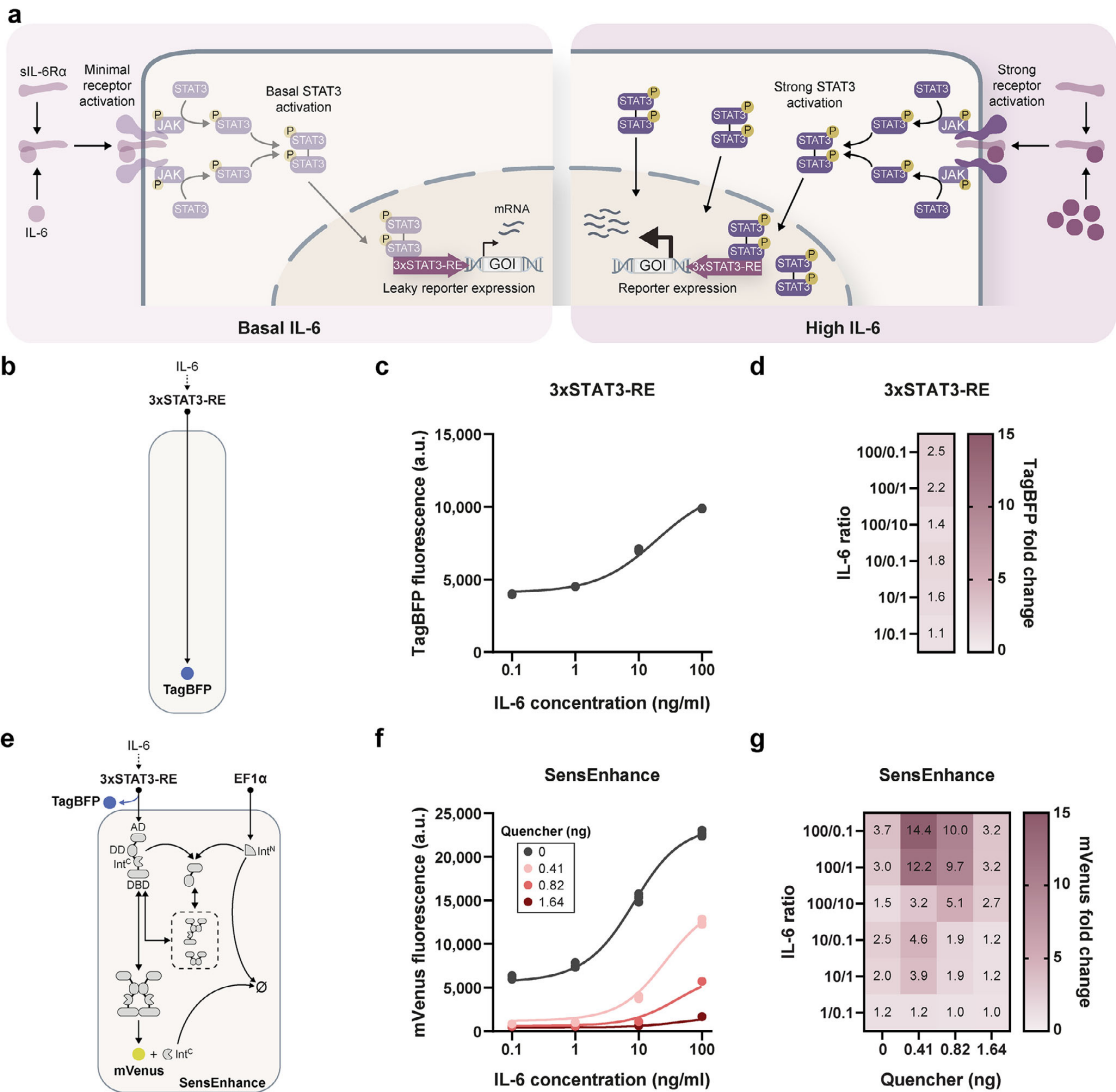
Generalization of SensEnhance to a STAT3‐responsive promoter. **(a** **)** Schematic of IL‐6/JAK/STAT3 trans‐signaling. In the presence of soluble IL‐6 receptor α (sIL‐6Rα), IL‐6 stimulation triggers JAK‐mediated STAT3 phosphorylation, STAT3 dimerization, nuclear translocation and binding to SIE elements to induce transcription. Basal pathway activity or stimulus‐independent promoter activity may contribute to reporter expression in the absence of exogenous IL‐6. **(b**) Schematic of the basic STAT3‐responsive sensor, in which the 3xSTAT3‐RE promoter, composed of three SIE elements, drives TagBFP expression. **(c, d** **)** Flow cytometry analysis of TagBFP fluorescence driven by the basic STAT3‐responsive sensor in transiently transfected HEK‐293T cells. Fluorescence was measured 24h after stimulation with IL‐6 (0.1, 1, 10 or $100\;\mathrm{ng}\;{\mathrm{mL}}^{-1}$) in the presence of $200\;\mathrm{ng}\;{\mathrm{mL}}^{-1}$ sIL‐6Rα (**c**; *n* = 4). Fold changes between the indicated IL‐6 concentrations shown in **c** are shown in **d**. **(e** **)** Schematic of SensEnhance driven by the 3xSTAT3‐RE promoter. In this configuration, 3xSTAT3‐RE controls Gal4‐derived activator expression, whereas ${\mathrm{Int}}^{N}$ expression was tuned by varying the quencher construct amount used for transfection. TagBFP reports 3xSTAT3‐RE activity, and mVenus reports SensEnhance output. **(f, g** **)** Flow cytometry analysis of mVenus fluorescence as SensEnhance output in transiently transfected HEK‐293T cells transfected with fixed amounts of activator and reporter constructs and increasing amounts of quencher construct (0, 0.41, 0.82 and 1.64ng). Fluorescence was measured 24h after stimulation with IL‐6 (0.1, 1, 10 or $100\;\mathrm{ng}\;{\mathrm{mL}}^{-1}$) in the presence of $200\;\mathrm{ng}\;{\mathrm{mL}}^{-1}$ sIL‐6Rα (**f**; *n* = 4). Fold changes between the indicated IL‐6 concentrations at each quencher construct amount shown in **f** are shown in **g**. For **c** and **f**, each dot represents the median fluorescence of one technical replicate, and lines indicate fits of the corresponding model. Fold changes in **d** and **g** were calculated from the mean fluorescence of four replicates.

To test this design, we transfected HEK‐293T cells with fixed amounts of the STAT3‐responsive input construct and the SensEnhance output construct, while titrating the EF1α‐driven quencher construct (0, 0.41, 0.82 and 1.64ng). Cells were additionally co‐transfected with a plasmid driving constitutive miRFP670 expression as a transfection control. As in the mechanosensing circuit, the sensing promoter co‐expressed the transcription factor and TagBFP, allowing us to monitor input activity independently (Figure 5b). Cells were stimulated with increasing concentrations of IL‐6 (0.1, 1, 10 or $100\;\mathrm{ng}\;{\mathrm{mL}}^{-1}$) together with a fixed concentration of $200\;\mathrm{ng}\;{\mathrm{mL}}^{-1}$ soluble IL‐6 receptor alpha (sIL‐6Rα), enabling IL‐6 trans‐signaling and STAT3‐dependent transcription.

The 3xSTAT3‐RE input promoter responded to IL‐6 stimulation, but its dynamic range was limited (Figure 5c). Across the full concentration range tested, from 0.1 to $100\;\mathrm{ng}\;{\mathrm{mL}}^{-1}$ IL‐6, TagBFP fluorescence increased by 2.5‐fold (Figure 5d). However, discrimination over narrower input intervals remained modest, with 1.6‐and 1.4‐fold differences between 1 and $10\;\mathrm{ng}\;{\mathrm{mL}}^{-1}$ and between 10 and $100\;\mathrm{ng}\;{\mathrm{mL}}^{-1}$ IL‐6, respectively.

We next evaluated mVenus fluorescence as the output of the full 3xSTAT3‐RE‐driven SensEnhance circuit (Figure 5e), whose IL‐6‐dependent response profiles were captured by fits of the corresponding SensEnhance model (Figure 5f). In the absence of the quencher construct, SensEnhance only modestly increased the difference between IL‐6 conditions (Figure 5f), reaching a 3.7‐fold change between 0.1 and $100\;\mathrm{ng}\;{\mathrm{mL}}^{-1}$ IL‐6 (Figure 5g). This limited improvement was linked to substantial mVenus expression at low IL‐6 concentrations (Figure 5f), indicating that basal activity from the input promoter was propagated through the downstream circuit. Co‐transfection with quencher construct suppressed this low‐input output (Figure 5f), bringing fluorescence close to background while preserving higher expression under strong IL‐6 stimulation. Consequently, the fold change between 0.1 and $100\;\mathrm{ng}\;{\mathrm{mL}}^{-1}$ IL‐6 increased to 14.4‐fold with 0.41ng quencher construct (Figure 5g). Representative histograms illustrating this increase in fold change with co‐transfection of the quencher construct are shown in Figure S14b (mVenus panels). Increasing the amount of quencher construct shifted the operating range of the circuit, allowing SensEnhance to improve discrimination over selected IL‐6 intervals (Figure 5f). For example, between 10 and $100\;\mathrm{ng}\;{\mathrm{mL}}^{-1}$ IL‐6, SensEnhance produced a 5.1‐fold difference with 0.82ng quencher construct, compared with 1.4‐fold for the 3xSTAT3‐RE promoter alone and 1.5‐fold for SensEnhance without quencher construct (Figure 5d,g).

Because transient transfection can introduce copy‐number‐dependent resource‐burden effects (Figure S15a,b), we also normalized reporter fluorescence to constitutively expressed miRFP670. After this correction, SensEnhance still improved IL‐6‐dependent reporter discrimination (Figure S15g,h), suggesting that this improvement was not explained solely by transfection‐associated variation or resource burden. Time‐course measurements following an acute increase to $100\;\mathrm{ng}\;{\mathrm{mL}}^{-1}$ IL‐6 also showed improved reporting with 0.41ng quencher construct compared with the 3xSTAT3‐RE promoter alone or SensEnhance without quencher (Figure S16).

Although quencher co‐transfection improved fold change, it also reduced reporter output at high IL‐6 concentrations. To test whether strengthening output‐mediated feedback could compensate for this reduction, we compared reporter constructs encoding mVenus alone ($0\times {\mathrm{Int}}^{C}$) or together with one or two ${\mathrm{Int}}^{C}$ copies separated by self‐cleaving peptides. HEK‐293T cells were transfected with each circuit variant using fixed amounts of sensor and reporter constructs and varying amounts of quencher construct (0, 0.41 and 0.69ng), and stimulated with 0.1 or $100\;\mathrm{ng}\;{\mathrm{mL}}^{-1}$ IL‐6. At high IL‐6 input, reporter fluorescence decreased as the number of output‐encoded ${\mathrm{Int}}^{C}$ copies increased, even without quencher (Figure S17a), potentially reflecting the additional expression burden of longer constructs or incomplete separation at the self‐cleaving peptide sites.

In the presence of quencher, including at least one output‐encoded ${\mathrm{Int}}^{C}$ copy substantially improved relief of quenching compared with the construct lacking ${\mathrm{Int}}^{C}$, consistent with the constitutive‐promoter experiments (Figure S3). At $100\;\mathrm{ng}\;{\mathrm{mL}}^{-1}$ IL‐6 and 0.69ng quencher construct, the $1\times {\mathrm{Int}}^{C}$ and $2\times {\mathrm{Int}}^{C}$ variants retained approximately 65% and 74% of their respective no‐quencher output, compared with approximately 35% for the $0\times {\mathrm{Int}}^{C}$ variant (Figure S17b). The modest increase in the fraction of output retained with a second copy supports the hypothesis that output‐produced ${\mathrm{Int}}^{C}$ is not the main factor limiting reporter expression under these conditions, potentially because it is already expressed downstream of a transcriptional amplification step. Approaching maximal reporter expression may instead require higher activator levels than the sensing promoter can provide under biologically relevant stimulation. Overall, the standard $1\times {\mathrm{Int}}^{C}$ SensEnhance reporter combined high signal with relief of quenching and achieved the highest fold change between 0.1 and $100\;\mathrm{ng}\;{\mathrm{mL}}^{-1}$ IL‐6 among the variants tested (Figure S17c).

To test whether SensEnhance‐mediated sensing enhancement extends beyond fluorescent reporters, we replaced mVenus in the IL‐6‐responsive circuit with human IL‐10 as a representative secreted output protein. Cells were transfected with fixed amounts of sensor and effector constructs and varying amounts of quencher construct (0, 0.41, or 0.82ng). At 24h after transfection, cells were stimulated with 0.1 or $100\;\mathrm{ng}\;{\mathrm{mL}}^{-1}$ IL‐6, and supernatant IL‐10 concentrations were quantified by ELISA 24h later. Quencher co‐transfection reduced IL‐10 concentrations at low IL‐6 input from approximately 4 to $\leq 1\;\mathrm{ng}\;{\mathrm{mL}}^{-1}$, while maintaining concentrations of 17–19 $\mathrm{ng}\;{\mathrm{mL}}^{-1}$ at high input. The fold difference in IL‐10 concentration between low and high IL‐6 input therefore increased from 6.8‐fold without quencher to 18.7‐fold with 0.82ng quencher construct (Figure S18). Together, these results support SensEnhance as a generalizable strategy to improve sensing‐promoter performance across distinct biological contexts, with outputs ranging from fluorescent reporters to secreted proteins.

## 3 Discussion

In this work, we developed SensEnhance, a modular genetic circuit that improves the performance of mammalian sensing promoters. Promoters responsive to endogenous signals are attractive inputs for synthetic gene circuits because they link physiologically relevant cellular states to genetic programs. However, many such promoters exhibit modest induction, high basal activity, and limited tunability, reducing the contrast between biologically relevant input states.

Building on the previously established Gal4‐based split‐intein platform [33], SensEnhance combines adjustable transcription‐factor quenching with positive feedback through output‐driven relief of quenching to improve sensing‐promoter performance. Unlike input‐layer engineering approaches [17, 19], this architecture leaves the sensing promoter unchanged and requires no manipulation of upstream signaling components. Transcriptional cascades and positive feedback strategies can amplify sensor output [20, 22, 54]. SensEnhance couples output amplification to adjustable quenching, enabling suppression of basal output and tuning of the sensor's operating range. This quenching acts directly on the synthetic transcription factor, reducing the pool of functional activator available for output expression. It therefore differs from promoter‐level thresholding based on competitive DNA binding [21] and output‐layer regulation of the reporter [10]. In parallel, output‐driven expression of additional ${\mathrm{Int}}^{C}$ helps mitigate the reduction in maximal expression that can accompany this quenching, addressing a common trade‐off in high‐performance biosensor circuits [23]. Related intein‐based controllers couple TNF‐α sensing to adalimumab secretion for closed‐loop regulation of extracellular TNF‐α [34]. In SensEnhance, output‐driven expression of free ${\mathrm{Int}}^{C}$ instead provides intracellular positive feedback to enhance sensing‐promoter readouts. The IL‐6/IL‐10 experiments assess how an externally supplied IL‐6 input drives IL‐10 secretion; feedback regulation of extracellular IL‐6 is not evaluated.

To illustrate the potential of SensEnhance, we coupled it to 8xGTIIC, a YAP/TAZ‐responsive promoter widely used to monitor mechanotransduction. Tissue mechanics are increasingly recognized as informative features of physiological and pathological states, motivating efforts to engineer cells that sense, report, or respond to altered mechanical environments [55, 56, 57, 58]. However, modest transcriptional outputs often limit the use of mechanoresponsive promoters as inputs to synthetic genetic programs. SensEnhance increased 8xGTIIC‐driven stiffness reporting beyond the promoter alone, enabling more robust detection of static, induced and spatial differences in matrix stiffness. This principle can be extended beyond mechanotransduction to other biologically informative but transcriptionally weak endogenous signals, as shown here for the IL‐6/STAT3 inflammatory pathway and potentially for metabolic [13, 59] or stress‐associated pathways [10, 60].

Several design constraints remain. The achievable output contrast depends on the strength and information content of the upstream promoter. Similarly, although output‐coupled feedback supports high‐gain amplification, we observed reduced reporter expression at high input compared with the corresponding circuit without quencher. The SensEnhance model predicts that maximal expression is approached asymptotically as activator input increases. However, weak sensing promoters, such as the IL‐6‐responsive and mechanoresponsive promoters, may not drive sufficient activator expression to reach this regime under biologically relevant conditions. Approaching the predicted maximum could therefore require unrealistically high stimulation, potentially at levels where the circuit without quencher has already saturated. Additionally, quencher expression should be tuned to each promoter's activity and the input range of interest. Insufficient quenching may leave basal output or responses below a selected threshold largely unchanged, whereas excessive quenching can suppress the desired induced response.

The choice of quencher‐control system also matters. For stable circuit implementation, the Tet‐On 3G system provides a practical way to tune ${\mathrm{Int}}^{N}$ expression, but doxycycline‐inducible systems can exhibit basal leakiness [48], dose sensitivity, and heterogeneous induction across cells [61]. Future implementations could replace this module with alternative inducible systems that provide more precise control [48, 62]. Finally, lentiviral delivery enables stable integration for long‐term applications but may require clone screening to balance expression of the sensing, quenching, and output modules. Integration‐site effects, copy‐number variation, and selective silencing may alter performance over time. Defined genomic landing pads, insulated expression cassettes, or more compact vectors may improve robustness.

Looking forward, SensEnhance could broaden the use of mammalian sensing promoters as inputs to synthetic gene circuits. Its modular architecture supports both fluorescent reporters and secreted protein outputs, while adjustable quencher expression enables the response range to be tuned to the input levels of interest. These features may support the development of tunable genetic biosensors for monitoring cellular signaling and tissue microenvironments.

## 4 Experimental Section

### 4.1 Construction of Expression Plasmids

All plasmids generated in this study were assembled by Gibson Assembly. The plasmid list is provided in Table S2, and the DNA sequences of the plasmids generated in this study are provided in Section S5 (Table S9). *E. coli* transformed with lentiviral vectors or constructs containing multiple repeats, such as response elements in the promoter region, were grown at $30^{\circ }C$. All plasmids were verified by Sanger sequencing. The plasmid 8xGTIIC‐luciferase was a gift from Stefano Piccolo (Addgene plasmid #34615) [31]. The plasmid pLVX‐TetOne‐Puro‐GFP was a gift from Jason Sheltzer (Addgene plasmid #171123) [63]. Plasmids pLTR‐G and pCD/NL‐BH*DDD were a gift from Jakob Reiser (Addgene plasmids #17532 and #17531, respectively) [64]. *LMNA* promoter was a kind gift from Dennis Discher (University of Pennsylvania) [39].

### 4.2 Mammalian Cell Culture and Transfection

Human embryonic kidney cells (HEK‐293T, DSMZ no. ACC 635), mouse embryonic fibroblasts (NIH/3T3, DSMZ no. ACC 59) and stable cell lines generated in this study were cultured in Dulbecco's modified Eagle's medium (DMEM, PAN Biotech, cat. no. P04‐03550) supplemented with 10% (v/v) fetal calf serum (FCS, PAN Biotech, cat. no. P30‐3602) and 1% (v/v) penicillin‐streptomycin solution (P/S, PAN Biotech, cat. no. P06‐07100) (referred to as complete medium) at 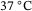 with 5% ${\mathrm{CO}}_{2}$. Cells were passaged every 2–3 days upon reaching 80% confluency.

For transient transfection experiments, 10,000 HEK‐293T cells were seeded per well of a 96‐well plate in 100μL of complete medium. After 24h, cells were transfected using polyethyleneimine (PEI, stock solution: $1\;\mathrm{mg}\;{\mathrm{mL}}^{-1}$ in $H_{2}O$, pH 7, Polysciences, cat. no. 23966‐1) as described before [65]. For each well, DNA/PEI complexes were formed by mixing 100ng of plasmid DNA and 0.33μL of PEI stock solution in 20μL of Opti‐MEM (Thermo Fisher, cat. no. 22600‐134). After a 20‐min incubation at room temperature, the DNA/PEI complexes were added dropwise to each well. Plasmid amounts and combinations for each experiment are described in Tables S3–S7.

### 4.3 Generation of Monoclonal Cell Lines

NIH/3T3 fibroblasts were stably transduced using VSV‐G‐pseudotyped lentiviral particles as described before [66]. To produce lentiviral particles, 250 000 HEK‐293T cells were seeded per well of a 6‐well plate in 2mL of complete medium. After 24h, the cells were transfected using PEI. The envelope plasmid pLTR‐G, the packaging plasmid pCD/NL‐BH*DDD, and the vector plasmid encoding the gene of interest were mixed in a mass ratio of 1:1:2 (total mass: 4000ng per well) together with 13.3μL of the PEI stock solution in 267μL of Opti‐MEM. After a 20‐min incubation at room temperature, the DNA/PEI complexes were added to the cells dropwise. Six to eight hours after transfection, the medium of the cells was carefully changed to Advanced DMEM (Thermo Fisher, cat. no. 12491015) containing 2% FCS, 10 µm cholesterol (Sigma‐Aldrich, cat. no. C4951), 10 µm egg lecithin (Serva Electrophoresis, cat. no. 27608), and 1× chemically defined lipid concentrate (Thermo Fisher, cat. no. 11905031). The medium containing the viral particles was harvested after 48h and filtered through a 0.45μm CA filter (Whatman Puradisc, cat. no. 10462100).

For transduction, 8000 NIH/3T3 fibroblasts were seeded per well in a 24‐well plate with 0.5mL of complete medium. After 24h, the medium was replaced with complete medium containing $8\;\mu g\;{\mathrm{mL}}^{-1}$ polybrene (Sigma‐Aldrich, cat. no. TR‐1003), and freshly produced, filtered lentiviral particles were added. Specifically, for the generation of SensEnhance mechanoresponsive cells, 10, 75, and 100μL of lentiviral supernatant corresponding to the reporter (pMB025k), activator (pMB042), and quencher (pMB029) constructs, respectively, were added per well. These volumes were selected based on titration experiments in unsorted cells to achieve low reporter expression in the absence of the activator, sufficient expression in its presence, and doxycycline‐dependent suppression upon induction of the quencher (Figure S6a).

Monoclonal cell lines were generated by sorting single d2EYFP (pMB025k) and TagBFP (pMB042) double‐positive cells into 96‐well plates using an SH800S Cell Sorter (Sony Biotechnology). Following expansion, clones were screened for (1) reporter fluorescence intensity, (2) doxycycline responsiveness, and (3) typical NIH/3T3 morphology and growth. The best‐performing clone was selected for further studies.

### 4.4 Hydrogel Preparation

8‐arm 10kDa polyethylene glycol‐vinyl sulfone (PEG‐VS) was purchased from JenKem Technology. The cysteine‐containing RGD peptide Ac‐GCGYGRGDSPG‐${\mathrm{NH}}_{2}$, its fluorescent variant (5,6‐FAM)‐GCGYGRGDSPG‐${\mathrm{NH}}_{2}$, and the enzyme‐responsive crosslinking peptides Ac‐GCRDWALQRPYPNPPVPDRCG‐${\mathrm{NH}}_{2}$ (Peptide‐Q) and Ac‐GCRDGAGKYVAPNPPVPDRCG‐${\mathrm{NH}}_{2}$ (Peptide‐K) were synthesized by Caslo ApS (Technical University of Denmark). Dithiothreitol (DTT; PanReac AppliChem, cat. no. A1101) was used as a crosslinker for the RGD‐DTT‐PEG hydrogels. All precursor materials were dissolved in 0.3 m triethanolamine (TEA, pH 8; Carl Roth, cat. no. 6300.1) before use.

Hydrogels were formed by Michael‐type addition, as described previously [50]. Briefly, PEG‐VS stock solution (25% (w/v)) was diluted in 0.3 m TEA and reacted with the RGD peptide for 5min at room temperature. The corresponding crosslinker was then added, and 40μL of the precursor solution were immediately cast between a Sigmacote‐treated hydrophobic glass slide (Sigma‐Aldrich, cat. no. SL2) and a 20mm diameter glass coverslip functionalized with 3‐aminopropyltriethoxysilane (APTES; Sigma‐Aldrich, cat. no. 440140). Coverslips were functionalized as previously described [67]. Hydrogels were allowed to polymerize for 1h at room temperature and subsequently washed at least three times with PBS for ≥5min.

For RGD‐DTT‐PEG hydrogels, the final RGD concentration was 2.5 mm, and DTT was added at a 1.2‐fold molar excess of thiol to vinyl sulfone groups. Hydrogel stiffness was tuned by varying the final PEG‐VS concentration (5.5%, 6%, 6.5%, and 8% (w/v)), while keeping the RGD concentration constant across all conditions. Hydrogels with spatial differences in stiffness were prepared using 5.75% and 8% PEG‐VS precursor solutions. For this, 20μL of each precursor solution was placed 1cm apart on a Sigmacote‐treated glass slide, followed by placement of an APTES‐functionalized coverslip on top. For representative visualization of spatial patterning, selected hydrogels were prepared with fluorescently labeled RGD in the soft region and imaged to confirm region‐specific hydrogel formulation.

Enzyme‐responsive hydrogels were prepared using the same casting procedure, with final PEG‐VS concentrations of 2.5% (w/v) for stiffening gels and 2.75% (w/v) for softening gels. The final RGD concentration was 1 mm. Peptide‐K and Peptide‐Q were used as crosslinkers at a 1.25‐fold molar excess of thiol to vinyl sulfone groups.

Table S8 provides a detailed overview of stock concentrations and hydrogel compositions.

### 4.5 Mechanical Characterization of Hydrogels

Hydrogels were mechanically characterized by nanoindentation using a Pavone Nanoindenter (Optics11) equipped with a spherical probe (10.5μm radius) attached to a cantilever with a spring constant of $0.49\;N\;m^{-1}$. Indentation measurements were performed at room temperature in PBS at a speed of $1\;\mu m\;s^{-1}$, and each hydrogel was indented 20 times at different positions 150μm apart. At least two different hydrogels were measured for each polymer concentration. Young's moduli were derived by fitting the resulting force‐indentation curves up to a 1μm indentation depth using the Hertz model for a spherical indenter:

(23) $$F=\frac{4}{3}\frac{E}{1-\nu^{2}}R^{1/2}h_{c}^{3/2}$$

where E is the Young's modulus of the hydrogel, ν is the Poisson ratio, R is the radius of the spherical probe, and $h_{c}$ is the indentation depth. A Poisson ratio of 0.5 was used for the PEG hydrogels [68].

### 4.6 Dual Luciferase Assay

NIH/3T3 fibroblasts were seeded in 10‐cm dishes at 295 000 cells per dish in 10mL complete medium 24h before transfection. Cells were transfected with 22.5μg total DNA and 75μL PEI in 1.5mL Opti‐MEM. Detailed transfection mixes are listed in Table S3. After 24h, cells were detached and seeded onto RGD‐DTT‐PEG hydrogels containing 5.5% or 8% PEG at a density of approximately 7700 $\mathrm{cells}\;{\mathrm{cm}}^{-2}$. Cells were lysed 48h after seeding by adding 250μL lysis buffer per well (25 mm Tris‐HCl, pH 7.8, 1% (v/v) Triton X‐100, 15 mm ${\mathrm{MgSO}}_{4}$, 4 mm EGTA and 1 mm DTT). After 5min incubation at room temperature, lysates were resuspended and transferred as two 80μL aliquots in white flat‐bottom 96‐well plates (Corning, CORN3912). Plates were centrifuged for 1min at 1,000×g to remove bubbles. Firefly luciferase activity was measured using a Spark microplate reader (Tecan) by adding 20μL firefly substrate (0.47 mm luciferin, 20 mm Tricine, 2.67 mm ${\mathrm{MgSO}}_{4}$, 0.1 mm EDTA, 33.3 mm DTT, 0.52 mm ATP, 0.27 mm acetyl‐CoA, 5 mm NaOH and 0.264 mm ${\mathrm{MgCO}}_{3}$) to one plate. Renilla luciferase activity was measured in the second plate by adding 20μL coelenterazine solution per well (47.2 µm coelenterazine in PBS). Luminescence was measured immediately after substrate addition. Firefly luciferase values were normalized to background‐subtracted Renilla luciferase values.

### 4.7 SensEnhance Mechanoresponsive Cell Seeding and Treatment

SensEnhance mechanoresponsive cells were seeded at approximately 7700 $\mathrm{cells}\;{\mathrm{cm}}^{-2}$ in 12‐well plates. Depending on the experiment, cells were seeded either on tissue‐culture plastic or on hydrogels and treated as described below.

For doxycycline titration (Figure S7), cells were seeded onto tissue‐culture plastic. Sixteen hours after seeding, the medium was replaced with 1mL complete medium containing 0, 20, 40, 60, 80, 100, or $250\;\mathrm{ng}\;{\mathrm{mL}}^{-1}$ doxycycline (DOX, Doxycycline hyclate, Sigma‐Aldrich, catalog no. D5207).

For stiffness‐dependent measurements (Figure 3c,f), cells were seeded on RGD‐DTT‐PEG hydrogels containing 5.5%, 6%, 6.5%, or 8% PEG, or on tissue‐culture plastic as a control. Sixteen hours after seeding, the medium was replaced with 1mL complete medium containing 0, 20, 40, or $60\;\mathrm{ng}\;{\mathrm{mL}}^{-1}$ doxycycline.

For induced stiffening experiments (Figure 4c,d), cells were seeded on enzyme‐responsive hydrogels. Sixteen hours after seeding, doxycycline treatment and hydrogel stiffening were initiated simultaneously. The medium was replaced with complete medium containing 0 or $40\;\mathrm{ng}\;{\mathrm{mL}}^{-1}$ doxycycline, and 50μL microbial transglutaminase solution in PBS was added to the corresponding samples to induce hydrogel stiffening ($700\;U\;L^{-1}$; mTG; Transglutaminase TI, $140\;U\;g^{-1}$, Bindly). Control samples received 50μL PBS without enzyme.

For induced softening experiments (Figure 4g,h), cells were seeded on previously mTG‐crosslinked enzyme‐responsive hydrogels. Sixteen hours after seeding, doxycycline treatment and hydrogel softening were initiated simultaneously. The medium was replaced with complete medium containing 0 or $40\;\mathrm{ng}\;{\mathrm{mL}}^{-1}$ doxycycline, and 50μL purified PPEP‐1 protease solution in PBS was added to the corresponding samples to induce hydrogel softening ($0.048\;U\;L^{-1}$). Control samples received 50μL PBS without enzyme. PPEP‐1 was expressed, purified and characterized as described previously [50].

For flow cytometry experiments, cells were collected 24h (for induced stiffening or softening experiments) or 48h after treatment. Samples were washed with PBS and detached using trypsin. For hydrogel samples, hydrogels were first transferred to a new plate before washing and cell detachment. Trypsin was inactivated with complete medium, and cells were pelleted, resuspended in FACS buffer (PBS containing 2% (v/v) FCS), and analyzed directly by flow cytometry.

For spatial stiffness experiments (Figure 4k–m), cells were seeded on hydrogels with spatially distinct stiffness regions at a density of approximately 7700 $\mathrm{cells}\;{\mathrm{cm}}^{-2}$ in complete medium containing 0 or $40\;\mathrm{ng}\;{\mathrm{mL}}^{-1}$ doxycycline. After 48h, cells were fixed with 4% (w/v) paraformaldehyde for 30min, washed with PBS, and imaged directly using a confocal microscope.

### 4.8 IL‐6 Stimulation

Twenty‐four hours after transfection with the IL‐6‐responsive SensEnhance circuit, cells were stimulated with 0.1, 1, 10, or $100\;\mathrm{ng}\;{\mathrm{mL}}^{-1}$ IL‐6 (PeproTech, cat. no. 200‐06) in the presence of $200\;\mathrm{ng}\;{\mathrm{mL}}^{-1}$ soluble IL‐6 receptor alpha (sIL‐6Rα) (PeproTech, cat. no. 200‐06RC) to enable IL‐6 trans‐signaling. The final volume was 140μL per well. Cells were analyzed after 24h of stimulation, except in the time‐course experiment, which included measurements at 0, 4, 8, 12, 24, and 48h. At each time point, cells were detached using trypsin, resuspended in FACS buffer, and analyzed directly by flow cytometry. For IL‐10 quantification, culture medium was collected before cell detachment and centrifuged at 2000×g for 10min, and the resulting supernatant was analyzed by ELISA.

### 4.9 IL‐10 Quantification

IL‐10 in the cleared cell supernatant was quantified with a Human IL‐10 ELISA Kit (ab185986, Abcam) according to the manual provided by the manufacturer. Optical density was measured at 450nm on a Spark microplate reader (Tecan). IL‐10 concentrations were interpolated from a blank‐corrected standard curve using an unweighted four‐parameter logistic fit and corrected for sample dilution.

### 4.10 Flow Cytometry

Cells were analyzed using an Attune NxT flow cytometer (Thermo Fisher Scientific). TagBFP fluorescence was detected using the VL1 channel with excitation at 405nm and emission collected through a 440/50nm filter. mVenus and d2EYFP fluorescence were detected using the BL1 channel with excitation at 488nm and emission collected through a 530/30nm filter. mCherry fluorescence was detected using the YL2 channel with excitation at 561nm and emission collected through a 620/15nm filter. miRFP670 fluorescence was detected using the RL1 channel with excitation at 637nm and emission collected through a 670/14nm filter. Data were analyzed using FlowJo v10.0. For the mechanosensing monoclonal cell line, live single cells were included in the analysis (gating strategy shown in Figure S11a). For transient transfection experiments, live single cells were further gated for miRFP670 and mVenus double‐positive populations (gating strategy shown in Figure S14a). At least 2500 gated cells were included in the analysis for each replicate. Median fluorescence intensity was used for comparisons between conditions.

### 4.11 YAP Immunostaining

NIH/3T3 wild type cells were seeded on 5.5% and 8% RGD‐DTT‐PEG hydrogels at a density of 7700 $\mathrm{cells}\;{\mathrm{cm}}^{-2}$. After 24h, cells on hydrogels were fixed with 4% paraformaldehyde for 30min at room temperature. Samples were then washed with PBS and incubated with 50 mm ${\mathrm{NH}}_{4}\mathrm{Cl}$ in PBS for 1h at room temperature to quench free aldehyde groups. Cells were then permeabilized with 0.2% (v/v) Triton X‐100 in PBS for 15min, washed at least twice with PBS, and blocked for 1h in blocking buffer (1% (w/v) BSA and 5% (v/v) FCS in PBS). Samples were incubated with anti‐YAP antibody (Santa Cruz Biotechnology, cat. no. sc‐101199; 1:50 in blocking buffer) for 2h at room temperature. After two washes with PBS and one wash with blocking buffer, samples were incubated with Alexa Fluor 488‐conjugated donkey anti‐mouse antibody (Thermo Fisher Scientific, cat. no. A‐21202; 1:250 in blocking buffer) for 1h. Before imaging, samples were washed at least three times with PBS.

### 4.12 Microscopy and Image Analysis

Images were acquired using a Zeiss LSM 900 upright confocal laser scanning microscope. YAP‐immunostained samples were imaged with a W Plan‐Apochromat 20×/1.0 objective. Z‐stacks were acquired with a 2μm step size and displayed as maximum‐intensity projections with identical intensity settings across compared conditions.

SensEnhance mechanoresponsive cells cultured on hydrogels with spatial differences in stiffness were imaged with a W Plan‐Apochromat 10×/0.5 objective. Z‐stacks were acquired with a 2μm step size and displayed as maximum‐intensity projections using identical intensity settings across compared conditions. Image analysis was performed using a custom MATLAB (R2023b, MathWorks) script. Full 3D CZI stacks were imported, and channels corresponding to d2EYFP and TagBFP were extracted. Cell segmentation was performed in 3D on the d2EYFP channel. Briefly, the d2EYFP volume was normalized, Gaussian‐smoothed, and thresholded using an Otsu‐based threshold calculated from the high‐intensity voxel population. The resulting 3D masks were cleaned by removing small objects, applying mild 3D closing, and watershed‐based splitting. Background fluorescence was estimated independently for each channel as the median intensity of voxels outside the segmented cell masks. Background‐subtracted d2EYFP and TagBFP fluorescence were quantified across the full 3D volume, and per‐cell mean fluorescence values were calculated. For each sample and stiffness region, the median of all per‐cell mean fluorescence values pooled across the corresponding fields of view was used for analysis. Maximum‐intensity projections with projected segmentation outlines were generated only for quality control and visualization; all fluorescence quantification was performed on the full 3D stacks.

### 4.13 Statistical Analysis

Comparisons between two groups were performed using unpaired two‐sided Welch's *t*‐tests. For comparisons involving more than two groups or multiple experimental factors, one‐way or two‐way ANOVA was used, as appropriate, followed by Tukey's multiple‐comparisons test. The exact statistical test, number and type of replicates (*n*), and definition of the plotted values are indicated in the corresponding figure legends. Unless stated otherwise, experiments were performed with at least three replicates. Statistical differences are reported as significant for p<0.05 and non‐significant for p≥0.05.

### 4.14 Software

Statistical analyses and data plotting were performed using GraphPad Prism 10 (GraphPad Software). Image processing and analysis were performed using ImageJ (v1.53t) and MATLAB (R2023b, MathWorks). Flow cytometry data analysis and visualization were performed using FlowJo (v10.0). Manuscript figures were assembled and formatted in Adobe Illustrator 2025. During manuscript preparation, the authors used ChatGPT and Microsoft Copilot to improve readability and language. All AI‐assisted text was reviewed and edited by the authors, who take full responsibility for the content of the publication.

## Author Contributions

M.B. and S.A. contributed equally to this work. M.K. and W.W. jointly supervised this work and share senior authorship. M.B., S.A., M.K. and W.W. conceptualized and designed the research. S.A. designed and validated the genetic circuit using constitutive promoters, and M.B. implemented the circuit with sensing promoters and performed all subsequent experimental work. P.G. and M.K. performed modeling and simulations, and M.K. carried out the mathematical analysis of the circuit. All authors analyzed the data. M.B., P.G., M.K. and W.W. wrote the manuscript with input from all authors. M.K. and W.W. acquired funding.

## Conflicts of Interest

The authors declare no conflicts of interest.

## Supporting information


**Supporting File**: advs78654‐sup‐0001‐SuppMat.pdf

## Data Availability

The data that support the findings of this study are available from the corresponding authors upon reasonable request.
