# Supplementary material for: SensEnhance: A Genetic Circuit for Converting Endogenous Mammalian Gene Signals Into High‐Dynamic‐Range Genetic Readouts
Source: Adv Sci (Weinh). 2026 Nov 9:e78654. Online ahead of print. doi: 10.1002/advs.78654 (PMC13748912; doi:10.1002/advs.78654)
Supplement: Supplementary file 1 [file ADVS-9999-e78654-s001.pdf]

Supporting Information

SensEnhance: A Genetic Circuit for Converting Endogenous Mammalian Gene Signals into High-Dynamic-Range Genetic Readouts

Miguel Baños[1,2,4], Stanislav Anastassov[3,4], Payman Goodarzi[1], Mustafa Khammash[3,5*], Wilfried Weber[1,2,5*]

[1]INM – Leibniz Institute for New Materials, Campus D2 2, 66123, Saarbrücken, Germany.

[2]Saarland University, Department of Materials Science and Engineering, Campus D2 2, 66123, Saarbrücken, Germany.

[3]Department of Biosystems Science and Engineering (D-BSSE), ETH Zurich, Klingelbergstrasse 48, 4056, Basel, Switzerland.

[4]These authors contributed equally to this work.

[5]These authors jointly supervised this work and share senior authorship.

*Corresponding author(s). E-mail(s): mustafa.khammash@bsse.ethz.ch; wilfried.weber@leibniz-inm.de;

Contents

1 Analysis of the Amplifier Circuit **2**

1.1 Nondimensionalization . . . 2

1.2 Steady-State Reduction and the Characteristic Cubic . . . 2

1.3 Amplifier Regime ($U \geq 1$): Uniqueness and Asymptotic Gain . . . 2

1.4 Leakage-Suppression Regime ($U < 1$): Uniqueness and Multistability . . . 3

1.5 Stability Analysis . . . 4

2 System Identification and General Optimization Framework **4**

2.1 Constitutive-promoter Circuit . . . 5

2.1.1 Lumped Identifiable Reparametrisation . . . 5

2.2 Mechanosensing Circuit . . . 8

2.3 IL-6 Sensing Circuit . . . 8

3 Supporting Figures **9**

4 Supporting Tables **21**

5 Plasmid DNA Sequences **25**

1 Analysis of the Amplifier Circuit

This supporting section provides the full mathematical analysis of the simplified circuit introduced in Section 2.2. We derive the steady-state characteristic equation, prove uniqueness of the operating point in the amplifier regime, characterize the asymptotic gain, and establish exact conditions under which the circuit can exhibit multistability in the leakage-suppression regime.

1.1 Nondimensionalization

We introduce dimensionless variables

$$\tau = \gamma t, \qquad x_1 = \frac{\gamma}{\alpha_q} z_1, \qquad x_2 = \frac{\eta}{\gamma} z_2, \qquad x_3 = \frac{\eta}{\gamma} z_3, \tag{S1}$$

and dimensionless parameters

$$U = \frac{u_s}{\alpha_q}, \qquad a = \frac{\eta \alpha_q}{\gamma^2}, \qquad b = \frac{\beta}{\gamma}. \tag{S2}$$

Here U is the normalized input, a measures the relative strength of intein-mediated sequestration, and b is the normalized downstream gain. Substituting into the circuit dynamics of Section 2.2 gives

$$\frac{dx_1}{d\tau} = U - x_1(1 + x_3), \tag{S3}$$

$$\frac{dx_2}{d\tau} = abx_1 - x_2(1 + x_3), \tag{S4}$$

$$\frac{dx_3}{d\tau} = a - x_3(1 + ax_1 + x_2). \tag{S5}$$

The system depends only on the three dimensionless parameters U, a, and b.

1.2 Steady-State Reduction and the Characteristic Cubic

Setting the right-hand sides of (S3)–(S5) to zero gives

$$U = x_1(1 + x_3), \qquad abx_1 = x_2(1 + x_3), \qquad a = x_3(1 + ax_1 + x_2). \tag{S6}$$

We define the effective loss factor

$$\mathcal{A} := 1 + x_3 = \frac{\eta z_3 + \gamma}{\gamma} \geq 1, \tag{S7}$$

since $x_3 \geq 0$. The first two relations in (S6) give immediately

$$x_1 = \frac{U}{\mathcal{A}}, \qquad x_2 = \frac{abU}{\mathcal{A}^2}. \tag{S8}$$

Substituting (S8) into the third relation and multiplying through by $\mathcal{A}^2$ yields the characteristic cubic

$$F(\mathcal{A}) = \mathcal{A}^3 + (aU - a - 1)\mathcal{A}^2 + aU(b - 1)\mathcal{A} - abU = 0, \qquad \mathcal{A} \geq 1. \tag{S9}$$

Physical equilibria of the circuit are in one-to-one correspondence with roots of F on the domain $\mathcal{A} \geq 1$. Once the positive root $\mathcal{A}^*$ is found, the steady-state concentrations follow from (S8) and the relation $x_3^* = \mathcal{A}^* - 1$, which in original variables read

$$z_1^* = \frac{u_s}{\gamma \mathcal{A}^*}, \qquad z_2^* = \frac{\beta u_s}{\gamma^2 (\mathcal{A}^*)^2}, \qquad z_3^* = \frac{\gamma}{\eta}(\mathcal{A}^* - 1). \tag{S10}$$

Note that $F(1) = 1 + (aU - a - 1) + aU(b - 1) - abU = -a < 0$ and $F(\mathcal{A}) \to +\infty$ as $\mathcal{A} \to \infty$, so there is always at least one physical equilibrium.

1.3 Amplifier Regime ($U \geq 1$): Uniqueness and Asymptotic Gain

Proposition 1 (Uniqueness in the amplifier regime) *For $U \geq 1$ and $b > 1$, the cubic F has exactly one root on $\mathcal{A} \geq 1$.*

Proof Differentiating (S9) gives

$$F'(\mathcal{A}) = 3\mathcal{A}^2 + 2(aU - a - 1)\mathcal{A} + aU(b-1), \tag{S11}$$

with second derivative $F''(\mathcal{A}) = 6\mathcal{A} + 2(aU - a - 1)$. For $\mathcal{A} \geq 1$ and $U \geq 1$,

$$F''(\mathcal{A}) \geq 6 + 2(a - a - 1) = 4 > 0, \tag{S12}$$

so F' is strictly increasing on $[1, \infty)$. Evaluating at $\mathcal{A} = 1$,

$$F'(1) = 1 + aU(b+1) - 2a = 1 + a\big[U(b+1) - 2\big]. \tag{S13}$$

For $U \geq 1$ and $b > 1$, we have $U(b+1) > 2$, so $F'(1) > 0$. Therefore $F'(\mathcal{A}) > 0$ for all $\mathcal{A} \geq 1$, and F is strictly increasing on $[1, \infty)$. Since $F(1) = -a < 0$ and $F(\mathcal{A}) \to +\infty$, the Intermediate Value Theorem guarantees exactly one root $\mathcal{A}^* > 1$. □

Asymptotic linear regime.

Rearranging (S9) gives

$$aU(\mathcal{A} - 1)(\mathcal{A} + b) = \mathcal{A}^2(a + 1 - \mathcal{A}). \tag{S14}$$

Since $\mathcal{A} \geq 1$ and $b > 0$, the left-hand side is non-negative, so $\mathcal{A} \leq a + 1$ and $\mathcal{A}$ is uniformly bounded. Dividing both sides of (S14) by U and taking $U \to \infty$, the right-hand side tends to zero, giving $\mathcal{A}(U) \to 1$. Therefore

$$x_1^*(U) = \frac{U}{\mathcal{A}(U)} \sim U \qquad (U \to \infty), \tag{S15}$$

and returning to original variables,

$$z_1^* \sim \frac{u_s}{\gamma}, \qquad z_2^* \sim \frac{\beta u_s}{\gamma^2} \qquad (u_s \to \infty). \tag{S16}$$

The output z_2^* grows linearly with input at a gain of β/γ^2, which exceeds the single-stage gain $1/\gamma$ by the factor β/γ when $\beta > \gamma$.

1.4 Leakage-Suppression Regime ($U < 1$): Uniqueness and Multistability

When $U < 1$, F' may be non-monotone on $[1, \infty)$, allowing up to three physical equilibria.

Sufficient condition for uniqueness.

If $a(1 - U) \leq 2$, then for all $\mathcal{A} \geq 1$,

$$F''(\mathcal{A}) = 6\mathcal{A} + 2(aU - a - 1) \geq 4 - 2a(1 - U) \geq 0, \tag{S17}$$

so F is convex on $[1, \infty)$. Combined with $F(1) < 0$ and $F(\mathcal{A}) \to +\infty$, this gives exactly one physical root.

Critical points.

When $\Delta > 0$, where

$$\Delta := \big(a(1 - U) + 1\big)^2 - 3aU(b - 1), \tag{S18}$$

the derivative F' has two real critical points

$$\mathcal{A}_\pm = \frac{a(1 - U) + 1 \pm \sqrt{\Delta}}{3}, \qquad \mathcal{A}_- < \mathcal{A}_+, \tag{S19}$$

where $\mathcal{A}_-$ is a local maximum and $\mathcal{A}_+$ a local minimum of F.

Proposition 2 (Exact condition for multistability) *The circuit possesses three distinct physical equilibria if and only if*

$$\Delta > 0, \qquad \mathcal{A}_- > 1, \qquad F(\mathcal{A}_-) > 0, \qquad F(\mathcal{A}_+) < 0. \tag{S20}$$

Equivalently, F must have two turning points in the physical domain $[1, \infty)$ with the local maximum above and the local minimum below the horizontal axis.

Proof Since $F(1) < 0$ and $F(\mathcal{A}) \to +\infty$, F always has at least one root on $[1, \infty)$. If $\Delta \leq 0$, then F' has at most one real root, so F is monotone or has a single inflection and can cross zero only once. If $\Delta > 0$ but $\mathcal{A}_- \leq 1$, the local maximum lies outside the physical domain and only one root is possible. Three distinct physical roots require $\mathcal{A}_- > 1$, which places both turning points inside $[1, \infty)$. In this case F increases on $[1, \mathcal{A}_-]$, decreases on $[\mathcal{A}_-, \mathcal{A}_+]$, and increases on $[\mathcal{A}_+, \infty)$. It crosses zero three times on $[1, \infty)$ if and only if the local maximum is positive and the local minimum is negative, giving conditions (S20). □

Illustrative example.

The parameter choice $U = 0.2$, $a = 20$, $b = 20$ gives

$$F(\mathcal{A}) = \mathcal{A}^3 - 17\mathcal{A}^2 + 76\mathcal{A} - 80 = (\mathcal{A} - 5)\big(\mathcal{A}^2 - 12\mathcal{A} + 16\big)\,, \tag{S21}$$

with three physical roots

$$\mathcal{A}_1 = 6 - 2\sqrt{5} \approx 1.528, \qquad \mathcal{A}_2 = 5, \qquad \mathcal{A}_3 = 6 + 2\sqrt{5} \approx 10.472. \tag{S22}$$

1.5 Stability Analysis

The Jacobian of the dimensionless dynamics (S3)–(S5) evaluated at a steady state (x_1^*, x_2^*, x_3^*) is

$$J = \begin{pmatrix} -(1+x_3^*) & 0 & -x_1^* \\ ab & -(1+x_3^*) & -x_2^* \\ -ax_3^* & -x_3^* & -(1+ax_1^*+x_2^*) \end{pmatrix}. \tag{S23}$$

For the three-equilibrium example above, the eigenvalues of J at each equilibrium are:

Equilibrium 1 ($\mathcal{A}_1 \approx 1.528$): $\lambda \approx -0.3717,\ -2.1763,\ -38.3964$. All eigenvalues are real and negative; this equilibrium is *locally asymptotically stable.*

Equilibrium 2 ($\mathcal{A}_2 = 5$): $\lambda \approx 0.2989,\ -7.6494 \pm 2.2491\,i$. One positive real eigenvalue; this equilibrium is an *unstable saddle.*

Equilibrium 3 ($\mathcal{A}_3 \approx 10.472$): $\lambda \approx -0.3622,\ -11.3468 \pm 2.5283\,i$. All eigenvalues have negative real part; this equilibrium is *locally asymptotically stable.*

The outer two equilibria are therefore stable and the middle one is unstable, which is the standard bistable configuration.

2 System Identification and General Optimization Framework

To identify the parameters of the genetic amplifier, we used a common internal circuit model for all three experimental implementations and allowed the mapping from experimental conditions to effective production rates to vary across circuits. Specifically, the internal dynamics of the three core species z_1, z_2, and z_3 were assumed to be preserved, while the stimulus input $u(s)$ and control reference input $q(c)$ were defined according to the biological implementation of each experiment. This separation was necessary because the internal sequestration amplification architecture is shared across the three circuits, whereas the experimental realization of the sensing and reference arms differs between the constitutive promoter validation, the IL-6 sensing circuit, and the mechanosensing circuit. Accordingly, all three circuits were described by the shared core model:

$$\dot{z}_1 = \alpha_1 + u(s) - \eta z_3 z_1 - \gamma z_1, \tag{S24}$$
$$\dot{z}_2 = \alpha_2 + h(z_1) - \eta z_2 z_3 - \gamma z_2, \tag{S25}$$
$$\dot{z}_3 = \alpha_3 + q(c) - \eta z_3(z_1 + z_2) - \gamma z_3. \tag{S26}$$

where α_i denote basal production rates, η is the intein-mediated sequestration rate, γ is the effective degradation and dilution rate, and $h(z_1)$ is the transcriptional activation function of the second stage. In this formulation, the circuit-specific differences enter only through the input functions $u(s)$ and $q(c)$, whereas the internal circuit structure and the reporter equation remain unchanged.

We first assessed the structural identifiability of this system, a theoretical mathematical property determining whether model parameters can be uniquely recovered from ideal, noise-free, continuous observations of the output reporter r under transient conditions. Using differential algebra via the `StructuralIdentifiability.jl` package in Julia (approximating the Hill coefficient as a rational integer to permit symbolic computation), we confirmed that all system parameters, including the sequestration rate η and the degradation rate γ, are globally structurally identifiable. This provided rigorous mathematical proof that the underlying differential equations do not possess intrinsic symmetries that would permanently prevent parameter isolation.

Having established theoretical structural identifiability, practical identifiability was then addressed. Structural identifiability guarantees unique solutions only for continuous time-series data; however, available steady-state dose-response data compress dynamic temporal information, often rendering parameters practically unidentifiable.

To infer the model parameters, we formulated a general parameter optimization framework applied across all circuits. Parameter estimation was defined as a nonlinear least squares problem, minimizing the residuals between the steady-state model predictions and the corresponding experimental measurements. The optimization was implemented in Python using the `SciPy` package, utilizing the Trust Region Reflective algorithm with stringent convergence tolerances (`xtol` and `ftol` set to 10^{-8}). To examine the stability of the optimization against different starting points and to ensure robust convergence to the global minimum rather than local minima, we employed a multi-start optimization strategy. We generated 150 random initial parameter guesses spanning the biologically plausible bounds. To efficiently cover this parameter space, we used Latin Hypercube Sampling (LHS) via the `SciPy` module. Furthermore, to evaluate the predictive power of the model and detect overfitting, we employed replicate-level leave-one-out cross-validation (LOOCV), systematically withholding one replicate at a time and refitting the model to the remaining data. The specific steady-state solutions, residual weighting, and practical identifiability constraints utilized for each experimental implementation are detailed in the subsequent circuit-specific sections.

2.1 Constitutive-promoter Circuit

For the constitutive-promoter validation circuit shown in Figure 2, the effective production rates of the activator and inhibitor arms were modeled as linear functions of the corresponding transfected plasmid masses:

$$u(s) = a \cdot s, \quad q(c) = b \cdot c, \tag{S27}$$

where $s = m_{Z1}$ and $c = m_{Z3}$ denote the transfected masses of the activator and inhibitor plasmids, respectively, and a, b are effective plasmid-to-production conversion factors. The transcriptional activation term was written as:

$$h(z_1) = V \frac{z_1^n}{K^n + z_1^n}. \tag{S28}$$

Under these assumptions, the steady-state equations become:

$$\begin{aligned}
0 &= a \cdot s + \alpha_1 - \eta z_{1,\mathrm{ss}} z_{3,\mathrm{ss}} - \gamma z_{1,\mathrm{ss}}, \\
0 &= V \frac{z_{1,\mathrm{ss}}^n}{K^n + z_{1,\mathrm{ss}}^n} + \alpha_2 - \eta z_{2,\mathrm{ss}} z_{3,\mathrm{ss}} - \gamma z_{2,\mathrm{ss}}, \\
0 &= b \cdot c + \alpha_3 - \eta z_{3,\mathrm{ss}} (z_{1,\mathrm{ss}} + z_{2,\mathrm{ss}}) - \gamma z_{3,\mathrm{ss}}, \\
r_{\mathrm{ss}} &= \frac{\alpha_r + V \frac{z_{1,\mathrm{ss}}^n}{K^n + z_{1,\mathrm{ss}}^n}}{\gamma}.
\end{aligned} \tag{S29}$$

2.1.1 Lumped Identifiable Reparametrisation

Since the original parameters $(\eta, \gamma, V, a, b, \alpha_i)$ are not individually identifiable from steady-state dose–response data, we work instead with the following lumped reparametrisation.

We consider α_i negligible, $\alpha_i \approx 0$. To eliminate the redundancy, we introduced the rescaled state variables:

$$w_i = \frac{\eta}{\gamma} z_i, \quad i \in \{1, 2, 3\}, \tag{S30}$$

and defined the four lumped parameters together with the fixed ratio $R_\eta = \eta/\gamma$:

$$C_a = \frac{\eta}{\gamma^2} b, \quad C_b = \frac{\eta}{\gamma^2} a, \quad R_V = \frac{V}{\gamma}, \quad K_t = \frac{\eta}{\gamma} K. \tag{S31}$$

Substituting (S30) into (S29) and solving for the states yields the equivalent steady-state system expressed entirely in terms of the lumped parameters:

$$w_3 = \frac{C_a\, c}{1 + w_1 + w_2}, \tag{S32}$$

Table S1. Model parameters and cross-fold stability metrics for the basal activator cross-validation ($R_\eta = 0.1$, $n = 1.5$, $a_1 = 0.4055$ fixed using $c = 0$ data). The fitted parameters (C_a, C_b, RV, and K_t) for each fold are reported in the table along with their mean, half-range, and percentage variation.

Left-out concentration	C_a	C_b	R_V	K_t	Train RMSE	Test RMSE
$c = 1.0$	5.0269	0.1814	1.0936	1.2320	0.0329	0.0219
$c = 4.0$	4.8802	0.1873	1.0810	1.2338	0.0341	0.0114
$c = 16.0$	4.9841	0.1902	1.0804	1.2433	0.0345	0.0051
Mean ± half-range	4.9637 ± 0.0734	0.1863 ± 0.0044	1.0850 ± 0.0066	1.2364 ± 0.0057	—	—
Variation (%)	3.0%	4.7%	1.2%	0.9%	—	—

$$w_1 = \frac{C_b\, s}{1 + w_3}, \tag{S33}$$

$$w_2 = \frac{R_\eta R_V}{1 + w_3} \frac{w_1^n}{K_t^n + w_1^n}, \tag{S34}$$

$$r_{\text{ss}} = R_V \frac{w_1^n}{K_t^n + w_1^n}. \tag{S35}$$

In this formulation the non-identifiable parameter γ has been eliminated entirely; the model depends only on the four identifiable lumped combinations (C_a, C_b, R_V, K_t), the Hill coefficient n, and the regularizing ratio R_η. Profile-likelihood analysis of the six-parameter model $(C_a, C_b, R_V, K_t, n, R_\eta)$ revealed a residual practical non-identifiability (Figure S1).

Due to the practical non-identifiability of the full system parameters, a subset of parameters is fixed to nominal or physically motivated values prior to validation. Specifically, the feedback-related dilution rate ratio R_η (which can be independently characterized via transient experiments) is set to a nominal value of 0.1. The Hill cooperativity exponent n is fixed to 1.5 to reflect a physically meaningful cooperative activator system ($n > 1$).

To isolate the basal parameters, the basal activator expression level a_1 is estimated exclusively from the dataset without feedback ($c = 0.0$), while the rest of basal feedback expression parameters a_i set to 0.0. Using these fixed values, we validate the model by performing a leave-one-inhibitor-level-out cross-validation. In each fold, we hold out one non-zero inhibitor concentration $c \in \{1.0, 4.0, 16.0\}$ as a test set, and fit the remaining free parameters (C_a, C_b, RV, and K_t) on the remaining training data (consisting of $c = 0.0$ and the other two inhibitor levels). The out-of-sample model predictions and associated errors are illustrated in Figure S2, while the estimated parameter values and their cross-fold variations are reported in Table S1.

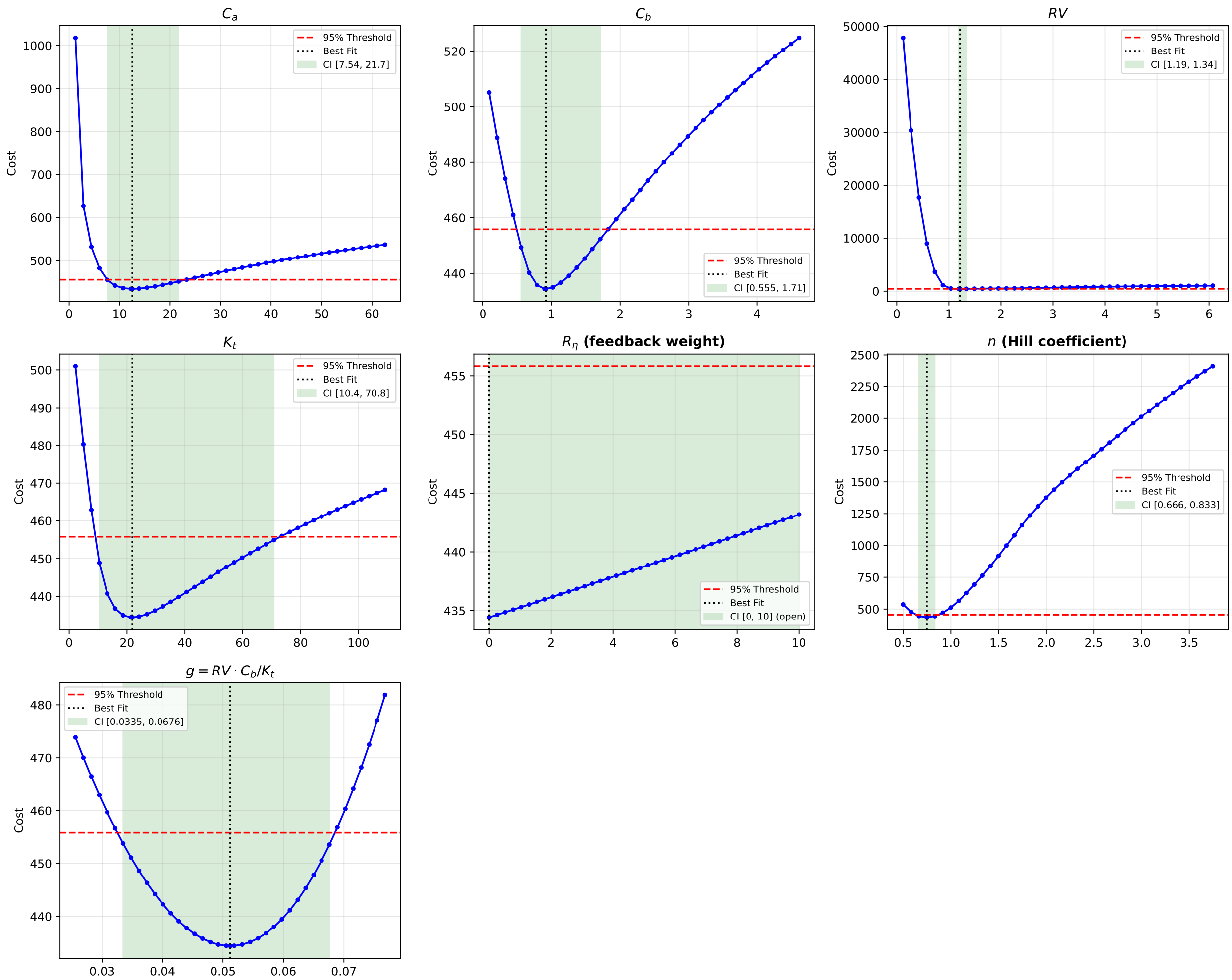

Figure S1. Profile-likelihood analysis for the constitutive-promoter validation circuit with six free parameters $(C_a, C_b, R_V, K_t, n, R_\eta)$.

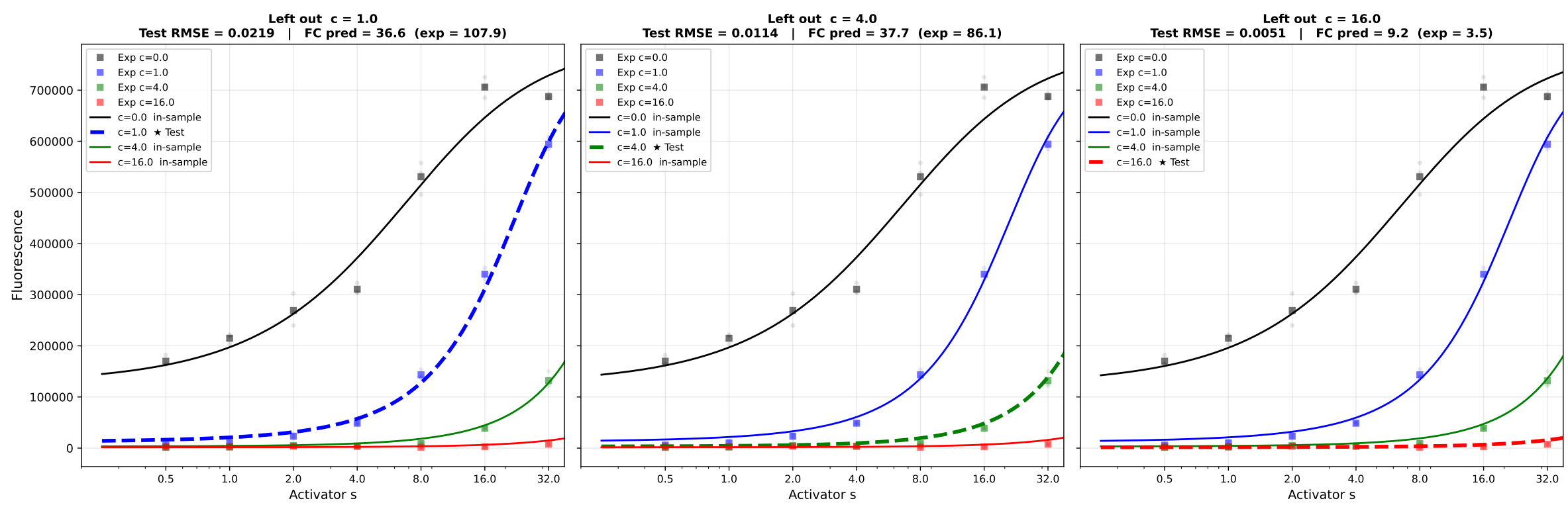

Figure S2. Leave-one-inhibitor-concentration-out cross-validation predictions for the basal activator model ($R_\eta = 0.1$, $n = 1.5$, $a_1 = 0.4055$). Each panel shows the model curves compared to experimental replicates (small circles) and replicate means (squares) when the indicated inhibitor level $c \in \{1.0, 4.0, 16.0\}$ is held out of the training set. In-sample training predictions are shown as solid lines, and the out-of-sample test prediction is highlighted as a thick dashed line. The out-of-sample Test RMSE and predicted raw fold changes (with experimental values in parentheses) are indicated at the top of each panel.

As the evaluation showed, the base circuit is not practically identifiable. Therefore, we do not perform system identification for the more sophisticated IL-6 and mechanosensing circuits. The following subsections present only

the models for those circuits, which are used in this manuscript to generate prediction plots of system output versus input for different c values, based on the fitted models and available measurements.

2.2 Mechanosensing Circuit

For the mechanosensing circuit shown in Figure 3, both the sensor and reference arms are controlled by stimulus-responsive promoters. The stimulus input $u(s)$ and the control reference input $q(c)$ were each modeled by Hill-type functions:

$$u(s) = V_u \frac{s^{n_u}}{K_u^{n_u} + s^{n_u}}, \qquad q(c) = V_q \frac{c^{n_q}}{K_q^{n_q} + c^{n_q}}, \tag{S36}$$

where s denotes substrate stiffness, c the doxycycline concentration, V_u and V_q the respective maximal production rates, K_u and K_q the half-saturation constants, and n_u, n_q the Hill coefficients of each arm.

2.3 IL-6 Sensing Circuit

For the IL-6 sensing circuit shown in Figure 5, the effective production rates of the activator and inhibitor arms were modeled as:

$$u(\mathrm{IL6}) = V_u \frac{\mathrm{IL6}^{n_u}}{K_u^{n_u} + \mathrm{IL6}^{n_u}}, \qquad q(c) = b \cdot c, \tag{S37}$$

where IL6 denotes the IL-6 concentration, c is the inhibitor plasmid ratio, V_u is the maximal activator production rate, K_u is the half-saturation constant, n_u is the Hill coefficient of the sensing arm, and b is the proportionality constant relating inhibitor plasmid ratio to effective inhibitor production.

3 Supporting Figures

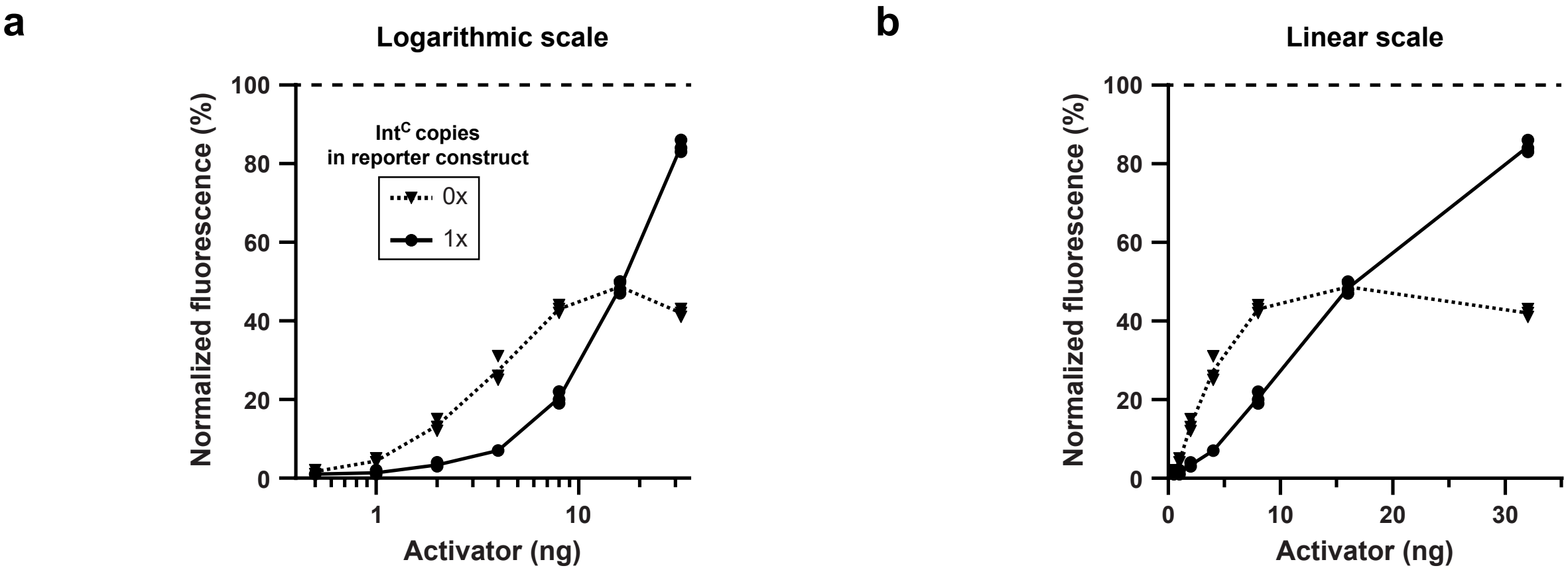

Figure S3. Contribution of output-driven $\mathrm{Int}^{\mathrm{C}}$ to reporter expression. a,b, Flow cytometry analysis of mVenus fluorescence in HEK-293T cells transfected with SensEnhance ($1 \times \mathrm{Int}^{\mathrm{C}}$ in the reporter construct) or the single sequestration circuit lacking output-driven $\mathrm{Int}^{\mathrm{C}}$ ($0 \times \mathrm{Int}^{\mathrm{C}}$). Cells were transfected with a fixed amount of reporter construct, increasing amounts of activator construct (0.5, 1, 2, 4, 8, 16 and 32 ng), and 1 ng of quencher construct. Activator and quencher expression were both driven by EF1α promoters. Fluorescence was measured 24 h after transfection. For each circuit, fluorescence was normalized to the highest value measured for the same circuit without quencher across the tested activator range, defined as 100%. The same data are shown with a logarithmic (**a**) or linear (**b**) activator axis. Each symbol represents the normalized median fluorescence of one technical replicate (n = 3), and lines connect the replicate means.

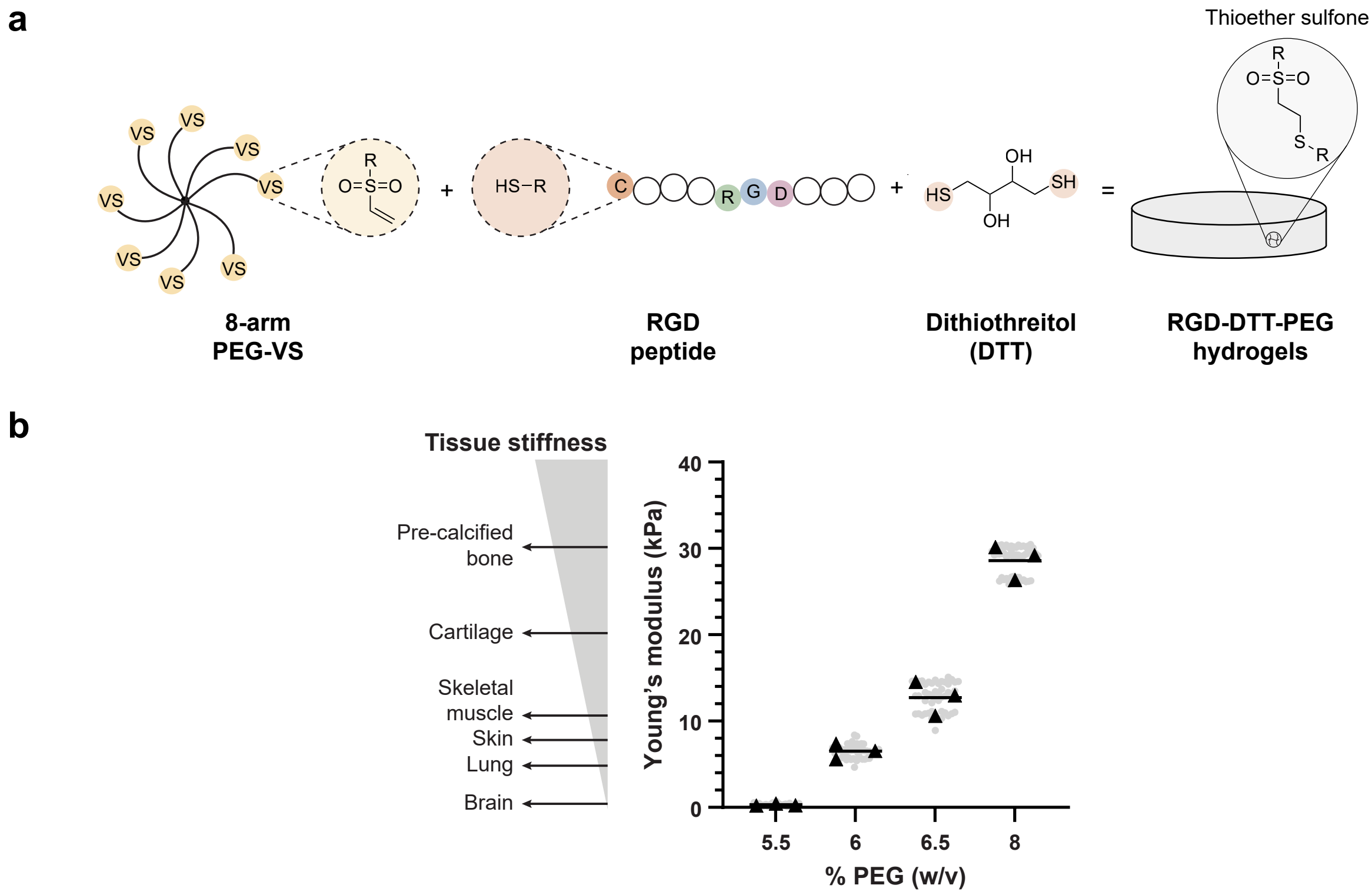

Figure S4. RGD-DTT-PEG hydrogel formation and mechanical characterization. a, Schematic representation of RGD-DTT-PEG hydrogel formation by Michael-type addition. Vinyl sulfone groups on 8-arm polyethylene glycol-vinyl sulfone (PEG-VS) react with thiol groups from cysteine-functionalized RGD peptide to support cell adhesion, or with thiol groups from dithiothreitol (DTT), which acts as the crosslinker. The thiol–vinyl sulfone reaction forms thioether sulfone bonds, resulting in an RGD-functionalized 3D hydrogel network. **b**, Mechanical characterization of RGD-DTT-PEG hydrogels by nanoindentation. Hydrogel stiffness was tuned by varying the final PEG-VS concentration (5.5, 6, 6.5 and 8% w/v), generating hydrogels with Young's moduli ranging from approximately 0.3 to 28 kPa. This range covers stiffness values reported for many soft tissues [1]. Dots indicate individual measurements, triangles indicate hydrogel means ($n = 3$ independent hydrogel batches), and horizontal lines indicate replicate means.

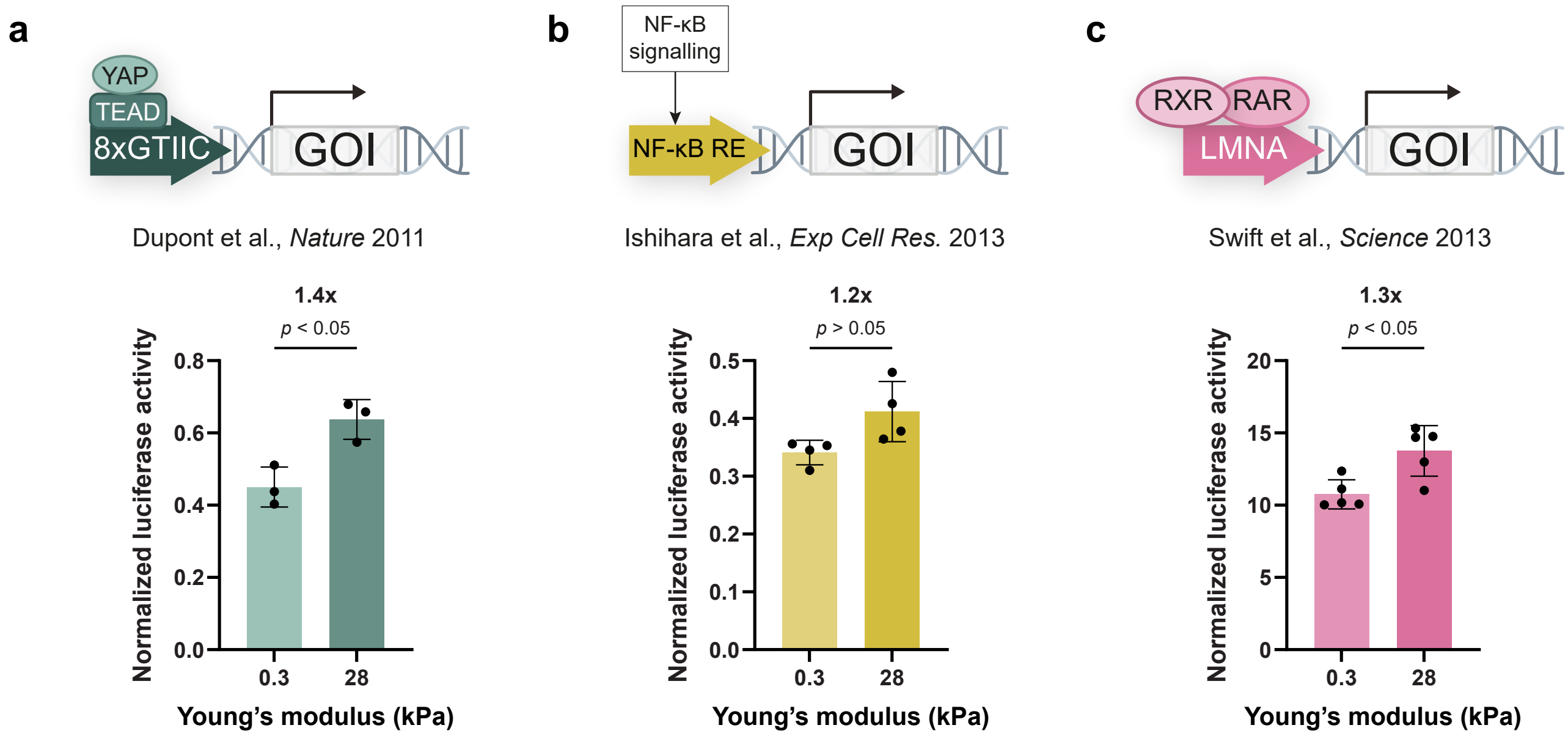

Figure S5. Dual-luciferase screening of candidate mechanoresponsive promoters as SensEnhance inputs. NIH/3T3 fibroblasts were transfected with firefly luciferase reporters driven by promoters previously described as mechanoresponsive: the YAP/TAZ-responsive 8xGTIIC promoter [2] (**a**), an NF-κB-responsive promoter [3] (**b**), and the lamin A/C gene (*LMNA*) promoter [4] (**c**). A constitutively expressed Renilla luciferase reporter was co-transfected for normalization. Twenty-four hours after transfection, cells were seeded on RGD-DTT-PEG hydrogels with Young's moduli of 0.3 or 28 kPa. Forty-eight hours after seeding, cells were lysed, and luminescence was measured. Each dot represents the normalized firefly/Renilla luciferase activity of cells from one independently prepared hydrogel replicate ($n = 3$ for 8xGTIIC, $n = 4$ for NF-κB, and $n = 5$ for *LMNA*). Bars represent the mean of the replicate values, and error bars indicate standard deviation. Statistical significance was assessed using unpaired two-sided Welch's t-tests.

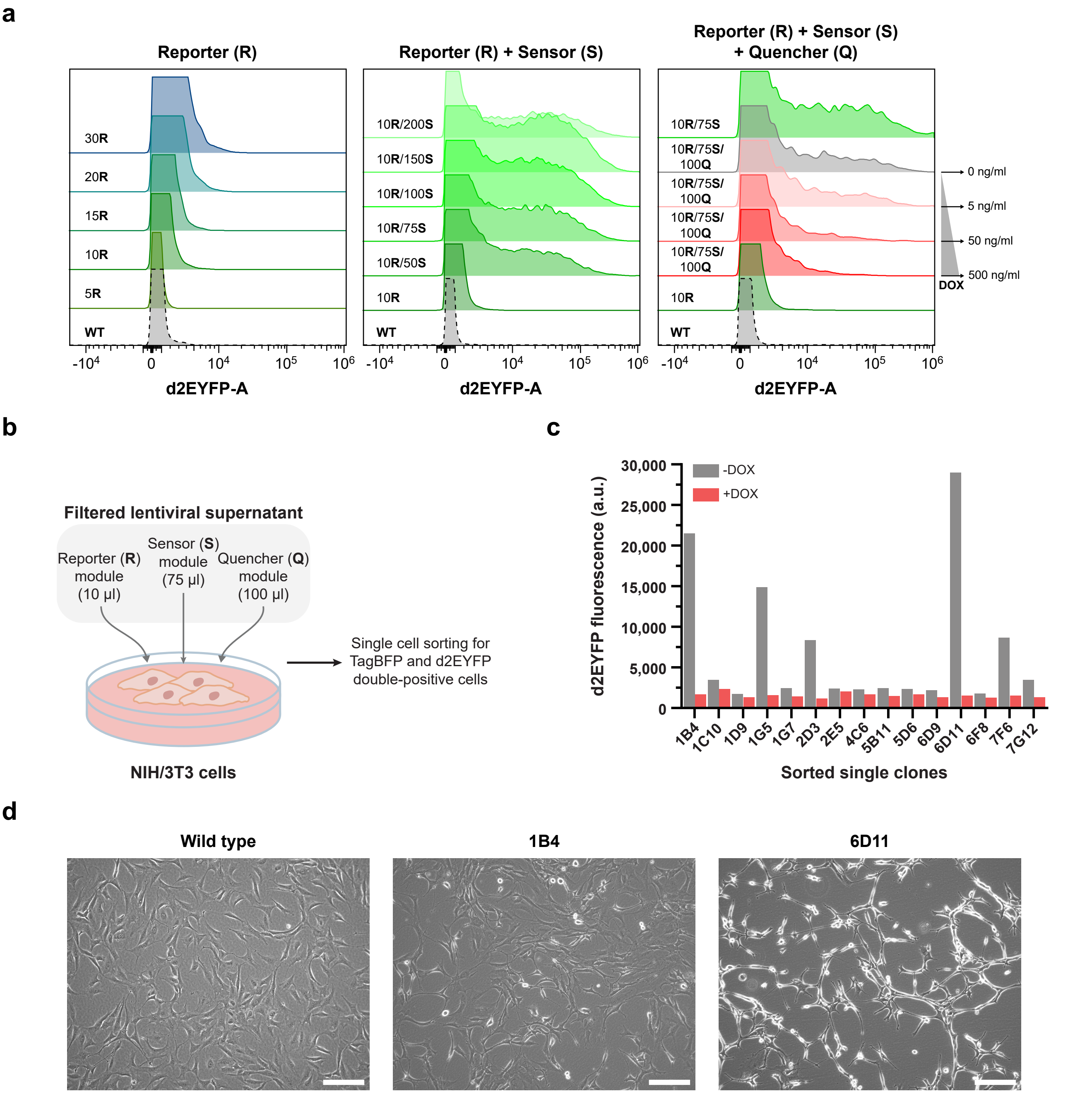

Figure S6. Lentiviral transduction and screening of monoclonal SensEnhance mechanoresponsive cell lines. a, Flow cytometry analysis of d2EYFP fluorescence in unsorted NIH/3T3 populations transduced with the reporter construct alone (left), reporter and sensor constructs (middle), or reporter, sensor and quencher constructs (right). R, S and Q denote the reporter, sensor and quencher modules, respectively; the preceding numbers indicate the volumes of filtered lentiviral supernatant used for each module in μL. **b**, Schematic representation of the transduction and sorting workflow. NIH/3T3 fibroblasts were transduced using 10, 75 and 100 μL of filtered lentiviral supernatant encoding the reporter, sensor and quencher constructs, respectively. Double-positive TagBFP$^+$/d2EYFP$^+$ cells were isolated by fluorescence-activated single cell sorting and expanded as single-cell-derived clones. **c**, Preliminary flow cytometry screening of SensEnhance clones without or with 1000 ng mL^{-1} doxycycline. Bars show median d2EYFP fluorescence for each clone and condition. Clone 1B4 was selected for high reporter fluorescence, doxycycline-induced suppression, and typical NIH/3T3 morphology and growth. **d**, Representative images of wild type (WT) NIH/3T3 cells and clones 1B4 and 6D11 cultured on tissue culture plastic. Scale bars are 200 μm.

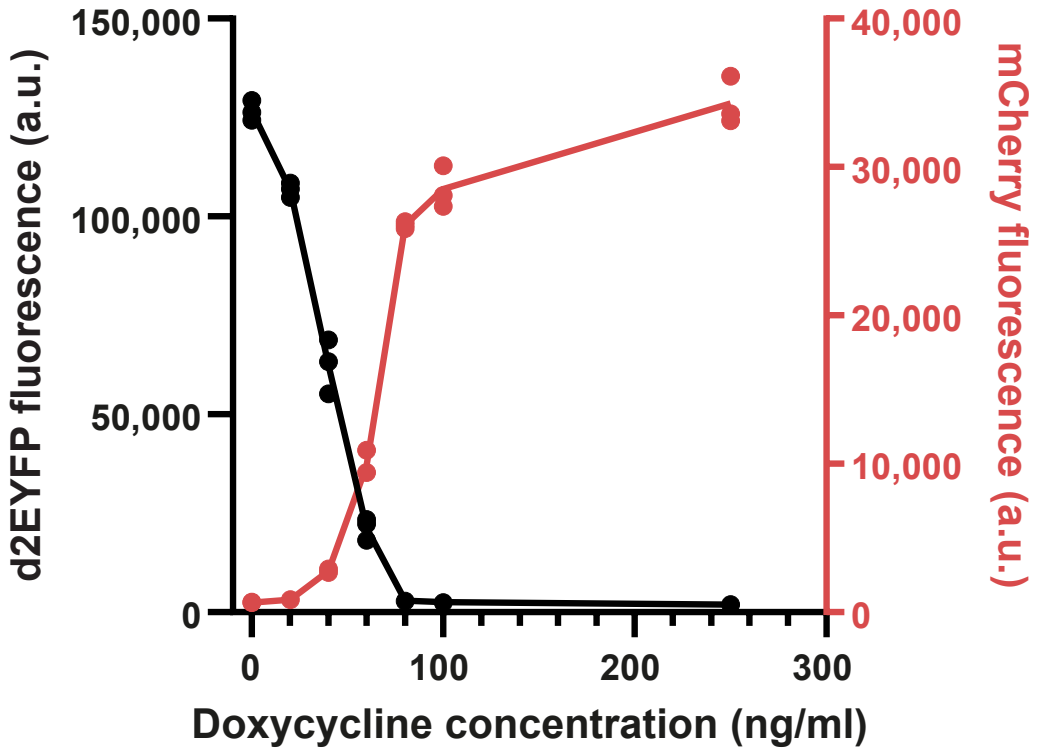

Figure S7. Doxycycline-dependent tuning of SensEnhance circuit output. Flow cytometry analysis of d2EYFP fluorescence, which reports full SensEnhance circuit output, and mCherry fluorescence, used as a proxy for doxycycline-induced $\mathrm{Int}^{\mathrm{N}}$ expression, as a function of doxycycline concentration. SensEnhance mechanoresponsive cells were seeded on tissue-culture plastic, where circuit activation is maximal. Sixteen hours after seeding, the medium was replaced with complete medium containing 0, 20, 40, 60, 80, 100 or 250 ng mL^{-1} doxycycline. After 48 h, cells were detached and analyzed by flow cytometry. Each dot represents the median fluorescence of one technical replicate ($n = 3$). Lines connect the mean fluorescence values of the replicates.

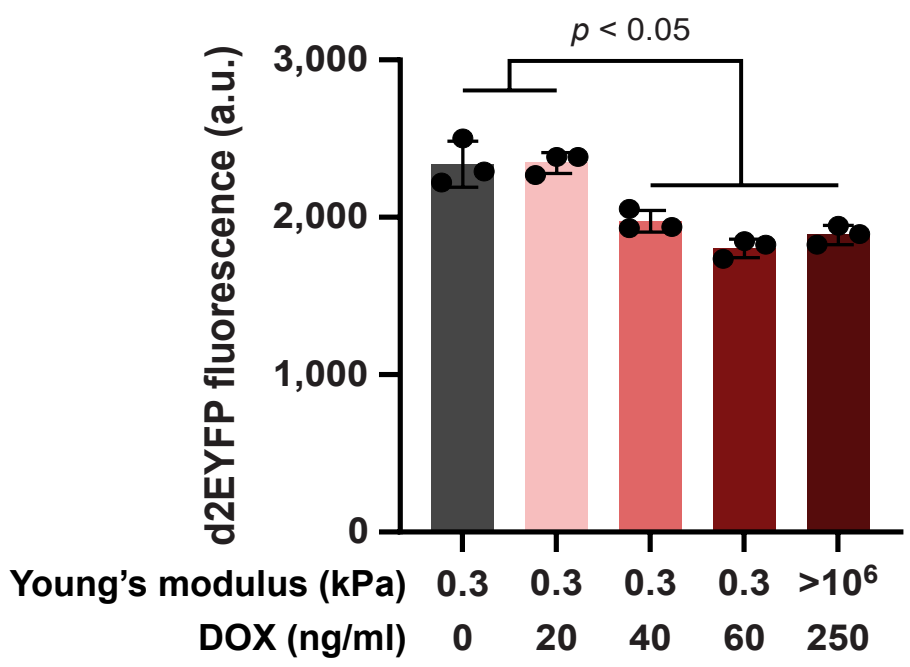

Figure S8. SensEnhance mechanoresponsive cells approach the lower circuit output state on soft hydrogels. Flow cytometry analysis of d2EYFP fluorescence in SensEnhance mechanoresponsive cells cultured on soft RGD-DTT-PEG hydrogels (0.3 kPa) and treated with increasing doxycycline concentrations (0, 20, 40 and 60 ng mL^{-1}). Tissue-culture plastic treated with 250 ng mL^{-1} doxycycline was included as a strongly quenched reference condition. Although doxycycline further reduced d2EYFP fluorescence on soft hydrogels, reporter output was already close to the lower circuit output state in the absence of doxycycline. Each dot represents the median fluorescence of cells from one independently prepared hydrogel replicate or well ($n = 3$). Bars represent the mean of the replicate values, and error bars indicate standard deviation. Statistical significance was assessed by one-way ANOVA followed by Tukey's multiple-comparisons test.

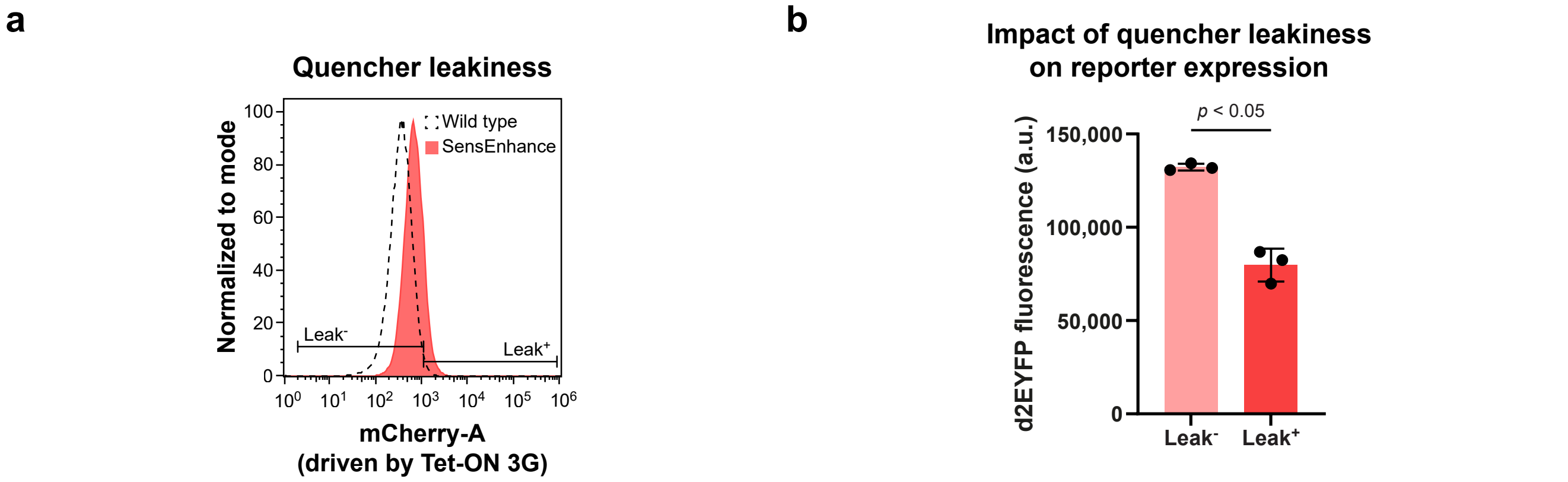

Figure S9. Impact of leaky $\mathrm{Int}^{\mathrm{N}}$ expression on reporter output in SensEnhance mechanoresponsive cells. **a**, Representative flow cytometry histograms of mCherry fluorescence in SensEnhance mechanoresponsive cells cultured on tissue-culture plastic without doxycycline, with wild type NIH/3T3 cells as a reference. mCherry was used as a proxy for expression of the Tet-On 3G-controlled $\mathrm{Int}^{\mathrm{N}}$ quencher module. Wild type cells were used to define the gates separating mCherry-negative (Leak^{-}) and mCherry-positive (Leak^{+}) SensEnhance subpopulations. **b**, Flow cytometry analysis of d2EYFP fluorescence in the Leak^{-} and Leak^{+} subpopulations defined in **a**. Each dot represents the median fluorescence of the indicated subpopulation in one technical replicate ($n = 3$). Bars represent the mean of the replicate values, and error bars indicate standard deviation. Statistical significance was assessed using an unpaired two-sided Welch's t-test.

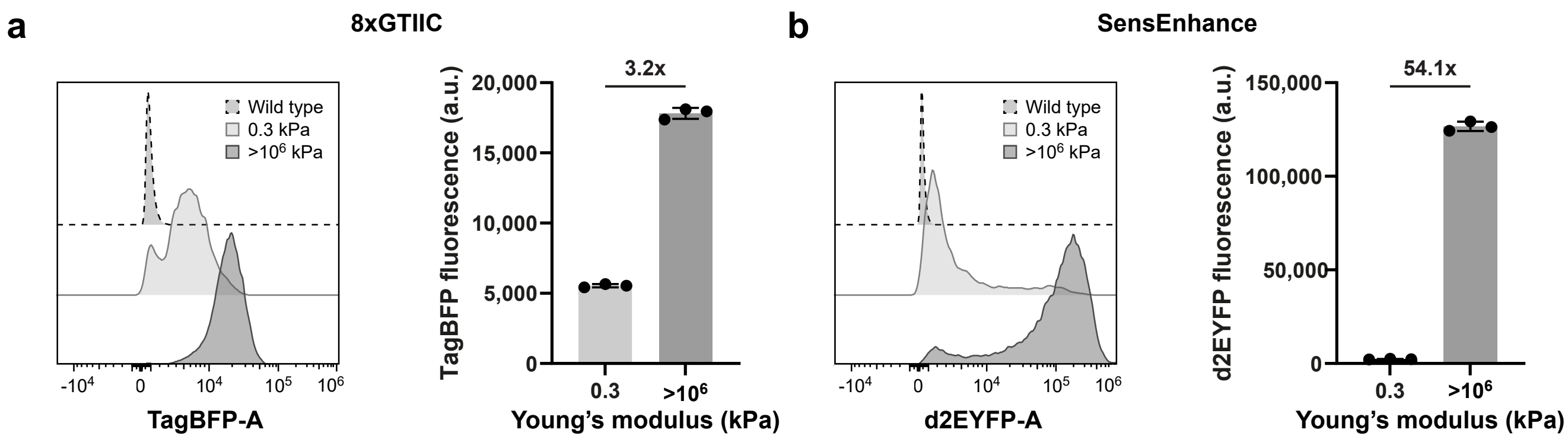

Figure S10. SensEnhance expands the dynamic range of the mechanosensing output across the widest stiffness range tested. **a**, Flow cytometry analysis of TagBFP fluorescence driven by the 8xGTIIC mechanoresponsive promoter in SensEnhance mechanoresponsive cells cultured on soft RGD-DTT-PEG hydrogels (0.3 kPa) or tissue-culture plastic ($> 10^6$ kPa). Representative TagBFP fluorescence histograms are shown for each condition together with wild type NIH/3T3 cells as a reference. In the bar plot, each circle represents the median TagBFP fluorescence of cells from one independently prepared hydrogel replicate or well ($n = 3$). **b**, Flow cytometry analysis of d2EYFP fluorescence as the full SensEnhance circuit output under the same stiffness conditions as in **a**. Representative d2EYFP fluorescence histograms are shown for each condition together with wild type NIH/3T3 cells as a reference. In the bar plot, each circle represents the median d2EYFP fluorescence of cells from one independently prepared hydrogel replicate or well ($n = 3$). Bars represent the mean of the replicate values, and error bars indicate standard deviation. Fold changes are indicated.

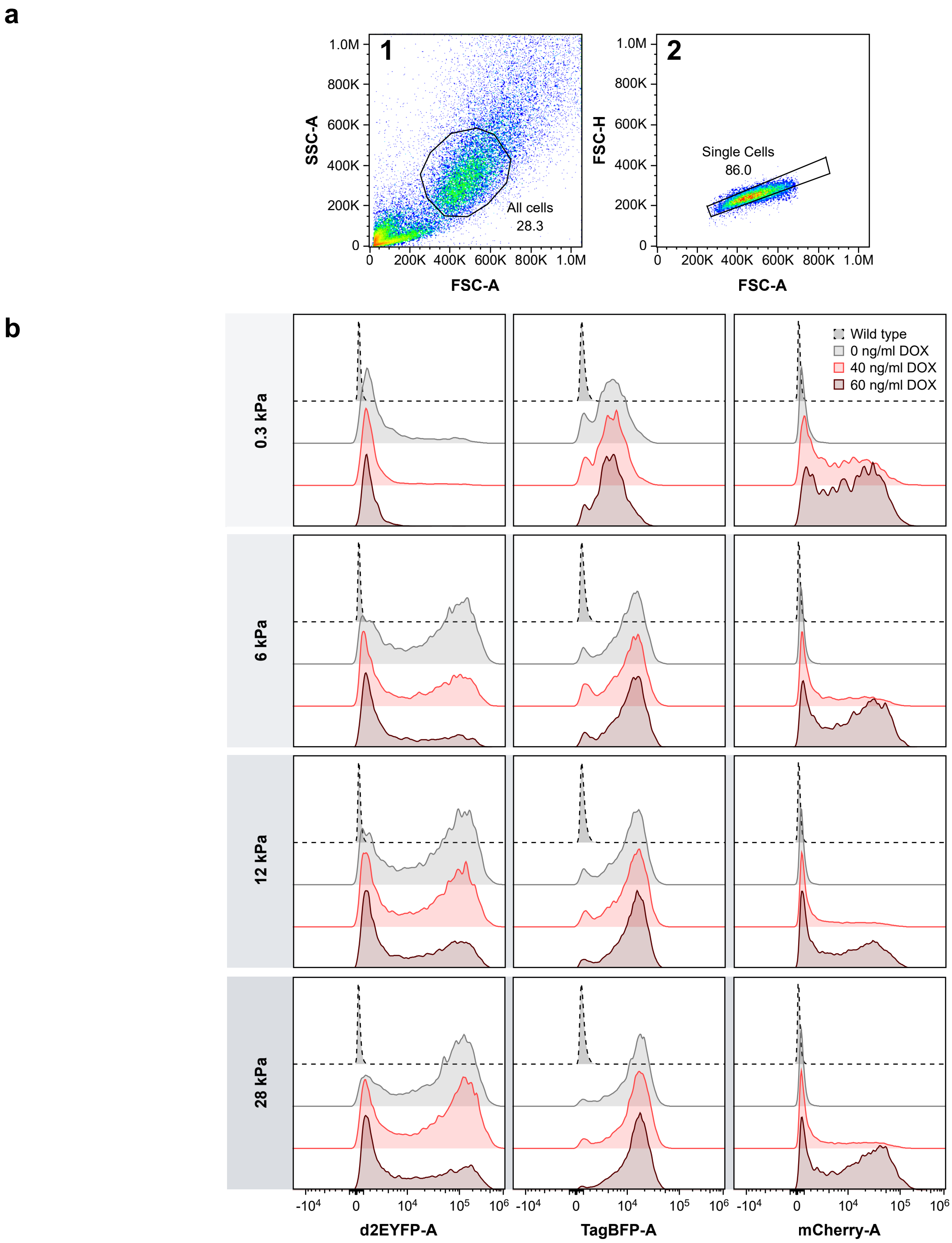

Figure S11. Flow cytometry analysis of SensEnhance mechanoresponsive NIH/3T3 cells cultured on hydrogels with different Young's moduli. a, Representative gating strategy used for fluorescence analysis of SensEnhance mechanoresponsive NIH/3T3 cells. The main cell population was first selected based on forward and side scatter (dot plot 1), followed by singlet gating (dot plot 2). The resulting single-cell population was used for subsequent fluorescence quantification. **b**, Representative fluorescence histograms of gated SensEnhance mechanoresponsive cells cultured on hydrogels with different Young's moduli and treated with 0, 40 or 60 ng mL^{-1} doxycycline. Histograms show the d2EYFP, TagBFP and mCherry channels. Wild type NIH/3T3 cells are shown as a fluorescence-negative reference.

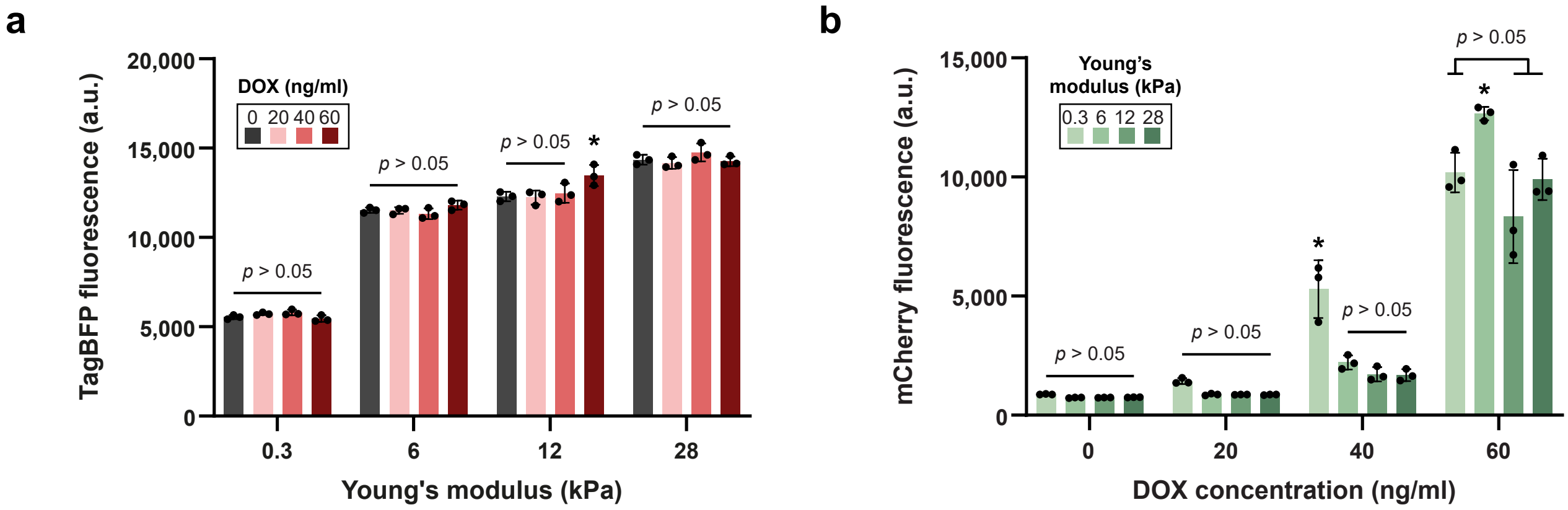

Figure S12. Doxycycline and matrix-stiffness effects on SensEnhance input-promoter and quenching-arm reporters. a, Flow cytometry analysis of TagBFP fluorescence in SensEnhance mechanoresponsive cells cultured on RGD-DTT-PEG hydrogels with Young's moduli of 0.3, 6, 12 and 28 kPa and treated with 0, 20, 40 or 60 ng mL^{-1} doxycycline. **b**, Flow cytometry analysis of mCherry fluorescence in SensEnhance mechanoresponsive cells under the same hydrogel-stiffness and doxycycline conditions. Each circle represents the median fluorescence of cells from one independently prepared hydrogel replicate (n = 3). Bars represent the mean of the replicate values, and error bars indicate standard deviation. Asterisks indicate significant differences ($p < 0.05$) for the indicated comparisons, as determined by two-way ANOVA followed by Tukey's multiple-comparisons test.

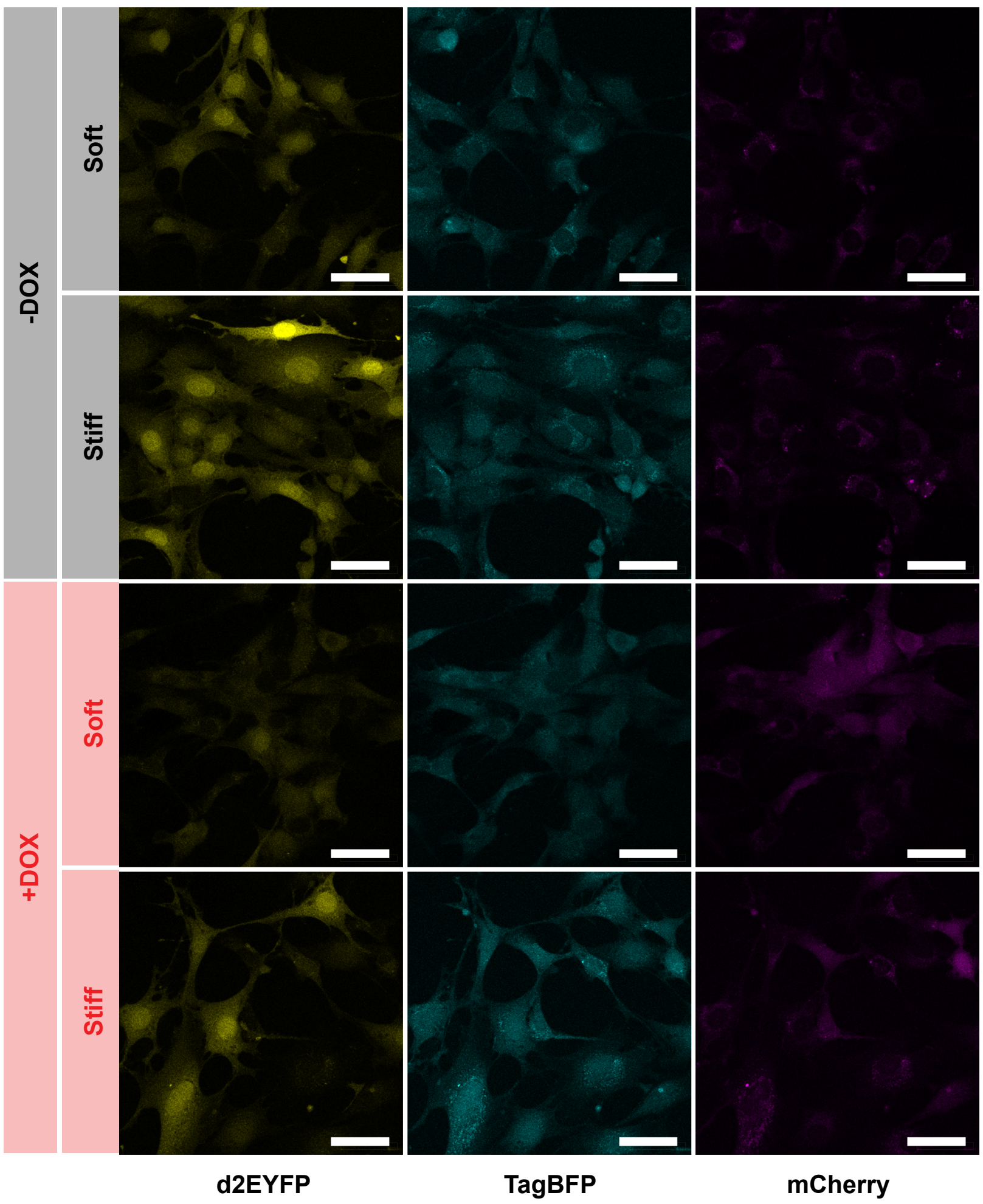

Figure S13. Representative confocal microscopy images of SensEnhance mechanoresponsive cells on spatially patterned hydrogels. SensEnhance mechanoresponsive cells were cultured on soft and stiff regions of spatially patterned hydrogels in the absence or presence of 40 ng mL^{-1} doxycycline. The soft and stiff regions had Young's moduli of approximately 2.5 and 28 kPa, respectively. Cells were fixed 48 h after seeding. Scale bars are 50 μm.

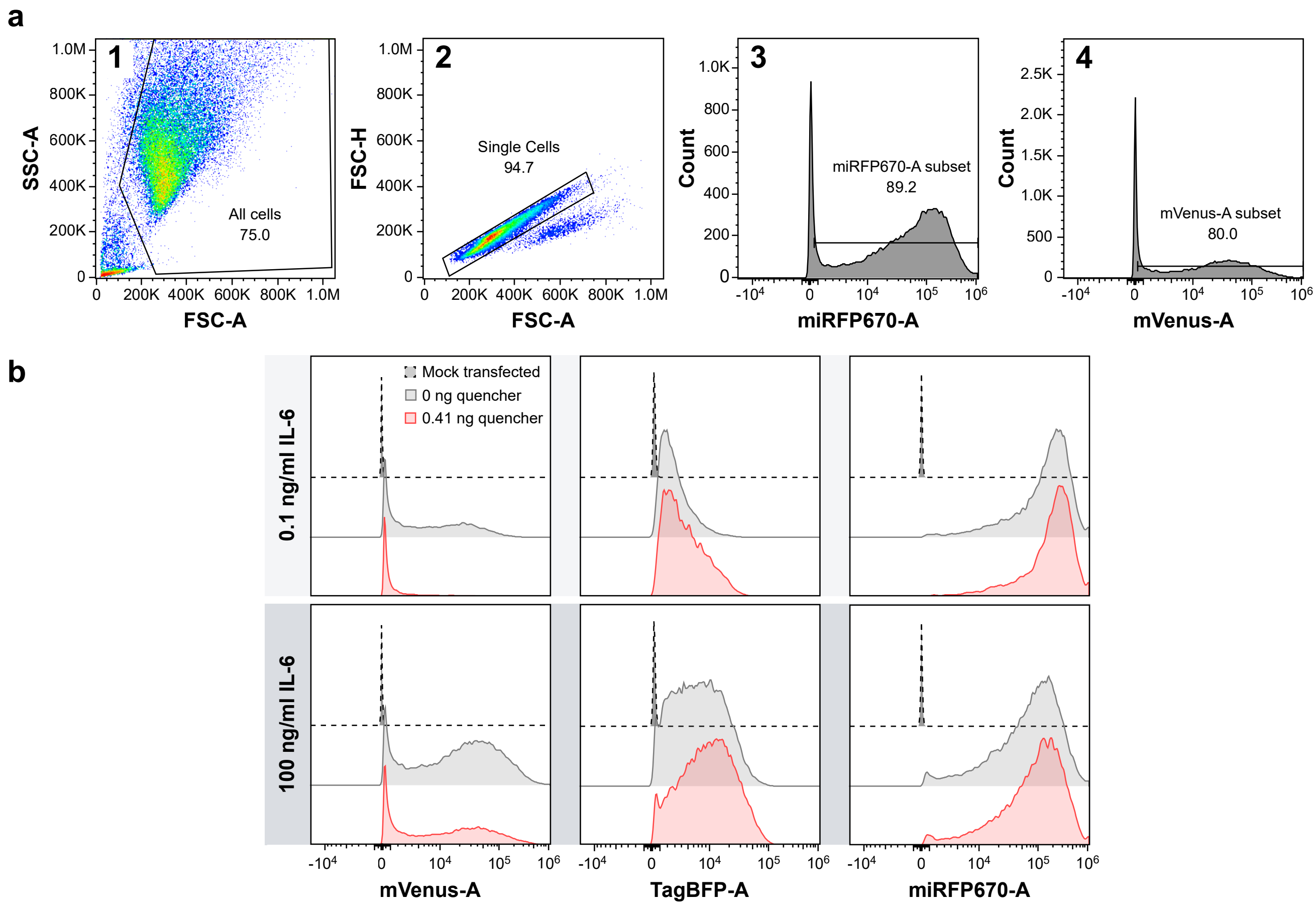

Figure S14. Flow cytometry analysis of SensEnhance HEK-293T cells. a, Representative gating strategy used for fluorescence analysis of SensEnhance HEK-293T cells. The main cell population was first selected based on forward and side scatter (dot plot 1), followed by singlet gating (dot plot 2). miRFP670$^+$/mVenus$^+$ double-positive single cells were selected as the transfected SensEnhance cell population used for subsequent fluorescence quantification (histograms 3 and 4). Histogram gates were defined using mock-transfected cells as the negative control and applied consistently across conditions. **b**, Representative fluorescence histograms of gated SensEnhance IL-6-sensing cells stimulated with low or high IL-6 concentrations (0.1 and 100 ng mL^{-1}), showing the mVenus, TagBFP and miRFP670 channels for cells transfected with 0 or 0.41 ng quencher construct. Mock-transfected cells are shown as a fluorescence-negative reference.

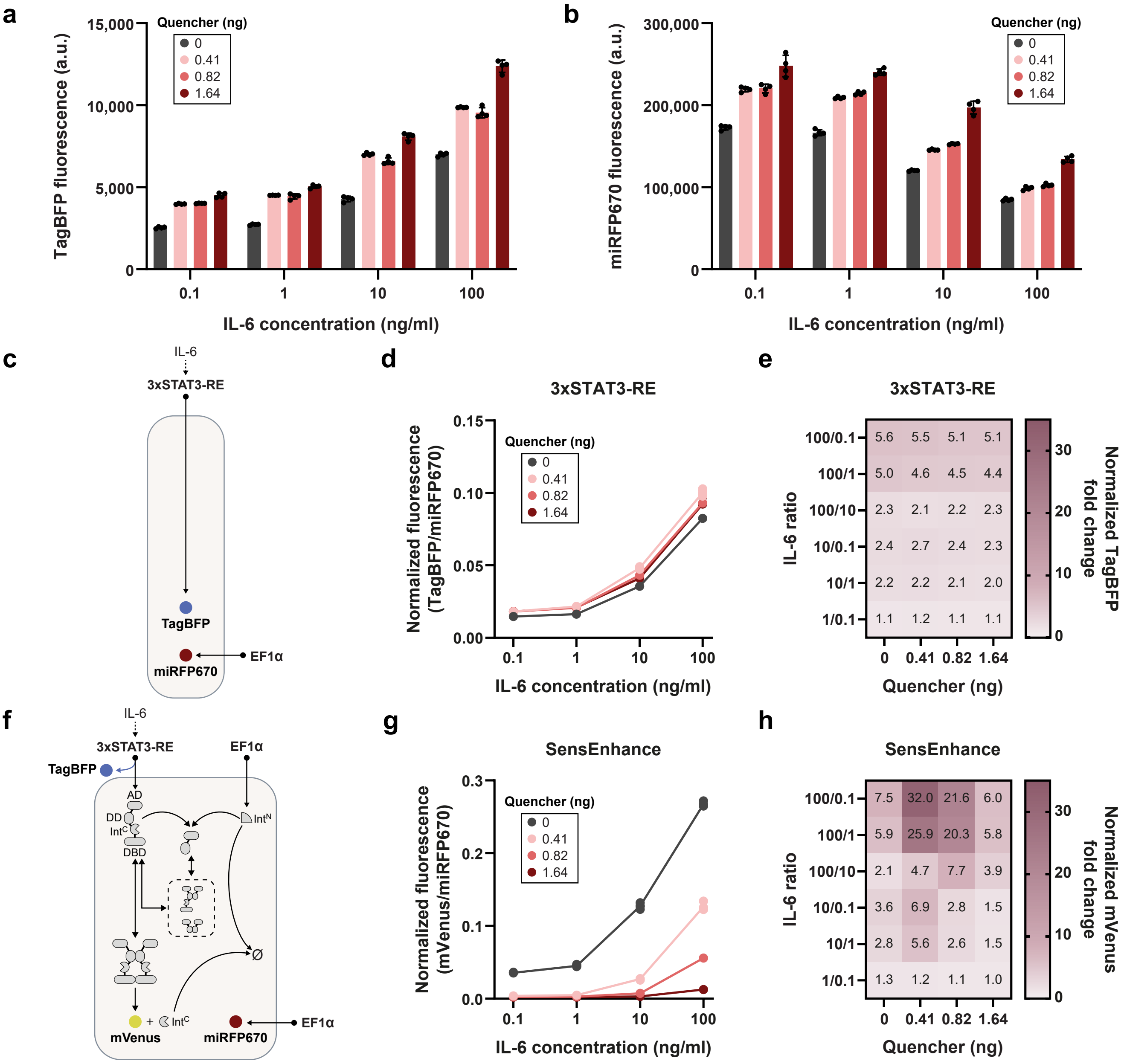

Figure S15. Quencher and IL-6 effects on SensEnhance input, output and normalization reporters. a, Flow cytometry analysis of TagBFP fluorescence in cells transfected with the 3×STAT3-RE-driven SensEnhance circuit, increasing amounts of quencher construct (0, 0.41, 0.82 and 1.64 ng), and stimulated with IL-6 (0.1, 1, 10 and 100 ng mL^{-1}) in the presence of 200 ng mL^{-1} sIL-6Rα. **b**, Flow cytometry analysis of miRFP670 fluorescence, constitutively driven by the EF1α promoter, under the same conditions as in **a**. To account for the transfection- or burden-associated variation observed in **a** and **b**, TagBFP fluorescence driven by the 3×STAT3-RE promoter (**c**) and mVenus fluorescence from the full circuit output (**f**) were normalized to the corresponding miRFP670 fluorescence. Normalized TagBFP fluorescence showed minimal variation across quencher conditions (**d**, **e**), whereas the SensEnhance-mediated improvement in IL-6-dependent reporter output was retained after normalization (**g**, **h**). Each circle represents the median fluorescence of one technical replicate (n = 4). Bars represent the mean of the replicate values, and error bars indicate standard deviation.

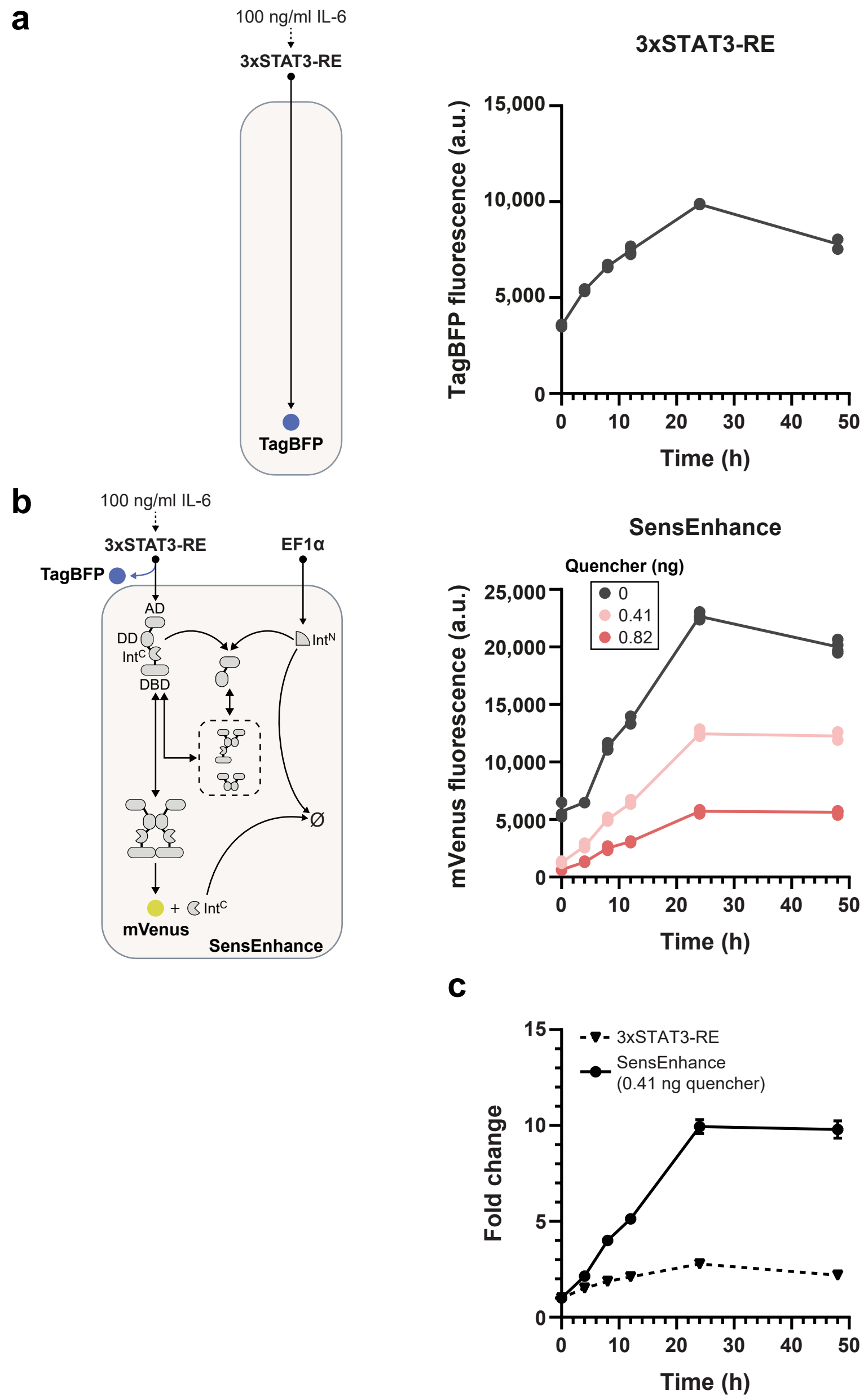

Figure S16. Time-dependent reporting of IL-6 stimulation by SensEnhance. **a**, Schematic and flow cytometry analysis of TagBFP fluorescence driven by the 3×STAT3-RE promoter in transiently transfected HEK-293T cells. Fluorescence was measured at the indicated times following stimulation at $t = 0$ with 100 ng mL^{-1} IL-6 in the presence of 200 ng mL^{-1} sIL-6Rα. **b**, Schematic and flow cytometry analysis of mVenus fluorescence as SensEnhance output in transiently transfected HEK-293T cells under the same stimulation conditions as in **a**. Cells were transfected with fixed amounts of activator and reporter constructs and increasing amounts of quencher construct (0, 0.41 and 0.82 ng). For **a** and **b**, each dot represents the median fluorescence of one technical replicate ($n = 4$), and lines connect the replicate means. **c**, Fold changes over time in TagBFP fluorescence driven by the 3×STAT3-RE promoter alone (dotted line, triangles) and mVenus fluorescence from SensEnhance with 0.41 ng quencher construct (solid line, circles). For each reporter, fold changes were calculated relative to its fluorescence at $t = 0$. Error bars indicate propagated standard errors of the mean.

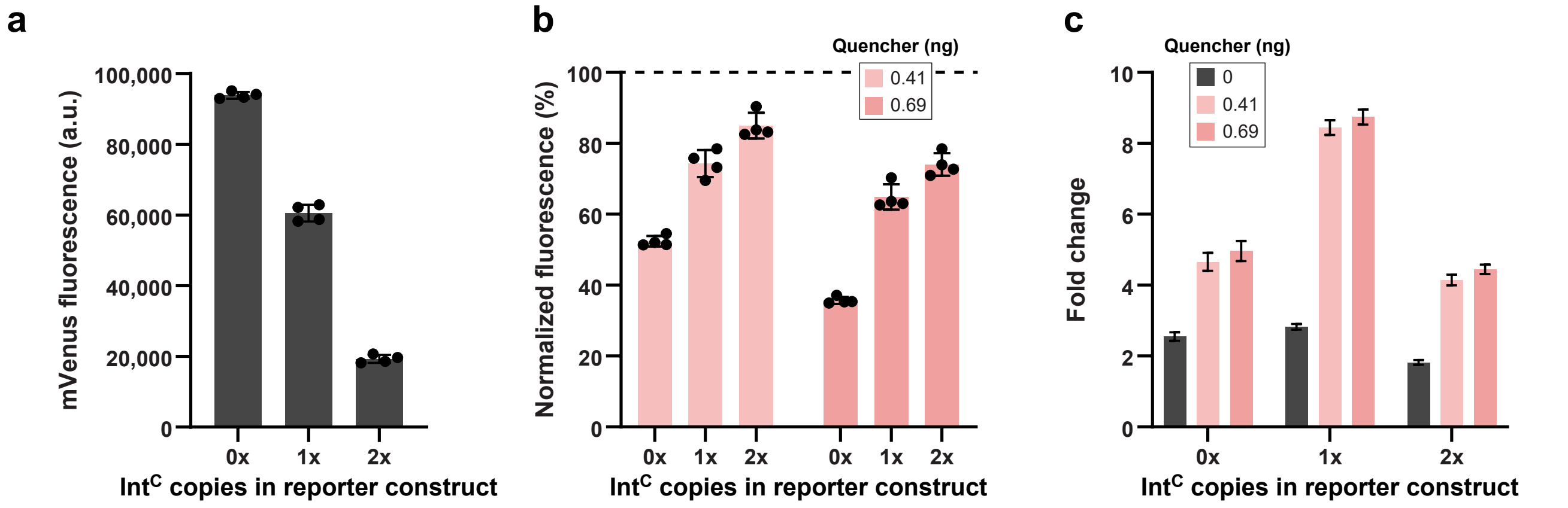

Figure S17. Impact of reporter-encoded $\mathrm{Int}^{\mathrm{C}}$ **copy number on relief of quenching in the IL-6-responsive SensEnhance circuit. a**, Flow cytometry analysis of mVenus fluorescence in transiently transfected HEK-293T cells containing the 3×STAT3-RE-driven SensEnhance circuit with reporter constructs encoding mVenus alone ($0 \times \mathrm{Int}^{\mathrm{C}}$) or together with one ($1{\times}\mathrm{Int}^{\mathrm{C}}$; SensEnhance) or two $\mathrm{Int}^{\mathrm{C}}$ copies ($2{\times}\mathrm{Int}^{\mathrm{C}}$). Cells were transfected with fixed amounts of activator and reporter constructs without quencher construct. Fluorescence was measured 24 h after stimulation with 100 ng mL^{-1} IL-6 in the presence of 200 ng mL^{-1} sIL-6Rα. **b**, Normalized mVenus fluorescence of the same circuit variants co-transfected with 0.41 or 0.69 ng quencher construct under the same stimulation conditions as in **a**. Fluorescence was normalized to the average fluorescence of the corresponding reporter variant without quencher, defined as 100%. For **a** and **b**, each dot represents the mean or normalized mean fluorescence, respectively, of one technical replicate (n = 4). Bars represent the mean of the replicate values, and error bars indicate standard deviation. **c**, Fold changes in mVenus fluorescence between 100 and 0.1 ng mL^{-1} IL-6 for each reporter variant and quencher construct amount (0, 0.41 and 0.69 ng). Bars represent fold changes, and error bars indicate propagated standard errors of the mean.

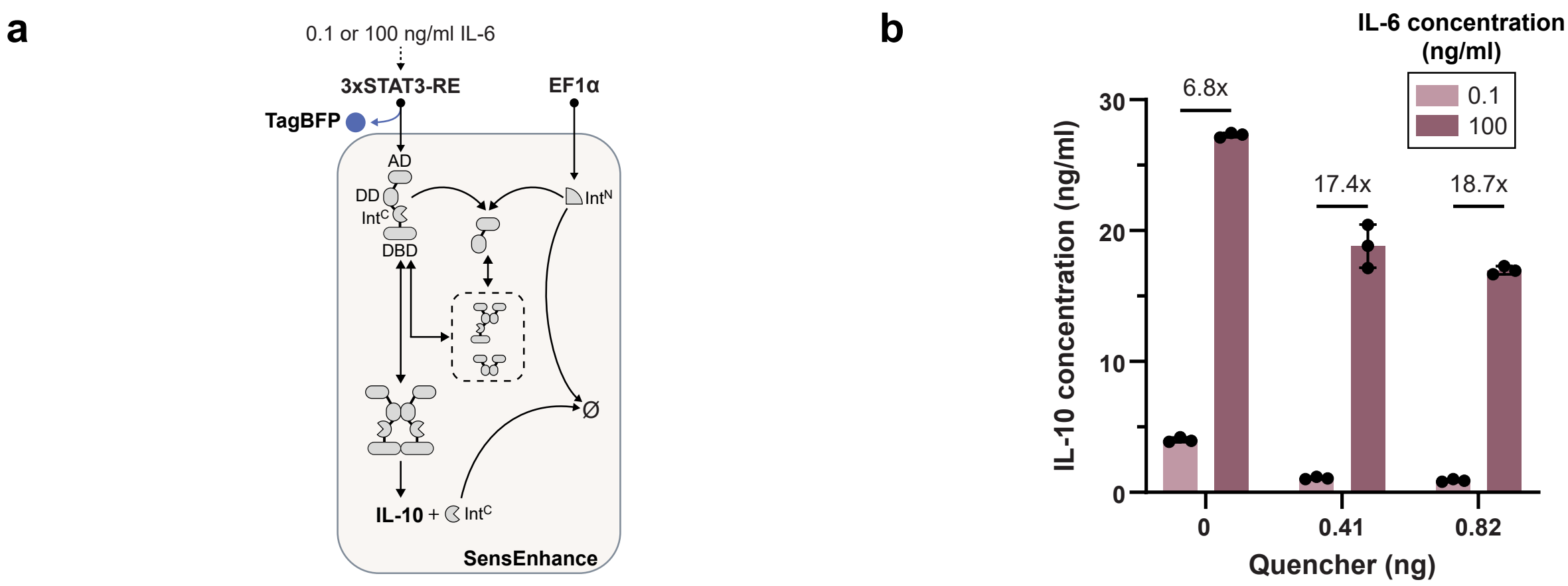

Figure S18. IL-10 production by the IL-6-responsive SensEnhance circuit. a, Schematic of the 3×STAT3-RE-driven SensEnhance circuit with human IL-10 as a representative secreted output protein. **b**, ELISA quantification of human IL-10 in culture supernatants of HEK-293T cells transfected with the circuit shown in **a**. Cells were transfected with fixed amounts of sensor and effector constructs and increasing amounts of quencher construct (0, 0.41 and 0.82 ng). At 24 h after transfection, cells were stimulated with 0.1 or 100 ng mL^{-1} IL-6 in the presence of 200 ng mL^{-1} sIL-6Rα. Culture supernatants were collected 24 h after stimulation. Each dot represents the IL-10 concentration in the supernatant from one separately transfected well (n = 3). Bars represent the mean of the replicate values, and error bars indicate standard deviation. Fold changes between conditions are indicated.

4 Supporting Tables

Table S2. Plasmid list.

Name	Category	Description	Backbone	Source
SA286	Activator	$P_{EF1\alpha}$–Gal4(DBD)–IntC–Gal4(DD)–VPR	pYTK095	[5]
SA720	Quencher	$P_{EF1\alpha}$–SUMO–IntN	pYTK095	This study
SA264	Reporter	5×UAS–P_{Min}–IntC–P2A–T2A–mVenus	pYTK095	[5]
SA480	Reporter	5×UAS–P_{Min}–mVenus	pYTK095	[5]
TTF138	Normalization	$P_{EF1\alpha}$–miRFP670	pGLM180	[6]
GL171	Stuffer	Empty vector	pGLM53	[7]
8xGTIIC	Luciferase reporter	8xGTIIC–P_{minTnT}–Firefly luciferase	pGL3b	[2]
NF-κB-Luc	Luciferase reporter	NF-κB-RE–P_{Min}–Firefly luciferase	[8]	[9]
pMB007	Luciferase reporter	P_{LMNA}–Firefly luciferase	pGL3b	This study
pRL-CMV	Normalization	P_{CMV}–Renilla luciferase	pRL-CMV	–
pMB025k	Reporter	5×UAS–P_{Min}–IntC–P2A–T2A–d2EYFP	pCDH	This study
pMB029	Quencher	P_{hPGK}–Tet-On 3G; P_{SV40}–PuroR; P_{TRE3GS}–SUMO–IntN–P2A–T2A–mCherry	pLVX-TetOne-Puro	This study
pMB042	Activator	8xGTIIC–P_{minTnT}–Gal4(DBD)–IntC–Gal4(DD)–VPR–P2A–T2A–TagBFP	pCDH	This study
pLTR-G	Lentiviral envelope	HIV-1 LTR–VSV-G	pEGFP-C1	[10]
pCD/NL-BH*DDD	Lentiviral packaging	P_{CMV}–HIV-1 gag/pol, Tat, Rev	pCDNA3.1	[11]
pMB091	Activator	3×STAT3-RE–P_{minCMV}–Gal4(DBD)–IntC–Gal4(DD)–VPR–P2A–T2A–TagBFP	pCDNA3.1	This study
pMB122	Effector	5×UAS–P_{Min}–IntC–P2A–T2A–hIL-10	SA264	This study
pMB124	Reporter	5×UAS–P_{Min}–IntC–P2A–IntC–P2A–T2A–mVenus	SA264	This study

Table S3. Transfection mixes used for the dual-luciferase assay of mechanoresponsive promoters on soft and stiff hydrogels shown in Figure S5. Amounts are given per 100 ng total DNA.

YAP/TAZ-responsive promoter	NF-κB-responsive promoter	*LMNA* promoter
8xGTIIC: 46.67 ng<br>pRL-CMV: 20 ng<br>GL171: 33.33 ng	NF-κB-Luc: 46.67 ng<br>pRL-CMV: 20 ng<br>GL171: 33.33 ng	pMB007: 46.67 ng<br>pRL-CMV: 20 ng<br>GL171: 33.33 ng

Table S4. Transfection mixes used for the constitutive-promoter SensEnhance validation shown in Figure 2b. For comparison with the circuit lacking output-driven IntC (Figure S3), the same mixes were used for the 0 and 1 ng SA720 conditions, with SA480 replacing SA264 and all DNA amounts unchanged. Amounts are given per 100 ng total DNA.

Increasing quencher (z_3) $\longrightarrow$

Increasing activator (z_1) $\longrightarrow$

SA286: 32 ng<br>SA720: 0 ng<br>SA264: 2.5 ng<br>TTF138: 5 ng<br>GL171: 60.5 ng	SA286: 32 ng<br>SA720: 1 ng<br>SA264: 2.5 ng<br>TTF138: 5 ng<br>GL171: 59.5 ng	SA286: 32 ng<br>SA720: 4 ng<br>SA264: 2.5 ng<br>TTF138: 5 ng<br>GL171: 56.5 ng	SA286: 32 ng<br>SA720: 16 ng<br>SA264: 2.5 ng<br>TTF138: 5 ng<br>GL171: 44.5 ng
SA286: 16 ng<br>SA720: 0 ng<br>SA264: 2.5 ng<br>TTF138: 5 ng<br>GL171: 76.5 ng	SA286: 16 ng<br>SA720: 1 ng<br>SA264: 2.5 ng<br>TTF138: 5 ng<br>GL171: 75.5 ng	SA286: 16 ng<br>SA720: 4 ng<br>SA264: 2.5 ng<br>TTF138: 5 ng<br>GL171: 72.5 ng	SA286: 16 ng<br>SA720: 16 ng<br>SA264: 2.5 ng<br>TTF138: 5 ng<br>GL171: 60.5 ng
SA286: 8 ng<br>SA720: 0 ng<br>SA264: 2.5 ng<br>TTF138: 5 ng<br>GL171: 84.5 ng	SA286: 8 ng<br>SA720: 1 ng<br>SA264: 2.5 ng<br>TTF138: 5 ng<br>GL171: 83.5 ng	SA286: 8 ng<br>SA720: 4 ng<br>SA264: 2.5 ng<br>TTF138: 5 ng<br>GL171: 80.5 ng	SA286: 8 ng<br>SA720: 16 ng<br>SA264: 2.5 ng<br>TTF138: 5 ng<br>GL171: 68.5 ng
SA286: 4 ng<br>SA720: 0 ng<br>SA264: 2.5 ng<br>TTF138: 5 ng<br>GL171: 88.5 ng	SA286: 4 ng<br>SA720: 1 ng<br>SA264: 2.5 ng<br>TTF138: 5 ng<br>GL171: 87.5 ng	SA286: 4 ng<br>SA720: 4 ng<br>SA264: 2.5 ng<br>TTF138: 5 ng<br>GL171: 84.5 ng	SA286: 4 ng<br>SA720: 16 ng<br>SA264: 2.5 ng<br>TTF138: 5 ng<br>GL171: 72.5 ng
SA286: 2 ng<br>SA720: 0 ng<br>SA264: 2.5 ng<br>TTF138: 5 ng<br>GL171: 90.5 ng	SA286: 2 ng<br>SA720: 1 ng<br>SA264: 2.5 ng<br>TTF138: 5 ng<br>GL171: 89.5 ng	SA286: 2 ng<br>SA720: 4 ng<br>SA264: 2.5 ng<br>TTF138: 5 ng<br>GL171: 86.5 ng	SA286: 2 ng<br>SA720: 16 ng<br>SA264: 2.5 ng<br>TTF138: 5 ng<br>GL171: 74.5 ng
SA286: 1 ng<br>SA720: 0 ng<br>SA264: 2.5 ng<br>TTF138: 5 ng<br>GL171: 91.5 ng	SA286: 1 ng<br>SA720: 1 ng<br>SA264: 2.5 ng<br>TTF138: 5 ng<br>GL171: 90.5 ng	SA286: 1 ng<br>SA720: 4 ng<br>SA264: 2.5 ng<br>TTF138: 5 ng<br>GL171: 87.5 ng	SA286: 1 ng<br>SA720: 16 ng<br>SA264: 2.5 ng<br>TTF138: 5 ng<br>GL171: 75.5 ng
SA286: 0.5 ng<br>SA720: 0 ng<br>SA264: 2.5 ng<br>TTF138: 5 ng<br>GL171: 92 ng	SA286: 0.5 ng<br>SA720: 1 ng<br>SA264: 2.5 ng<br>TTF138: 5 ng<br>GL171: 91 ng	SA286: 0.5 ng<br>SA720: 4 ng<br>SA264: 2.5 ng<br>TTF138: 5 ng<br>GL171: 88 ng	SA286: 0.5 ng<br>SA720: 16 ng<br>SA264: 2.5 ng<br>TTF138: 5 ng<br>GL171: 76 ng
SA286: 0 ng<br>SA720: 0 ng<br>SA264: 2.5 ng<br>TTF138: 5 ng<br>GL171: 92.5 ng	SA286: 0 ng<br>SA720: 1 ng<br>SA264: 2.5 ng<br>TTF138: 5 ng<br>GL171: 91.5 ng	SA286: 0 ng<br>SA720: 4 ng<br>SA264: 2.5 ng<br>TTF138: 5 ng<br>GL171: 88.5 ng	SA286: 0 ng<br>SA720: 16 ng<br>SA264: 2.5 ng<br>TTF138: 5 ng<br>GL171: 76.5 ng

Table S5. Transfection mixes used for the generalization of SensEnhance using a STAT3-responsive promoter shown in Figure 5c,f, Figure S15 and Figure S16. Amounts are given per 100 ng total DNA.

Increasing quencher (z_3) $\longrightarrow$

pMB091: 30 ng<br>SA720: 0 ng<br>SA264: 15 ng<br>TTF138: 10 ng<br>GL171: 45 ng	pMB091: 30 ng<br>SA720: 0.41 ng<br>SA264: 15 ng<br>TTF138: 10 ng<br>GL171: 44.59 ng	pMB091: 30 ng<br>SA720: 0.82 ng<br>SA264: 15 ng<br>TTF138: 10 ng<br>GL171: 44.18 ng	pMB091: 30 ng<br>SA720: 1.64 ng<br>SA264: 15 ng<br>TTF138: 10 ng<br>GL171: 43.36 ng

Table S6. Transfection mixes used to compare reporter constructs encoding 0×, 1×, and 2× IntC in IL-6-responsive SensEnhance, shown in Figure S17. Amounts are given per 100 ng total DNA.

Increasing quencher (z_3) $\longrightarrow$

**SA480**<br>0× IntC	pMB091: 30 ng<br>SA720: 0 ng<br>SA480: 15 ng<br>TTF138: 10 ng<br>GL171: 45 ng	pMB091: 30 ng<br>SA720: 0.41 ng<br>SA480: 15 ng<br>TTF138: 10 ng<br>GL171: 44.59 ng	pMB091: 30 ng<br>SA720: 0.69 ng<br>SA480: 15 ng<br>TTF138: 10 ng<br>GL171: 44.31 ng
**SA264**<br>1× IntC	pMB091: 30 ng<br>SA720: 0 ng<br>SA264: 15 ng<br>TTF138: 10 ng<br>GL171: 45 ng	pMB091: 30 ng<br>SA720: 0.41 ng<br>SA264: 15 ng<br>TTF138: 10 ng<br>GL171: 44.59 ng	pMB091: 30 ng<br>SA720: 0.69 ng<br>SA264: 15 ng<br>TTF138: 10 ng<br>GL171: 44.31 ng
**pMB124**<br>2× IntC	pMB091: 30 ng<br>SA720: 0 ng<br>pMB124: 15 ng<br>TTF138: 10 ng<br>GL171: 45 ng	pMB091: 30 ng<br>SA720: 0.41 ng<br>pMB124: 15 ng<br>TTF138: 10 ng<br>GL171: 44.59 ng	pMB091: 30 ng<br>SA720: 0.69 ng<br>pMB124: 15 ng<br>TTF138: 10 ng<br>GL171: 44.31 ng

Table S7. Transfection mixes used for IL-6-responsive IL-10 production using SensEnhance, shown in Figure S18. Amounts are given per 100 ng total DNA.

Increasing quencher (z_3) $\longrightarrow$

pMB091: 30 ng<br>SA720: 0 ng<br>pMB122: 15 ng<br>TTF138: 10 ng<br>GL171: 45 ng	pMB091: 30 ng<br>SA720: 0.41 ng<br>pMB122: 15 ng<br>TTF138: 10 ng<br>GL171: 44.59 ng	pMB091: 30 ng<br>SA720: 0.82 ng<br>pMB122: 15 ng<br>TTF138: 10 ng<br>GL171: 44.18 ng

Table S8. Hydrogel compositions (in μL) for 100 μL final volume.

Reagent	Stock concentration	Formulations						
		RGD-DTT-PEG					Enzyme-responsive	
		**5.5%**	**5.75%**	**6%**	**6.5%**	**8%**	**2.5%**	**2.75%**
TEA	0.3 M	72.9	71.7	70.6	68.4	61.7	82.8	81.1
PEG-VS	25% (w/v)	22	23	24	26	32	10	11
RGD peptide	100 mM	2.5	2.5	2.5	2.5	2.5	1	1
DTT	1 M	2.6	2.8	2.9	3.1	3.8	-	-
Peptide Q	200 mM	-	-	-	-	-	3.1	3.4
Peptide K	200 mM	-	-	-	-	-	3.1	3.4

5 Plasmid DNA Sequences

Table S9. DNA sequences for the plasmids generated in this study. Arrows indicate reverse-strand elements.

Construct	Sequence
**SA720**<br>**Backbone (20 nt)**<br>**EF1α promoter**<br>**SUMO**<br>**IntN**	GATGTCAACACAGCTACAACGGCTCCGGTGCCCGTCAGTGGGCAGAGCGCACAT<br>CGCCCACAGTCCCCGAGAAGTTGGGGGGAGGGGTCGGCAATTGAACCGGTGCCT<br>AGAGAAGGTGGCGCGGGGTAAACTGGGAAAGTGATGTCGTGTACTGGCTCCGCC<br>TTTTTCCCGAGGGTGGGGGAGAACCGTATATAAGTGCAGTAGTCGCCGTGAACG<br>TTCTTTTTCGCAACGGGTTTGCCGCCAGAACACAGGTAAGTGCCGTGTGTGGTT<br>CCCGCGGGCCTGGCCTCTTTACGGGTTATGGCCCTTGCGTGCCTTGAATTACTT<br>CCACGCCCCTGGCTGCAGTACGTGATTCTTGATCCCGAGCTTCGGGTTGGAAGT<br>GGGTGGGAGAGTTCGAGGCCTTGCGCTTAAGGAGCCCCTTCGCCTCGTGCTTGA<br>GTTGAGGCCTGGCCTGGGCGCTGGGGCCGCCGCGTGCGAATCTGGTGGCACCTT<br>CGCGCCTGTCTCGCTGCTTTCGATAAGTCTCTAGCCATTTAAAATTTTTGATGA<br>CCTGCTGCGACGCTTTTTTTCTGGCAAGATAGTCTTGTAAATGCGGGCCAAGAT<br>CTGCACACTGGTATTTCGGTTTTTGGGGCCGCGGGCGGCGACGGGGCCCGTGCG<br>TCCCAGCGCACATGTTCGGCGAGGCGGGGCCTGCGAGCGCGGCCACCGAGAATC<br>GGACGGGGGTAGTCTCAAGCTGGCCGGCCTGCTCTGGTGCCTGGCCTCGCGCCG<br>CCGTGTATCGCCCCGCCCTGGGCGGCAAGGCTGGCCCGGTCGGCACCAGTTGCG<br>TGAGCGGAAAGATGGCCGCTTCCCGGCCCTGCTGCAGGGAGCTCAAAATGGAGG<br>ACGCGGCGCTCGGGAGAGCGGGCGGGTGAGTCACCCACACAAAGGAAAAGGGCC<br>TTTCCGTCCTCAGCCGTCGCTTCATGTGACTCCACGGAGTACCGGGCGCCGTCC<br>AGGCACCTCGATTAGTTCTCGAGCTTTTGGAGTACGTCGTCTTTAGGTTGGGGG<br>GAGGGGTTTTATGCGATGGAGTTTCCCCACACTGAGTGGGTGGAGACTGAAGTT<br>AGGCCAGCTTGGCACTTGATGTAATTCTCCTTGGAATTTGCCCTTTTTGAGTTT<br>GGATCTTGGTTCATTCTCAAGCCTCAGACAGTGGTTCAAAGTTTTTTTCTTCCA<br>TTTCAGGTGTCGTGACATAAACTGCCCTAGACCACCTATGGGAAGCGGGTTGGT<br>GCCCAGAGGAAGCGCATCTATGTCTGACAGTGAGGTTAACCAAGAAGCCAAGCC<br>CGAAGTTAAGCCTGAGGTTAAACCCGAAACTCATATAAATCTGAAAGTTTCCGA<br>TGGCTCTTCAGAAATATTCTTTAAAATTAAGAAAACCACTCCTTTGCGCCGGCT<br>TATGGAAGCATTCGCGAAAAGACAGGGCAAAGAGATGGATTCCCTGAGGTTCTT<br>GTATGATGGGATCAGAATCCAAGCTGACCAGACACCAGAGGATCTTGACATGGA<br>AGATAACGACATAATCGAAGCTCATCGGGAACAGATTGGTGGGACACGCAGCGG<br>TTATTGTCTCGATTTGAAAACGCAAGTGCAAACACCACAAGGTATGAAAGAAAT<br>AAGCAATATTCAAGTCGGGGACCTGGTCCTTTCCAACACTGGGTACAATGAAGT<br>GCTGAATGTGTTTCCTAAAAGTAAGAAGAAAAGTTATAAGATCACACTGGAAGA<br>TGGAAAAGAGATAATTTGCTCAGAGGAGCACCTGTTCCCGACACAAACCGGCGA<br>GATGAACATCAGCGGCGGCCTGAAGGAGGGCATGTGCCTGTACGTGAAGGAGGG<br>TTCTGGCGGTGGTGGTAGTGGTGGAGGTGGATCTGGAGGTGGTGGCTCAGGATC<br>CTAGGCTAAGTAGTAATCTAGAGG

Construct	Sequence
**pMB007**<br>**Backbone**<br>**LMNA promoter**<br>**Firefly luciferase**	AGGTGCCAGAACATTTCTCTATCGATAGGTACCGAGCTCTTACGCGTGCTAGGT<br>AGAAAGGGACTGAAGGGGGAAGGAACCCAGGCAGTGCTTGGGTCCAAAGGAAGG<br>GGACAGGAGATGGAAGGGGCAGTGCCTGGCTCCTATTCTTGGCTTTCTTTAGGG<br>GACTTCTTTAGGGGACTGTGGCTTGTTGCTTGGGTCTAAAAACGAATGCTTGGC<br>TTTGAAGAGAGATAGATTGGGGCAAAAGAAAGAAAAAAAGGGACCCCCCAAACT<br>CCTTGATCCCTGGCCCCAAACTGGGGGCATAAAGGAACTCAGGTTCCAGAACTT<br>TGCTCCCCCCAGGGAACCCAGGCATTCCTTCTCCACCCCACTCCTGGCACACTG<br>AGATGCAGCTCTGAATGGGCTGCCCACGTGTGGAGGGGGGTTGGGGTGACTCAC<br>TATTACTACTGGGAGGACAGGGGGAGCCAGTGGTGGAAGAAGGGTGAGTCACAC<br>TGATGGGCACCAGCCTCAGCCCTCCCCCCACTTTCCTGGCTCCCAGCCCTGCCT<br>ACCTGACCCTCTCCCTTGCTTTGCGCCCACTTCCCTCTCTTTCTCCCCGACCCT<br>TTTGCCCACCCACTCTCCCTCCTTGGCTCTGCCCTCTAGCCCAGAAGGTCTGAG<br>GCAATGGGGGCAAGCTTGGAGCCGACAGTGCTGAGCAGGCAGGAGCCAAGAGAG<br>GGGAAGCTTGAGCCTCACGCAGTTAGGGGTGCGCTGGAGAGGGTGGGGCCCGAC<br>TCCGCCACACCCCAACGGTCCTTCCCCCTCCTCACCACTCCCGCCCCCACCCCC<br>AATGGATCTGGGACTGCCCCTTTAAGAGTAGTGGCCCCTCCTCCCTTCAGAGGA<br>GGACCTATTAGAGCCTTTGCCCCGGCGTCGGTGACTCAGTGTTCGCGGGAGCGC<br>CGCACCTACACCAGCCAACCCAGATCCCGAGGTCCGACAGCGCCCGGCCCAGAT<br>CCCCACGCCTGCCAGGAGCAAGCCGAGAGCCAGCCGGCCGGCGCACTCCGACTC<br>CGAGCAGTCTCTGTCCTTCGACCCGAGCCCCGCGCCCTTTCCGGGACCCCTGCC<br>CCGCGGGCAGCGCTGCCAACCTGCCACCATGGAAGACGCCAAAAACATAAAGAA<br>AGGCCCGGCGCCATTCTATCCGCTGGAAGATGGAACCGCTGGAGAGCAACTGCA<br>TAAGGCTATGAAGAGATACGCCCTGGTTCCTGGAACAATTGCTTTTACAGATGC<br>ACATATCGAGGTGGACATCACTTACGCTGAGTACTTCGAAATGTCCGTTCGGTT<br>GGCAGAAGCTATGAAACGATATGGGCTGAATACAAATCACAGAATCGTCGTATG<br>CAGTGAAAACTCTCTTCAATTCTTTATGCCGGTGTTGGGCGCGTTATTTATCGG<br>AGTTGCAGTTGCGCCCGCGAACGACATTTATAATGAACGTGAATTGCTCAACAG<br>TATGGGCATTTCGCAGCCTACCGTGGTGTTCGTTTCCAAAAAGGGGTTGCAAAA<br>AATTTTGAACGTGCAAAAAAAGCTCCCAATCATCCAAAAAATTATTATCATGGA<br>TTCTAAAACGGATTACCAGGGATTTCAGTCGATGTACACGTTCGTCACATCTCA<br>TCTACCTCCCGGTTTTAATGAATACGATTTTGTGCCAGAGTCCTTCGATAGGGA<br>CAAGACAATTGCACTGATCATGAACTCCTCTGGATCTACTGGTCTGCCTAAAGG<br>TGTCGCTCTGCCTCATAGAACTGCCTGCGTGAGATTCTCGCATGCCAGAGATCC<br>TATTTTTGGCAATCAAATCATTCCGGATACTGCGATTTTAAGTGTTGTTCCATT<br>CCATCACGGTTTTGGAATGTTTACTACACTCGGATATTTGATATGTGGATTTCG<br>AGTCGTCTTAATGTATAGATTTGAAGAAGAGCTGTTTCTGAGGAGCCTTCAGGA<br>TTACAAGATTCAAAGTGCGCTGCTGGTGCCAACCCTATTCTCCTTCTTCGCCAA<br>AAGCACTCTGATTGACAAATACGATTTATCTAATTTACACGAAATTGCTTCTGG<br>TGGCGCTCCCCTCTCTAAGGAAGTCGGGGAAGCGGTTGCCAAGAGGTTCCATCT<br>GCCAGGTATCAGGCAAGGATATGGGCTCACTGAGACTACATCAGCTATTCTGAT<br>TACACCCGAGGGGGATGATAAACCGGGCGCGGTCGGTAAAGTTGTTCCATTTTT<br>TGAAGCGAAGGTTGTGGATCTGGATACCGGGAAAACGCTGGGCGTTAATCAAAG<br>AGGCGAACTGTGTGTGAGAGGTCCTATGATTATGTCCGGTTATGTAAACAATCC<br>GGAAGCGACCAACGCCTTGATTGACAAGGATGGATGGCTACATTCTGGAGACAT<br>AGCTTACTGGGACGAAGACGAACACTTCTTCATCGTTGACCGCCTGAAGTCTCT<br>GATTAAGTACAAAGGCTATCAGGTGGCTCCCGCTGAATTGGAATCCATCTTGCT<br>CCAACACCCCAACATCTTCGACGCAGGTGTCGCAGGTCTTCCCGACGATGACGC<br>CGGTGAACTTCCCGCCGCCGTTGTTGTTTTGGAGCACGGAAAGACGATGACGGA<br>AAAAGAGATCGTGGATTACGTCGCCAGTCAAGTAACAACCGCGAAAAAGTTGCG<br>CGGAGGAGTTGTGTTTGTGGACGAAGTACCGAAAGGTCTTACCGGAAAACTCGA<br>CGCAAGAAAAATCAGAGAGATCCTCATAAAGGCCAAGAAGGGCGGAAAGATCGC<br>CGTGTAATTCTAGAGTCGGGGCGGCCG

Construct	Sequence
**pMB025k**<br>**Backbone (20 nt)**<br>**5×UAS**<br>**MinP**<br>**IntC**<br>**P2A**<br>**T2A**<br>**d2EYFP**	ATAGGTACCGAGTTTCTAGACGGAGTACTGTCCTCCGAGCGGAGTACTGTCCTC<br>CGACTCGAGCGGAGTACTGTCCTCCGATCGGAGTACTGTCCTCCGCGAATTCCG<br>GAGTACTGTCCTCCGAAGACAAGCTTAGACACTAGAGGGTATATAATGGAAGCT<br>CGACTTCCAGCTTGGCAATCCGGTACTGTTGGTAAAGCCACCATGATGCTGAAG<br>AAAATCCTTAAAATAGAGGAACTTGACGAACGGGAACTGATCGACATTGAGGTT<br>TCCGGGAATCATTTATTCTATGCAAATGATATCTTAACCCATAATAGTGGGAGC<br>GGTTCTGGAAGCGGAGCGACCAACTTTAGCCTGCTGAAACAGGCGGGCGATGTG<br>GAAGAAAACCCTGGACCTGGAAGCGGAGAGGGCAGAGGAAGTCTGCTAACATGC<br>GGTGACGTCGAGGAGAATCCTGGCCCAATCGACGGTGGTTCCGGAGGATCCGCC<br>ACCATGGTGAGCAAGGGCGAGGAGCTGTTCACCGGGGTGGTGCCCATCCTGGTC<br>GAGCTGGACGGCGACGTAAACGGCCACAAGTTCAGCGTGTCCGGCGAGGGCGAG<br>GGCGATGCCACCTACGGCAAGCTGACCCTGAAGTTCATCTGCACCACCGGCAAG<br>CTGCCCGTGCCCTGGCCCACCCTCGTGACCACCTTCGGCTACGGCCTGCAGTGC<br>TTCGCCCGCTACCCCGACCACATGAAGCAGCACGACTTCTTCAAGTCCGCCATG<br>CCCGAAGGCTACGTCCAGGAGCGCACCATCTTCTTCAAGGACGACGGCAACTAC<br>AAGACCCGCGCCGAGGTGAAGTTCGAGGGCGACACCCTGGTGAACCGCATCGAG<br>CTGAAGGGCATCGACTTCAAGGAGGACGGCAACATCCTGGGGCACAAGCTGGAG<br>TACAACTACAACAGCCACAACGTCTATATCATGGCCGACAAGCAGAAGAACGGC<br>ATCAAGGTGAACTTCAAGATCCGCCACAACATCGAGGACGGCAGCGTGCAGCTC<br>GCCGACCACTACCAGCAGAACACCCCCATCGGCGACGGCCCCGTGCTGCTGCCC<br>GACAACCACTACCTGAGCTACCAGTCCGCCCTGAGCAAAGACCCCAACGAGAAG<br>CGCGATCACATGGTCCTGCTGGAGTTCGTGACCGCCGCCGGGATCACTCTCGGC<br>ATGGACGAGCTGTACAAGAAGCTTAGCCATGGCTTCCCGCCGGAGGTGGAGGAG<br>CAGGATGATGGCACGCTGCCCATGTCTTGTGCCCAGGAGAGCGGGATGGACCGT<br>CACCCTGCAGCCTGTGCTTCTGCTAGGATCAATGTGTAGATGCGCGGCCGCTTA<br>AGCTA

Construct	Sequence
**pMB029**<br>Backbone (20 nt)<br>Tet-On 3G ←<br>hPGK promoter ←<br>TRE3GS promoter<br>SUMO<br>IntN<br>P2A<br>T2A<br>mCherry	ACTGACACACATTCGCGCGTTTACCCGGGGAGCATGTCAAGGTCAAAATCGTCA<br>AGAGCGTCAGCAGGCAGCATATCAAGGTCAAAGTCGTCAAGGGCATCGGCTGGG<br>AGCATGTCTAAGTCAAAATCGTCAAGGGCGTCGGTCGGCCCGCCGCTTTCGCAC<br>TTTAGCTGTTTCTCCAGGCCACATATGATTAGTTCCAGGCCGAAAAGGAAGGCA<br>GGTTCGGCTCCCTGCCGGTCGAACAGCTCAATTGCTTGTTTCAGAAGTGGGGGC<br>ATAGAATCGGTGGTAGGTGTCTCTCTTTCCTCTTTTGCTACTTGATGCTCCTGT<br>TCCTCCAATACGCAGCCCAGTGTAAAGTGGCCCACGGCGGACAGAGCGTACAGT<br>GCGTTCTCCAGGGAGAAGCCTTGCTGACACAGGAACGCGAGCTGATTTTCCAGG<br>GTTTCGTACTGTTTCTCTGTTGGGCGGGTGCCGAGATGCACTTTAGCCCCGTCG<br>CGATGTGAGAGGAGAGCACAGCGGTATGACTTGGCGTTGTTCCGCAGAAAGTCT<br>TGCCATGACTCGCCTTCCAGGGGGCAGGAGTGGGTATGATGCCTGTCCAGCATC<br>TCGATTGGCAGGGCATCGAGCAGGGCCCGCTTGTTCTTCACGTGCCAGTACAGG<br>GTAGGCTGCTCAACTCCCAGCTTTTGAGCGAGTTTCCTTGTCGTCAGGCCTTCG<br>ATACCGACTCCATTGAGTAATTCCAGAGCAGAGTTTATGACTTTGCTCTTGTCC<br>AGTCTAGACATGGTAATTCGATGATCCCCCTGGGGAGAGAGGTCGGTGATTCGG<br>TCAACGAGGGAGCCGACTGCCGACGTGCGCTCCGGAGGCTTGCAGAATGCGGAA<br>CACCGCGCGGGCAGGAACAGGGCCCACACTACCGCCCCACACCCCGCCTCCCGC<br>ACCGCCCCTTCCCGGCCGCTGCTCTCGGCGCGCCCTGCTGAGCAGCCGCTATTG<br>GCCACAGCCCATCGCGGTCGGCGCGCTGCCATTGCTCCCTGGCGCTGTCCGTCT<br>GCGAGGGTACTAGTGAGACGTGCGGCTTCCGTTTGTCACGTCCGGCACGCCGCG<br>AACCGCAAGGAACCTTCCCGACTTAGGGGCGGAGCAGGAAGCGTCGCCGGGGGG<br>CCCACAAGGGTAGCGGCGAAGATCCGGGTGACGCTGCGAACGGACGTGAAGAAT<br>GTGCGAGACCCAGGGTCGGCGCCGCTGCGTTTCCCGGAACCACGCCCAGAGCAG<br>CCGCGTCCCTGCGCAAACCCAGGGCTGCCTTGGAAAAGGCGCAACCCCAACCCC<br>GTGGAATTATCACCTCGAGTTTACTCCCTATCAGTGATAGAGAACGTATGAAGA<br>GTTTACTCCCTATCAGTGATAGAGAACGTATGCAGACTTTACTCCCTATCAGTG<br>ATAGAGAACGTATAAGGAGTTTACTCCCTATCAGTGATAGAGAACGTATGACCA<br>GTTTACTCCCTATCAGTGATAGAGAACGTATCTACAGTTTACTCCCTATCAGTG<br>ATAGAGAACGTATATCCAGTTTACTCCCTATCAGTGATAGAGAACGTATAAGCT<br>TTGCTTATGTAAACCAGGGCGCCTATAAAAGAGTGCTGATTTTTTGAGTAAACT<br>TCAATTCCACAACACTTTTGTCTTATACCAACTTTCCGTACCACTTCCTACCCT<br>CGTAAAGGCCACCATGGGAAGCGGGTTGGTGCCCAGAGGAAGCGCATCTATGTC<br>TGACAGTGAGGTTAACCAAGAAGCCAAGCCCGAAGTTAAGCCTGAGGTTAAACC<br>CGAAACTCATATAAATCTGAAAGTTTCCGATGGCTCTTCAGAAATATTCTTTAA<br>AATTAAGAAAACCACTCCTTTGCGCCGGCTTATGGAAGCATTCGCGAAAAGACA<br>GGGCAAAGAGATGGATTCCCTGAGGTTCTTGTATGATGGGATCAGAATCCAAGC<br>TGACCAGACACCAGAGGATCTTGACATGGAAGATAACGACATAATCGAAGCTCA<br>TCGGGAACAGATTGGTGGGACACGCAGCGGTTATTGTCTCGATTTGAAAACGCA<br>AGTGCAAACACCACAAGGTATGAAAGAAATAAGCAATATTCAAGTCGGGGACCT<br>GGTCCTTTCCAACACTGGGTACAATGAAGTGCTGAATGTGTTTCCTAAAAGTAA<br>GAAGAAAAGTTATAAGATCACACTGGAAGATGGAAAAGAGATAATTTGCTCAGA<br>GGAGCACCTGTTCCCGACACAAACCGGCGAGATGAACATCAGCGGCGGCCTGAA<br>GGAGGGCATGTGCCTGTACGTGAAGGAGGGTTCTGGCGGTGGTGGTAGTGGTGG<br>AGGTGGATCTGGAGGTGGTGGCTCAGGATCCGGTTCTGGAAGCGGAGCGACCAA<br>CTTTAGCCTGCTGAAACAGGCGGGCGATGTGGAAGAAAACCCTGGACCTGGAAG<br>CGGAGAGGGCAGAGGAAGTCTGCTAACATGCGGTGACGTCGAGGAGAATCCTGG<br>CCCAATCGACGGTGGTTCCGGAGGATCCGCCACCATGGTGAGCAAGGGCGAGGA<br>GGATAACATGGCCATCATCAAGGAGTTCATGCGCTTCAAGGTGCACATGGAGGG<br>CTCCGTGAACGGCCACGAGTTCGAGATCGAGGGCGAGGGCGAGGGCCGCCCCTA<br>CGAGGGCACCCAGACCGCCAAGCTGAAGGTGACCAAGGGTGGCCCCCTGCCCTT<br>CGCCTGGGACATCCTGTCCCCTCAGTTCATGTACGGCTCCAAGGCCTACGTGAA<br>GCACCCCGCCGACATCCCCGACTACTTGAAGCTGTCCTTCCCCGAGGGCTTCAA<br>GTGGGAGCGCGTGATGAACTTCGAGGACGGCGGCGTGGTGACCGTGACCCAGGA<br>CTCCTCCCTGCAGGACGGCGAGTTCATCTACAAGGTGAAGCTGCGCGGCACCAA<br>CTTCCCCTCCGACGGCCCCGTAATGCAGAAGAAGACCATGGGCTGGGAGGCCTC<br>CTCCGAGCGGATGTACCCCGAGGACGGCGCCCTGAAGGGCGAGATCAAGCAGAG<br>GCTGAAGCTGAAGGACGGCGGCCACTACGACGCTGAGGTCAAGACCACCTACAA<br>GGCCAAGAAGCCCGTGCAGCTGCCCGGCGCCTACAACGTCAACATCAAGTTGGA<br>CATCACCTCCCACAACGAGGACTACACCATCGTGGAACAGTACGAACGCGCCGA<br>GGGCCGCCACTCCACCGGCGGCATGGACGAGCTGTACAAGTGATGACAGACCAC<br>CTCCCCTGC

Construct	Sequence
**pMB042**<br>**Backbone (20 nt)**<br>**8×GTIIC promoter**<br>**GTIIC motifs**<br>**Gal4 (1–147)**<br>**IntC**<br>**VP64**<br>**SV40 NLS**<br>**RelA (p65)**<br>**Rta AD**<br>**P2A**<br>**T2A**<br>**TagBFP**	TAACAACGAGCTCTTACGCGTGCTAGCCCGGCCAGTGCCAAGTTGAGACACATT<br>CCACACATTCCACTGCAAGCTTGAGACACATTCCACACATTCCACTGCAAGCTT<br>CTAGCCCGGCCAGTGCCAAGTTGAGACACATTCCACACATTCCACTGCAAGCTT<br>GAGACACATTCCACACATTCCACTGCAAGCTTCTAGAGATCTGCAGGTCGAGGT<br>CGACGGTATCGATAAGCTTGGGGGTGGGCGCCGGGGGGACCTTAAAGCCTCTGC<br>CCCCCAAGGAGCCCTTCCCAGACAGCCGCCGGCACCCACCGCTCCGTGGGACGA<br>TCCCCCAAGAGCTTGGCATTCCGGTACTGTTGGTAAAGCCACCATGAAACTGCT<br>TAGCAGCATCGAGCAGGCCTGTGACATCTGTCGGCTCAAGAAACTGAAATGTAG<br>CAAGGAGAAGCCTAAATGTGCAAAATGCCTCAAGAATAATTGGGAATGCCGGTA<br>TAGCCCTAAGACTAAGAGAAGTCCTTTGACACGGGCCCACCTTACCGAGGTCGA<br>GTCCAGACTTGAGCGCCTCGAGGGTTCTATGCTGAAGAAAATCCTTAAAATAGA<br>GGAACTTGACGAACGGGAACTGATCGACATTGAGGTTTCCGGGAATCATTTATT<br>CTATGCAAATGATATCTTAACCCATAATAGTGGGAGCGGATCCCAATTGTTCTT<br>GCTTATCTTCCCACGCGAGGATCTGGATATGATCCTTAAGATGGACTCACTGCA<br>AGACATCAAGGCCCTTCTTACCGGCCTCTTCGTTCAGGACAACGTCAACAAGGA<br>CGCAGTGACGGACAGGCTGGCCAGCGTAGAAACCGACATGCCCTTGACTCTGCG<br>GCAACACCGCATCTCCGCAACGAGCAGTTCCGAGGAATCTTCCAATAAGGGACA<br>GCGGCAACTCACAGTGAGCGGTTCTTCCGAGGCCAGCGGTTCCGGACGGGCTGA<br>CGCATTGGACGATTTTGATCTGGATATGCTGGGAAGTGACGCCCTCGATGATTT<br>TGACCTTGACATGCTTGGTTCGGATGCCCTTGATGACTTTGACCTCGACATGCT<br>CGGCAGTGACGCCCTTGATGATTTCGACCTGGACATGCTGATTAACTCTAGAAG<br>TTCCGGATCTCCGAAAAAGAAACGCAAAGTTGGTAGCCAGTACCTGCCCGACAC<br>CGACGACCGGCACCGGATCGAGGAAAAGCGGAAGCGGACCTACGAGACATTCAA<br>GAGCATCATGAAGAAGTCCCCCTTCAGCGGCCCCACCGACCCTAGACCTCCACC<br>TAGAAGAATCGCCGTGCCCAGCAGATCCAGCGCCAGCGTGCCAAAACCTGCCCC<br>CCAGCCTTACCCCTTCACCAGCAGCCTGAGCACCATCAACTACGACGAGTTCCC<br>TACCATGGTGTTCCCCAGCGGCCAGATCTCTCAGGCCTCTGCTCTGGCTCCAGC<br>CCCTCCTCAGGTGCTGCCTCAGGCTCCTGCTCCTGCACCAGCTCCAGCCATGGT<br>GTCTGCACTGGCTCAGGCACCAGCACCCGTGCCTGTGCTGGCTCCTGGACCTCC<br>ACAGGCTGTGGCTCCACCAGCCCCTAAACCTACACAGGCCGGCGAGGGCACACT<br>GTCTGAAGCTCTGCTGCAGCTGCAGTTCGACGACGAGGATCTGGGAGCCCTGCT<br>GGGAAACAGCACCGATCCTGCCGTGTTCACCGACCTGGCCAGCGTGGACAACAG<br>CGAGTTCCAGCAGCTGCTGAACCAGGGCATCCCTGTGGCCCCTCACACCACCGA<br>GCCCATGCTGATGGAATACCCCGAGGCCATCACCCGGCTCGTGACAGGCGCTCA<br>GAGGCCTCCTGATCCAGCTCCTGCCCCTCTGGGAGCACCAGGCCTGCCTAATGG<br>ACTGCTGTCTGGCGACGAGGACTTCAGCTCTATCGCCGATATGGATTTCTCAGC<br>CTTGCTGGGCTCTGGCAGCGGCAGCCGGGATTCCAGGGAAGGGATGTTTTTGCC<br>GAAGCCTGAGGCCGGCTCCGCTATTAGTGACGTGTTTGAGGGCCGCGAGGTGTG<br>CCAGCCAAAACGAATCCGGCCATTTCATCCTCCAGGAAGTCCATGGGCCAACCG<br>CCCACTCCCCGCCAGCCTCGCACCAACACCAACCGGTCCAGTACATGAGCCAGT<br>CGGGTCACTGACCCCGGCACCAGTCCCTCAGCCACTGGATCCAGCGCCCGCAGT<br>GACTCCCGAGGCCAGTCACCTGTTGGAGGATCCCGATGAAGAGACTAGCCAGGC<br>TGTCAAAGCCCTTCGGGAGATGGCCGATACTGTGATTCCCCAGAAGGAAGAGGC<br>TGCAATCTGTGGCCAAATGGACCTTTCCCATCCGCCCCCAAGGGGCCATCTGGA<br>TGAGCTGACAACCACACTTGAGTCCATGACCGAGGATCTGAACCTGGACTCACC<br>CCTGACCCCGGAATTGAACGAGATTCTGGATACCTTCCTGAACGACGAGTGCCT<br>CTTGCATGCCATGCATATCAGCACAGGACTGTCCATCTTCGACACATCTCTGTT<br>TGGATCCGGTTCTGGAAGCGGAGCGACCAACTTTAGCCTGCTGAAACAGGCGGG<br>CGATGTGGAAGAAAACCCTGGACCTGGAAGCGGAGAGGGCAGAGGAAGTCTGCT<br>AACATGCGGTGACGTCGAGGAGAATCCTGGCCCAATCGACGGTGGTTCCGGAGG<br>ATCCGCCACCATGAGCGAGCTGATTAAGGAGAACATGCACATGAAGCTGTACAT<br>GGAGGGCACCGTGGACAACCATCACTTCAAGTGCACATCCGAGGGCGAAGGCAA<br>GCCCTACGAGGGCACCCAGACCATGAGAATCAAGGTGGTCGAGGGCGGCCCTCT<br>CCCCTTCGCCTTCGACATCCTGGCTACTAGCTTCCTCTACGGCAGCAAGACCTT<br>CATCAACCACACCCAGGGCATCCCCGACTTCTTCAAGCAGTCCTTCCCTGAGGG<br>CTTCACATGGGAGAGAGTCACCACATACGAAGACGGGGGCGTGCTGACCGCTAC<br>CCAGGACACCAGCCTCCAGGACGGCTGCCTCATCTACAACGTCAAGATCAGAGG<br>GGTGAACTTCACATCCAACGGCCCTGTGATGCAGAAGAAAACACTCGGCTGGGA<br>GGCCTTCACCGAGACGCTGTACCCCGCTGACGGCGGCCTGGAAGGCAGAAACGA<br>CATGGCCCTGAAGCTCGTGGGCGGGAGCCATCTGATCGCAAACATCAAGACCAC<br>ATATAGATCCAAGAAACCCGCTAAGAACCTCAAGATGCCTGGCGTCTACTATGT<br>GGACTACAGACTGGAAAGAATCAAGGAGGCCAACAACGAGACCTACGTCGAGCA<br>GCACGAGGTGGCAGTGGCCAGATACTGCGACCTCCCTAGCAAACTGGGGCACAA<br>GCTTAATTGACCGGCCGCTTCATAAGTCGA

Construct	Sequence
**pMB091**<br>Backbone (20 nt)<br>3×SIE<br>Minimal CMV promoter<br>Gal4 (1–147)<br>IntC<br>VP64<br>SV40 NLS<br>RelA (p65)<br>Rta AD<br>P2A<br>T2A<br>TagBFP	GCCATGCATTAGTTATTAATGTCGACATTTCCCGTAAATCGTCGAGTCGACATT<br>TCCCGTAAATCGTCGAGTCGACATTTCCCGTAAATCGTCGAACGCGTTAGGCGT<br>GTACGGTGGGAGGTCTATATAAGCAGAGCTGGTTTAGTGAACCGTCAGATCCGC<br>TAGCGCTACCGGACTCAGATCTCGAGCTCAAGCTTCGAATTCTGCAGTCGACGG<br>TACCGCGGGCCCGGGATCCACCGGTCGCCACCATGAAACTGCTTAGCAGCATCG<br>AGCAGGCCTGTGACATCTGTCGGCTCAAGAAACTGAAATGTAGCAAGGAGAAGC<br>CTAAATGTGCAAAATGCCTCAAGAATAATTGGGAATGCCGGTATAGCCCTAAGA<br>CTAAGAGAAGTCCTTTGACACGGGCCCACCTTACCGAGGTCGAGTCCAGACTTG<br>AGCGCCTCGAGGGTTCTATGCTGAAGAAAATCCTTAAAATAGAGGAACTTGACG<br>AACGGGAACTGATCGACATTGAGGTTTCCGGGAATCATTTATTCTATGCAAATG<br>ATATCTTAACCCATAATAGTGGGAGCGGATCCCAATTGTTCTTGCTTATCTTCC<br>CACGCGAGGATCTGGATATGATCCTTAAGATGGACTCACTGCAAGACATCAAGG<br>CCCTTCTTACCGGCCTCTTCGTTCAGGACAACGTCAACAAGGACGCAGTGACGG<br>ACAGGCTGGCCAGCGTAGAAACCGACATGCCCTTGACTCTGCGGCAACACCGCA<br>TCTCCGCAACGAGCAGTTCCGAGGAATCTTCCAATAAGGGACAGCGGCAACTCA<br>CAGTGAGCGGTTCTTCCGAGGCCAGCGGTTCCGGACGGGCTGACGCATTGGACG<br>ATTTTGATCTGGATATGCTGGGAAGTGACGCCCTCGATGATTTTGACCTTGACA<br>TGCTTGGTTCGGATGCCCTTGATGACTTTGACCTCGACATGCTCGGCAGTGACG<br>CCCTTGATGATTTCGACCTGGACATGCTGATTAACTCTAGAAGTTCCGGATCTC<br>CGAAAAAGAAACGCAAAGTTGGTAGCCAGTACCTGCCCGACACCGACGACCGGC<br>ACCGGATCGAGGAAAAGCGGAAGCGGACCTACGAGACATTCAAGAGCATCATGA<br>AGAAGTCCCCCTTCAGCGGCCCCACCGACCCTAGACCTCCACCTAGAAGAATCG<br>CCGTGCCCAGCAGATCCAGCGCCAGCGTGCCAAAACCTGCCCCCCAGCCTTACC<br>CCTTCACCAGCAGCCTGAGCACCATCAACTACGACGAGTTCCCTACCATGGTGT<br>TCCCCAGCGGCCAGATCTCTCAGGCCTCTGCTCTGGCTCCAGCCCCTCCTCAGG<br>TGCTGCCTCAGGCTCCTGCTCCTGCACCAGCTCCAGCCATGGTGTCTGCACTGG<br>CTCAGGCACCAGCACCCGTGCCTGTGCTGGCTCCTGGACCTCCACAGGCTGTGG<br>CTCCACCAGCCCCTAAACCTACACAGGCCGGCGAGGGCACACTGTCTGAAGCTC<br>TGCTGCAGCTGCAGTTCGACGACGAGGATCTGGGAGCCCTGCTGGGAAACAGCA<br>CCGATCCTGCCGTGTTCACCGACCTGGCCAGCGTGGACAACAGCGAGTTCCAGC<br>AGCTGCTGAACCAGGGCATCCCTGTGGCCCCTCACACCACCGAGCCCATGCTGA<br>TGGAATACCCCGAGGCCATCACCCGGCTCGTGACAGGCGCTCAGAGGCCTCCTG<br>ATCCAGCTCCTGCCCCTCTGGGAGCACCAGGCCTGCCTAATGGACTGCTGTCTG<br>GCGACGAGGACTTCAGCTCTATCGCCGATATGGATTTCTCAGCCTTGCTGGGCT<br>CTGGCAGCGGCAGCCGGGATTCCAGGGAAGGGATGTTTTTGCCGAAGCCTGAGG<br>CCGGCTCCGCTATTAGTGACGTGTTTGAGGGCCGCGAGGTGTGCCAGCCAAAAC<br>GAATCCGGCCATTTCATCCTCCAGGAAGTCCATGGGCCAACCGCCCACTCCCCG<br>CCAGCCTCGCACCAACACCAACCGGTCCAGTACATGAGCCAGTCGGGTCACTGA<br>CCCCGGCACCAGTCCCTCAGCCACTGGATCCAGCGCCCGCAGTGACTCCCGAGG<br>CCAGTCACCTGTTGGAGGATCCCGATGAAGAGACTAGCCAGGCTGTCAAAGCCC<br>TTCGGGAGATGGCCGATACTGTGATTCCCCAGAAGGAAGAGGCTGCAATCTGTG<br>GCCAAATGGACCTTTCCCATCCGCCCCCAAGGGGCCATCTGGATGAGCTGACAA<br>CCACACTTGAGTCCATGACCGAGGATCTGAACCTGGACTCACCCCTGACCCCGG<br>AATTGAACGAGATTCTGGATACCTTCCTGAACGACGAGTGCCTCTTGCATGCCA<br>TGCATATCAGCACAGGACTGTCCATCTTCGACACATCTCTGTTTGGATCCGGTT<br>CTGGAAGCGGAGCGACCAACTTTAGCCTGCTGAAACAGGCGGGCGATGTGGAAG<br>AAAACCCTGGACCTGGAAGCGGAGAGGGCAGAGGAAGTCTGCTAACATGCGGTG<br>ACGTCGAGGAGAATCCTGGCCCAATCGACGGTGGTTCCGGAGGATCCGCCACCA<br>TGAGCGAGCTGATTAAGGAGAACATGCACATGAAGCTGTACATGGAGGGCACCG<br>TGGACAACCATCACTTCAAGTGCACATCCGAGGGCGAAGGCAAGCCCTACGAGG<br>GCACCCAGACCATGAGAATCAAGGTGGTCGAGGGCGGCCCTCTCCCCTTCGCCT<br>TCGACATCCTGGCTACTAGCTTCCTCTACGGCAGCAAGACCTTCATCAACCACA<br>CCCAGGGCATCCCCGACTTCTTCAAGCAGTCCTTCCCTGAGGGCTTCACATGGG<br>AGAGAGTCACCACATACGAAGACGGGGGCGTGCTGACCGCTACCCAGGACACCA<br>GCCTCCAGGACGGCTGCCTCATCTACAACGTCAAGATCAGAGGGGTGAACTTCA<br>CATCCAACGGCCCTGTGATGCAGAAGAAAACACTCGGCTGGGAGGCCTTCACCG<br>AGACGCTGTACCCCGCTGACGGCGGCCTGGAAGGCAGAAACGACATGGCCCTGA<br>AGCTCGTGGGCGGGAGCCATCTGATCGCAAACATCAAGACCACATATAGATCCA<br>AGAAACCCGCTAAGAACCTCAAGATGCCTGGCGTCTACTATGTGGACTACAGAC<br>TGGAAAGAATCAAGGAGGCCAACAACGAGACCTACGTCGAGCAGCACGAGGTGG<br>CAGTGGCCAGATACTGCGACCTCCCTAGCAAACTGGGGCACAAGCTTAATTGAC<br>CGGCCGCTTCATAAGTCGA

Construct	Sequence
**pMB122**<br>**Backbone (20 nt)**<br>**5×UAS**<br>**MinP**<br>**IntC**<br>**P2A**<br>**T2A**<br>**hIL-10**	ATAGGTACCGAGTTTCTAGACGGAGTACTGTCCTCCGAGCGGAGTACTGTCCTC<br>CGACTCGAGCGGAGTACTGTCCTCCGATCGGAGTACTGTCCTCCGCGAATTCCG<br>GAGTACTGTCCTCCGAAGACAAGCTTAGACACTAGAGGGTATATAATGGAAGCT<br>CGACTTCCAGCTTGGCAATCCGGTACTGTTGGTAAAGCCACTATGATGCTGAAG<br>AAAATCCTTAAAATAGAGGAACTTGACGAACGGGAACTGATCGACATTGAGGTT<br>TCCGGGAATCATTTATTCTATGCAAATGATATCTTAACCCATAATAGTGGGAGC<br>GGTTCTGGAAGCGGAGCGACCAACTTTAGCCTGCTGAAACAGGCGGGCGATGTG<br>GAAGAAAACCCTGGACCTGGAAGCGGAGAGGGCAGAGGAAGTCTGCTAACATGC<br>GGTGACGTCGAGGAGAATCCTGGCCCAATGCATAGCTCTGCCCTGCTGTGCTGT<br>CTGGTCCTCTTGACAGGAGTGAGGGCTTCACCTGGACAAGGAACCCAGTCTGAA<br>AACAGCTGCACTCACTTTCCCGGTAATCTCCCCAATATGCTGCGGGATTTGAGG<br>GACGCCTTTAGTAGGGTGAAGACGTTCTTTCAGATGAAAGACCAACTCGATAAC<br>TTGCTCCTGAAAGAGTCACTCTTGGAGGATTTTAAGGGTTATCTTGGTTGCCAG<br>GCATTGAGTGAGATGATTCAGTTCTATTTGGAAGAAGTAATGCCTCAGGCTGAG<br>AACCAGGATCCCGACATTAAAGCGCACGTAAATTCCCTCGGTGAAAATTTGAAG<br>ACACTCAGGCTCCGGCTGCGACGCTGCCACCGCTTTCTTCCGTGCGAAAATAAG<br>TCTAAAGCCGTGGAGCAGGTGAAGAATGCTTTCAATAAGCTCCAAGAGAAAGGC<br>ATATATAAAGCAATGTCAGAGTTTGACATTTTCATCAACTACATCGAAGCCTAC<br>ATGACCATGAAAATCAGAAATTAGTAACGCTTCGAGCAGACATG
**pMB124**<br>**Backbone (20 nt)**<br>**5×UAS**<br>**MinP**<br>**IntC (×2)**<br>**P2A**<br>**T2A**<br>**mVenus**	ATAGGTACCGAGTTTCTAGACGGAGTACTGTCCTCCGAGCGGAGTACTGTCCTC<br>CGACTCGAGCGGAGTACTGTCCTCCGATCGGAGTACTGTCCTCCGCGAATTCCG<br>GAGTACTGTCCTCCGAAGACAAGCTTAGACACTAGAGGGTATATAATGGAAGCT<br>CGACTTCCAGCTTGGCAATCCGGTATGCTGAAGAAAATCCTTAAAATAGAGGAA<br>CTTGACGAACGGGAACTGATCGACATTGAGGTTTCCGGGAATCATTTATTCTAT<br>GCAAATGATATCTTAACCCATAATAGTGGGAGCGGAGCGACCAACTTTAGCCTG<br>CTGAAACAGGCGGGCGATGTGGAAGAAAACCCTGGACCTGGTACTGTTGGTAAA<br>GCCACTATGATGCTGAAGAAAATCCTTAAAATAGAGGAACTTGACGAACGGGAA<br>CTGATCGACATTGAGGTTTCCGGGAATCATTTATTCTATGCAAATGATATCTTA<br>ACCCATAATAGTGGGAGCGGTTCTGGAAGCGGAGCGACCAACTTTAGCCTGCTG<br>AAACAGGCGGGCGATGTGGAAGAAAACCCTGGACCTGGAAGCGGAGAGGGCAGA<br>GGAAGTCTGCTAACATGCGGTGACGTCGAGGAGAATCCTGGCCCAATCGACGGT<br>GGTTCCGGAGGATCCGCCACCATGGTGAGCAAGGGCGAGGAGCTGTTCACCGGG<br>GTGGTGCCCATCCTGGTCGAGCTGGACGGCGACGTAAACGGCCACAAGTTCAGC<br>GTGTCCGGCGAGGGCGAGGGCGATGCCACCTACGGCAAGCTGACCCTGAAGTTG<br>ATCTGCACCACCGGCAAGCTGCCCGTGCCCTGGCCCACCCTCGTGACCACCCTC<br>GGCTACGGCCTGCAGTGCTTCGCCCGCTACCCCGACCACATGAAGCAGCACGAC<br>TTCTTCAAGTCCGCCATGCCCGAAGGCTACGTCCAGGAGCGCACCATCTTCTTC<br>AAGGACGACGGCAACTACAAGACCCGCGCCGAGGTGAAGTTCGATGGCGACACC<br>CTGGTGAACCGCATCGAGCTGAAGGGCATCGACTTCAAGGAGGACGGCAACATC<br>CTGGGGCACAAGCTGGAGTACAACTACAACAGCCACAACGTCTATATCACCGCC<br>GACAAGCAGAAGAACGGCATCAAGGCAAACTTCAAGATCCGCCACAACATCGAG<br>GACGGCGGCGTGCAGCTCGCCGACCACTACCAGCAGAACACCCCCATCGGCGAC<br>GGCCCCGTGCTGCTGCCCGACAACCACTACCTGAGCTACCAGTCCAAACTGAGC<br>AAAGACCCCAACGAGAAGCGCGATCACATGGTCCTGCTGGAGTTCGTGACCGCC<br>GCCGGGATCACTCTCGGCATGGACGAGCTGTACAAGGGTAGTGGTAGTGGATCC<br>TGATGGCCGGCCGCTTCGAGCAG

References

[1] Vining, K. H. & Mooney, D. J. Mechanical forces direct stem cell behaviour in development and regeneration. *Nature reviews Molecular cell biology* **18**, 728–742 (2017).

[2] Dupont, S. *et al.* Role of yap/taz in mechanotransduction. *Nature* **474**, 179–183 (2011).

[3] Ishihara, S. *et al.* Substrate stiffness regulates temporary nf-κb activation via actomyosin contractions. *Experimental cell research* **319**, 2916–2927 (2013).

[4] Swift, J. *et al.* Nuclear lamin-a scales with tissue stiffness and enhances matrix-directed differentiation. *Science* **341**, 1240104 (2013).

[5] Anastassov, S., Filo, M., Chang, C.-H. & Khammash, M. A cybergenetic framework for engineering intein-mediated integral feedback control systems. *Nature Communications* **14**, 1337 (2023).

[6] Frei, T. *et al.* Characterization and mitigation of gene expression burden in mammalian cells. *Nature communications* **11**, 4641 (2020).

[7] Lillacci, G., Benenson, Y. & Khammash, M. Synthetic control systems for high performance gene expression in mammalian cells. *Nucleic acids research* **46**, 9855–9863 (2018).

[8] Schneider, P., Willen, L. & Smulski, C. R. Tools and techniques to study ligand–receptor interactions and receptor activation by tnf superfamily members. *Methods in enzymology* **545**, 103–125 (2014).

[9] Fischer, A. A. M. *et al.* Activation of nf-κb signaling by optogenetic clustering of ikkα and β. *Advanced Biology* **9**, e00384 (2025).

[10] Reiser, J. *et al.* Transduction of nondividing cells using pseudotyped defective high-titer hiv type 1 particles. *Proceedings of the National Academy of Sciences* **93**, 15266–15271 (1996).

[11] Zhang, X.-Y., La Russa, V. F. & Reiser, J. Transduction of bone-marrow-derived mesenchymal stem cells by using lentivirus vectors pseudotyped with modified rd114 envelope glycoproteins. *Journal of virology* **78**, 1219–1229 (2004).
